# P53 – a new player in the metabolic adaptation of colorectal carcinoma cells under hypoxia

**DOI:** 10.1186/s12885-026-16184-y

**Published:** 2026-05-19

**Authors:** Stefanie Saoub, Eric Metzen, Basant Kumar Thakur, Yoshiyuki Henning, Yves Schild, Tristan Leu, Joachim Fandrey, Anna Wrobeln

**Affiliations:** 1 Institute of Physiology, University of Duisburg Essen, Hufelandstraße 55, Essen, 45147 Germany; 2 Department of Pediatrics III, University Hospital Essen, Hufelandstraße 55, Essen, 45147 Germany

**Keywords:** P53, HIF-1α, Hypoxia, Colorectal cancer, Glycolysis, Metabolic adaptation, Transcriptional regulation

## Abstract

**Background:**

Colorectal cancer (CRC) frequently exhibits hypoxic regions due to poor vascularization, leading to the stabilization of hypoxia-inducible factor 1 alpha (HIF-1α). Moreover, mutations in the tumour suppressor p53 occur in approximately half of all CRCs. While the individual roles of both transcription factors in tumour cell survival are well characterized, their interaction and its influence on the metabolic adaptation of CRC cells under hypoxic stress remain unclear.

**Methods:**

Using HCT116 CRC cells with targeted deletions of TP53 and HIF1A, we examined the effects of p53 loss on HIF-1 signalling and the respective consequences for metabolic adaptation as well as the survival of CRC cells under moderate (1% O₂) and severe (0.1% O₂) hypoxia.

**Results:**

Severe hypoxia stabilized p53 protein levels despite the transcriptional repression of *TP53*, possibly through posttranslational mechanisms and dependent on nutrient availability. In contrast to the assumption that p53 is transcriptionally inactive under hypoxia, we observed stable expression of p53 target genes (*P21*, *BAX*) under severe hypoxia, indicating functional transactivation. Loss of p53 impaired the early induction of HIF-1 target genes (*VEGF*, *PHD2*), although HIF-1α protein levels and DNA binding were unaffected, suggesting a coactivator role for p53. Furthermore, compared with wild-type cells, p53-deficient cells presented delayed but exaggerated expression of glycolytic genes, including *Glucose Uptake Transporter 1 (GLUT1)*,* Phosphofructokinase Liver-Type (PFKL) and Lactate Dehydrogenase A (LDHA)*, under hypoxia, with no impairment of glycolytic function or cell viability. Remarkably, even HIF1A knockout cells preserved glycolysis, whereas glycolytic genes were significantly downregulated, indicating HIF-1-independent metabolic compensation.

**Conclusion:**

Our findings position p53 as a temporal gatekeeper and key regulator of hypoxic adaptation in CRC cells, coordinating early gene induction and metabolic responses. The ability of CRC cells to maintain glycolysis despite the loss of p53, respectively, HIF-1α underscores the existence of compensatory HIF-independent pathways. Targeting these alternative circuits may represent a promising strategy in hypoxic, p53-deficient CRC.

**Supplementary Information:**

The online version contains supplementary material available at 10.1186/s12885-026-16184-y.

## Introduction

Colorectal cancer is the third most commonly diagnosed malignancy and the second leading cause of cancer-related death worldwide [1]. A key driver of colorectal tumourigenesis is the mutation of the tumour suppressor gene p53. Known as the “guardian of the genome”, p53 is responsible for maintaining genomic integrity by inducing cell cycle arrest, DNA repair or apoptosis in response to cellular stress. The important role of p53 in cancer development gets clear considering that colorectal carcinomas harbour mutations in the p53 gene in approximately 43% of all cases [2].

In addition to its canonical role in cell cycle regulation, p53 also contributes to metabolic control. As early as 1956, Warburg reported increased glycolytic rates in cancer cells carrying a p53 mutation [3]. Since then, there has been growing evidence that p53 promotes mitochondrial respiration, e.g., by inducing *SCO2 (synthesis of cytochrome c oxidase 2)* [4], and suppresses glycolysis through the upregulation of *TIGAR (TP53 inducible regulator of glycolysis and apoptosis) *[5] or the downregulation of *GLUT1* and *GLUT4*, two key glucose uptake transporters [6].

A key adaptive mechanism for cancer cells is their ability to survive in hypoxic environments within the tumour. For the adaptation of cell metabolism under hypoxia, hypoxia-inducible factor 1 (HIF-1), a dimeric transcription factor, is considered a central transcriptional regulator. Under low-oxygen conditions, the HIF-1α subunit escapes degradation, dimerizes with the β-subunit and activates genes involved in, inter alia, angiogenesis *(VEGF)*, erythropoiesis *(EPO)*, and glycolysis (*GLUT1*,* PFKL*, *LDHA) *[7–12].

While the interaction between HIF-1 and p53 has been extensively studied, the results remain contradictory. Some studies suggest that hypoxia stabilizes p53 [13], whereas others could not verify this effect without altering the microenvironment [14]. Similarly, examination of the impact of p53 loss on HIF-1 activity and target gene expression has led to conflicting conclusions [15–17].

Despite these efforts, it remains unclear how loss of p53 function affects metabolic adaptation under hypoxia, particularly in colorectal carcinoma cells, which naturally reside in a hypoxic environment [18]. Moreover, most studies have focused on normoxic conditions, which do not reflect the physiology of solid tumours. We therefore sought to clarify this point by our primary aim to investigate how loss of p53 affects metabolic adaptation to hypoxia in colorectal cancer cells, with a focus on the timing and regulation of HIF-1 target gene induction, the flexibility of glycolytic metabolism in the context of the Warburg effect, and the potential impact on tumour microenvironment acidification through lactate production and CA9 expression. By explicitly linking these aspects, we provide a clearer rationale for examining hypoxic adaptation and metabolic plasticity in p53-deficient colorectal cancer cells.

Accordingly, this study aimed to investigate how the loss of p53 affects the metabolic phenotype of colorectal carcinoma cells under hypoxic conditions. Specifically, we examined how p53 influences the stability and transcriptional activity of HIF-1 and how their interaction shapes glycolytic metabolism and cell viability under hypoxia. Our findings provide new insights into the metabolic plasticity of p53-deficient colorectal cancer cells and challenge the presumed central role of HIF-1 in hypoxic adaptation.

## Methods

### Cell culture

HCT116 wild-type (wt), p53 knockout (ko) and HIF1A ko cells were used for all experiments. p53 ko cells were obtained from Horizon Discovery, while HIF1A ko cells were generated in-house via lentiviral transduction using a CRISPR/Cas9 system. Lentiviral vectors based on lentiCRISPRv2-plasmid (Plasmid #52961, Addgene) were constructed. *HIF1A* knockout was generated by using 3 gRNAs with the following targeting sequences: 5’- CACCGACTTTGTCTAGTGCTTCCAT-3’, 5’- CACCGTGGTATCATATACGTGAATG-3’ and 5’- CACCGACTAAAGGACAAGTCACCAC-3’. Knockout validation is shown in supplemental Fig. 4.

All cells were cultured as monolayers in RPMI-1640 medium supplemented with 10% fetal bovine serum and 1% penicillin/streptomycin under normoxic conditions (21% O_2_, 5% CO_2_) in a humidified incubator (HeraCell, Heraeus). For the experiments, the same medium was used. Experiments with genetically modified cells were conducted within six passages. Hypoxic conditions were achieved using a hypoxia chamber (InvivO_2_ 400, Baker Ruskin).

### Experimental setup for gene and protein expression analysis as well as metabolite measurement

One day before each experiment, 1 × 10^6^ cells per well were seeded in six-well-plates (triplicates per condition) and incubated overnight under normoxia (21% O_2_, 5% CO_2_). The following day, the medium was replaced, and the cells were incubated under normoxia (21% O_2_, 5% CO_2_), moderate hypoxia (1% O_2_, 5% CO_2_) or severe hypoxia (0.1% O_2_, 5% CO_2_) for four or 24 h.

### Gene expression analysis

Total RNA was isolated by lysing cells in guanidinium thiocyanate and purifying the RNA via the NucleoSpin RNA Kit (MACHERY-NAGEL) according to the manufacturer’s instructions. 500 ng of total RNA were reverse-transcribed into complementary DNA (cDNA) using oligo(dT) primers, with primer annealing at 68 °C for 10 min, followed by cDNA synthesis with M-MLV reverse transcriptase (Promega) in a three-step program (90 min at 45 °C, 30 min at 52 °C and 30 min at 90 °C) in a TECHNE™ 3PrimeG Thermocycler (Thermo Fisher Scientific). Quantitative real-time PCR was performed using Biozym Blue SyBr Green 2x qPCR Mix and gene-specific primers. The primers listed in Table 1 were used.

**Table 1:** Human primer sequences

	TCACTGGGACTATTAGGCTCAGGT	forward 5`	202 bp
HIF1A	CTCCATTACCCACCGCTGAA	reverse 3`	
*VEGF*	GCAAGACAAGAAAATCCCTGTGGGCC	forward 5`	165 bp
	CCGCCTCGGCTTGTCACA	reverse 3`	
*PHD2*	CCAGCTTCCCGTTACAGT	forward 5`	176 bp
	GCACGACACCGGGAAGTT	reverse 3`	
*CA9*	CAC GTG GTT CAC CTC AGC AC	forward 5`	151 bp
	CAG CGA TTT CTT CCA AGC G	reverse 3`	
*GLUT1*	TCTGGCATCAACGCTGTCTT	forward 5`	214 bp
	CTAGCGCGATGGTCATGAGT	reverse 3`	
*PFKL*	GCTGGGCGGCACTATCATT	forward 5`	236 bp
	TCAGGTGCGAGTAGGTCCG	reverse 3`	
*LDHA*	TTGACCTACGTGGCTTGGAAG	forward 5`	91 bp
	GGTAACGGAATCGGGCTGAAT	reverse 3`	
*p53*	AGACCCAGGTCCAGATGAAGCT	forward 5`	161 bp
	GGAAACCGTAGCTGCCCTGGTA	reverse 3`	
*BAX*	CCCGAGAGGTCTTTTTCCGAG	forward 5`	155 bp
	CCAGCCCATGATGGTTCTGAT	reverse 3`	
*P21*	TGTCCGTCAGAACCCATGC	forward 5`	139 bp
	AAAGTCGAAGTTCCATCGCTC	reverse 3`	
*MDM2*	TCGTCGGGTGAGGGTACTG	forward 5`	152 bp
	AACCACTTCTTGGAACCAGGT	reverse 3`	
*ACTIN*	TCACCCACACTGTGCCCATCTACGA	forward 5`	295 bp
	CAGCGGAACCGCTCATTGCCAATGG	reverse 3`	

### Protein expression analysis

The cells were washed with ice-cold PBS and lysed in buffer which contained 150 mM NaCl, 20 mM Tris-HCl, 1% Nonidet P-40, 5 mM EDTA and was supplemented with protease inhibitor (1:10) or, for p53 phosphorylation analysis, protease/phosphatase inhibitor (1:100). The lysates were incubated on ice for 30 min and centrifuged for 15 min at 13,200 rpm at 4 °C. The supernatants were stored at −80 °C. The protein concentration was determined by the Lowry method. 30–40 µg of the total protein amount was denatured with SDS, separated on 7.5% SDS‒PAGE gels, and then transferred to PVDF membranes via a TurboBlot system (Bio-Rad). The membranes were blocked for at least 1 h at room temperature in 5% nonfat dry milk or for analysis of phosphorylated p53 protein in 5% bovine serum albumin in TBS-T. The membranes were incubated overnight at 4 °C with primary antibodies (Table 2) in blocking solution. After three washes in TBS-T, the membranes were incubated with HRP-conjugated secondary antibodies (1:10000) in blocking solution for one hour at room temperature. Chemiluminescence detection was performed after the addition of HRP substrate to the membrane. For sequential protein detection on the same membrane, the membranes were stripped with Restore™ Western Blot Stripping Buffer (Thermo Scientific Fisher) for 30 min, washed and then reblocked before the procedure described above was repeated.

**Table 2:** Primary antibodies used for western blot analysis and immunohistochemistry

HIF-1α	BD Transduction Laboratories™
α-Tubulin	Santa Cruz Biotechnology
P53	Proteintech
p-P53	Proteintech
ARNT	Cell Signaling Technology

### Immunohistochemistry

Two days before the experiment, 120,000 cells per well were seeded on cover slips in 24-well-plates. After 24 h under normoxia or moderate or severe hypoxia, cells were fixed in precooled (−20 °C) methanol/acetone, washed three times with PBS, and permeabilized with 0.5% Triton X-100 in PBS for 15 min. After further washing with PBS-T (0.1% Triton X-100 in PBS), the coverslips were blocked with 10% donkey serum in PBS for one hour at room temperature. Primary antibodies (p53, Proteintech) were diluted 1:100 in PBS-T with 0.5% donkey serum and incubated overnight at 4 °C in the dark. The following day, the cover slips were washed and incubated with an Alexa Fluor 568-conjugated secondary antibody (1:100) for one hour at room temperature. After washing, the cover slips were mounted with Mowiol containing DAPI (1:1000).

### HIF-1 binding activity

A total of 10 million HCT116 p53 wt and p53 ko cells were seeded in TC75 flasks (duplicates per condition). The next day, the medium was replaced, and cells were exposed to normoxia or moderate or severe hypoxia for four hours. Nuclear protein extracts were prepared via a Nuclear Extraction Kit (Cayman Chemical) following the manufacturer’s protocol. The purity of the nuclear extracts was confirmed via Western blot analysis of α-tubulin. The HIF-1α protein concentration in the nuclear extracts was quantified by means of a HIF1A ELISA (Human HIF1A ELISA Kit, Invitrogen). HIF-1 binding activity to the HRE was assessed via a HIF-1α Transcription Factor Assay Kit (Cayman Chemical). 40 fg of HIF-1α protein were loaded per well; in normoxic samples with low HIF-1α protein amount, the highest volume determined from hypoxic samples was used. Assays were performed according to the manufacturers’ protocols with appropriate positive and negative controls.

### Glucose and lactate measurement

The medium was collected after 24 h of exposure to normoxia or moderate or severe hypoxia and analysed in a blood gas analyser (ABL 715, Radiometer).

### Extracellular flux analysis

Extracellular flux analyses were performed in a Seahorse XFe24 Analyzer (Agilent). A total of 50,000 HCT116 wt, p53 ko and HIF1A ko cells were seeded per well in XFe24 cell culture plates. The plates were shaken vigorously to achieve a homogenous cell monolayer. As HCT116 cells reached complete adherence after six hours at room temperature, cells were dispensed in cell culture medium supplemented with 25 nM HEPES. Cells were incubated under normoxia or moderate hypoxia (1% O_2_) overnight. Sensor cartridges were hydrated in XF Calibrant overnight under CO_2_-free normoxic or hypoxic conditions. The following day, cells were washed and incubated for one hour in Agilent Seahorse XF RPMI medium (pH = 7.4) supplemented with 10 mM glucose, 2 mM glutamine, and 1 mM pyruvate (Glycolysis Stress Test medium contained only 2 mM glutamine) under CO_2_-free normoxic or hypoxic conditions. The oxygen consumption rate (OCR) and extracellular acidification rate (ECAR) were measured via the Mito Stress Test (1 µM oligomycin, 1 µM FCCP, 0.5 µM rotenone/antimycin A), the Glycolysis Stress Test (2 mM glucose, 50 mM 2-deoxyglucose, 0.5 µM rotenone/antimycin A) and the Glycolytic Rate Assay (50 nM 2-deoxyglucose, 0.5 µM rotenone/antimycin A). Normalisation was achieved by cell staining with Hoechst 33,342 (1 µg/ml). Hypoxic Seahorse XF assays were performed in a dedicated hypoxia chamber at 1% O₂ to ensure continuous hypoxic conditions during the measurement, preventing reoxygenation and capturing the metabolic state of the cells under hypoxia.

### Annexin assay

A total of 200,000 cells per well were seeded in 12-well-plates and incubated overnight under normoxia. The next day, the medium was changed, and cells were incubated for 24 h under normoxia (21% O_2_) or severe hypoxia (0.1% O_2_). Afterwards, cells were detached with 300 µl of Accutase per well (for 5 min), neutralized with 700 µl of medium, and kept on ice. After centrifugation at 1000 rpm for 5 min and washing twice with ice-cold PBS, cells were resuspended in 200 µl of binding buffer. 5 µl FITC Annexin V and 5 µl of propidium iodide (PI) were added to each sample. After 15 min of incubation at room temperature in the dark, the samples were analysed on a BD FACSCelesta™ Multicolor Flow Cytometer. Colchicine-treated cells (40 nM) served as a positive control for FITC-conjugated Annexin V, and heat-denatured cells served as a positive control for PI staining. The data were analysed via FlowJo.

### Statistics

Statistical analyses were performed via GraphPad Prism 10. The outliers were removed via Grubbs’ test (α = 0.05). Normality was assessed via the Shapiro‒Wilk test. For normally distributed data, unpaired t tests were performed. In the case of unequal variance, a t test with Welch’s correction was applied. For data that were not distributed normally, the Mann‒Whitney U test was chosen. All the data are expressed as mean + SD. P values are expressed as absolute numbers. n refers to biological replicates, N to technical replicates.

## Results

### Severe hypoxia restores p53 protein levels despite strong mRNA downregulation

**Fig. 1:**
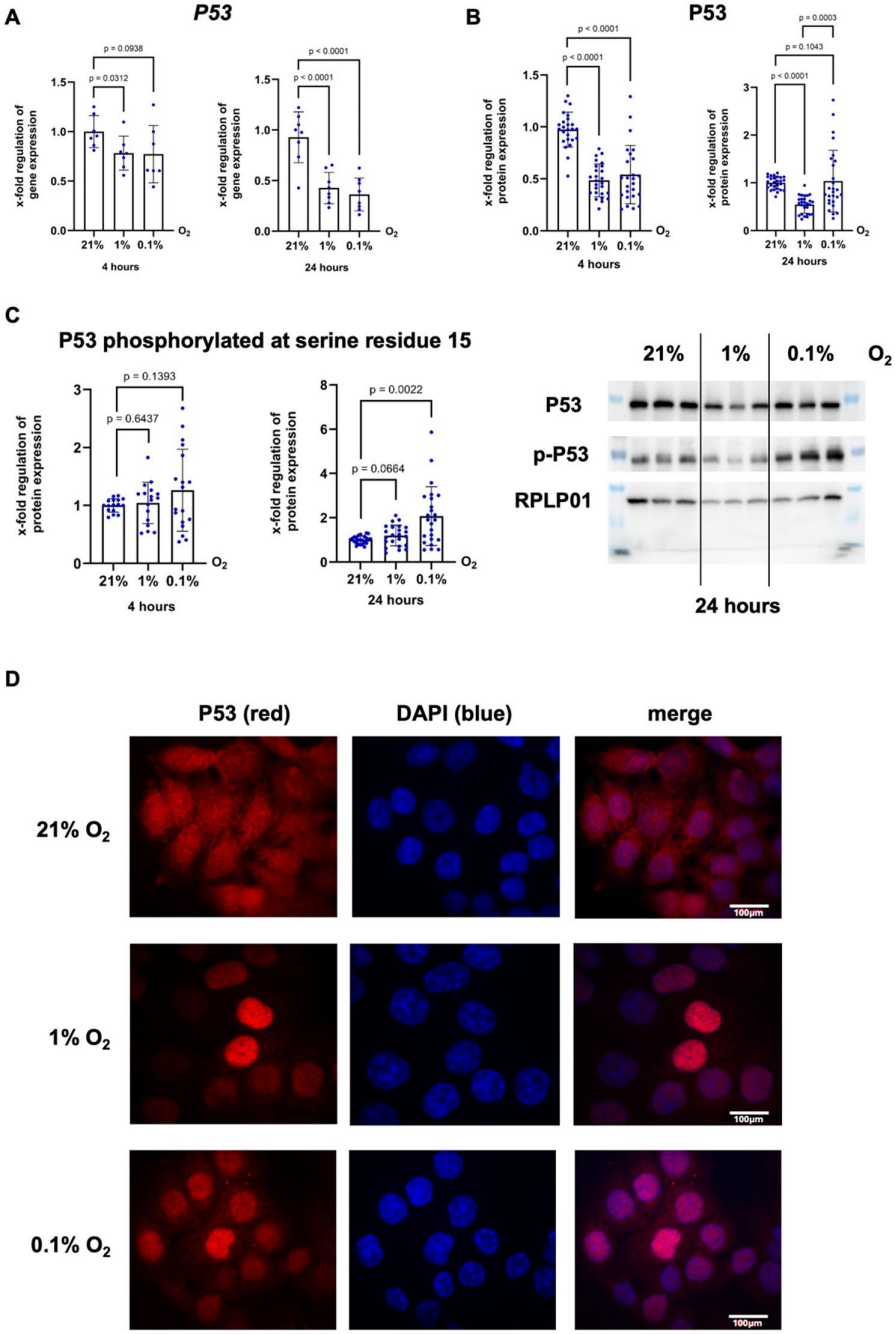
P53 protein recover under severe hypoxia despite mRNA suppression. **A** qPCR analysis of p53 mRNA expression (*n* = 7–8, *N* = 19–21) and (**B**) Western Blot analysis of p53 protein expression (technical replicates presented, *n* = 11, *N* = 25–27) after four and 24 h under normoxia, moderate (1% O_2_) and severe (0.1% O_2_) hypoxia. **C** Western Blot analysis of phosphorylation status of p53 in HCT116 wt cells after 24 h under moderate and severe hypoxia compared to normoxia (technical replicates presented, *n* = 8, *N* = 24). **D** Immunohistochemical analysis showing nuclear accumulation of p53 under moderate and severe hypoxia compared to normoxia. HCT116 cells were stained with Alexa Fluor 568 (red), which bound to a p53 antibody, and DAPI (blue) as nuclear stain, *n* = 3, *N* = 9

To clarify how the tumour suppressor p53 is regulated under hypoxic conditions, we analysed p53 mRNA and protein levels under normoxia (21% O₂), moderate hypoxia (1% O₂), and severe hypoxia (0.1% O₂) after incubation for 4 and 24 h. As shown in Fig. 1A, P53 mRNA expression was significantly downregulated under both hypoxic conditions compared to normoxia. Interestingly, after 24 h, p53 protein levels recovered under severe hypoxia, whereas they remained suppressed under moderate hypoxia (Fig. 1B). These data suggest that prolonged severe hypoxia can restore p53 protein levels despite transcriptional downregulation.

To determine whether posttranslational modifications contribute to p53 protein stabilization under hypoxia, we examined the phosphorylation status of the p53 protein at serine residue 15 (Ser15), a modification known to protect the protein from degradation. Indeed, phosphorylation of p53 at Ser15 was significantly increased after 24 h of severe hypoxia compared to normoxia, whereas moderate hypoxia did not significantly change p53 phosphorylation (Fig. 1C). However, the comparison between moderate hypoxia and normoxia yielded a p value of 0.0664, indicating a trend towards increased phosphorylation already under moderate hypoxia. In line with these findings, immunofluorescence staining indicated nuclear accumulation of p53 after 24 h of both moderate and severe hypoxia, suggesting that this transcription factor is generally activated under hypoxic conditions (Fig. 1D).

### p53 is required for maintaining *BAX* expression under severe hypoxia

**Fig. 2:**
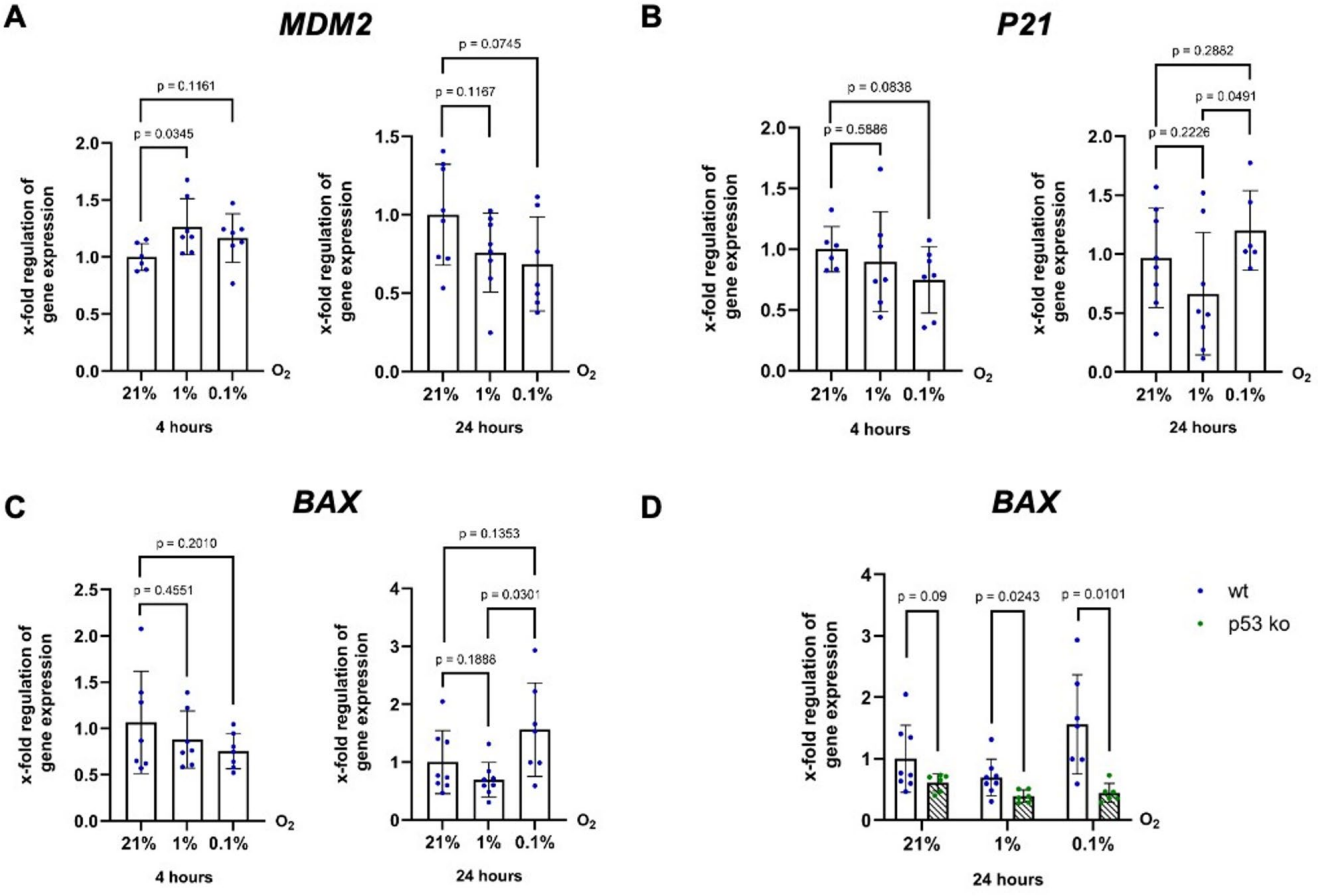
BAX induction under severe hypoxia depends on P53. Expression of p53 target genes *MDM2* (**A**), *P21* (**B**) and *BAX* (**C**) in HCT116 wt cells after four and 24 h under moderate and severe hypoxia compared to normoxia. n (wt) = 6–8, N (wt) = 16–24 (**D**) mRNA expression of the specific p53 target gene *BAX* in HCT116 p53 wt and p53 ko cells after 24 h under moderate and severe hypoxia compared to normoxia. In C and D Data are derived from the same experiment, WT data are shared between panels for direct comparison. *n* = 6–7 n (wt) = 7–8, N (wt) = 18–24, n (p53 ko) = 6, N (p53 ko) = 18

Next, we analysed the mRNA expression of three established p53 target genes, namely, *Mouse Double Minute 2 Homolog (MDM2)*, *BCL2 Associated X (BAX)* and *Cyclin Dependent Kinase Inhibitor 1 A (CDKN1A)*, also known as *P21*. MDM2 induces the degradation of p53 for negative feedback regulation. BAX is a proapoptotic molecule, whereas P21 induces cell cycle arrest. No significant changes in the mRNA expression of these genes were detected after 24 h of severe hypoxia compared to normoxia (Fig. 2). However, both *P21* (Fig. 2B) and *BAX* (Fig. 2C) mRNA expression were significantly upregulated after 24 h of severe hypoxia compared to moderate hypoxia. This difference between the expression levels of *BAX* after 24 h under moderate and severe hypoxia could not be observed in HCT116 p53 ko cells, suggesting that p53 is required for fine-tuning the regulation of these genes under hypoxic stress (Fig. 2D).

### P53 knockout does not influence the expression of HIF-1α but might reduce the transcriptional activity of HIF-1

**Fig. 3:**
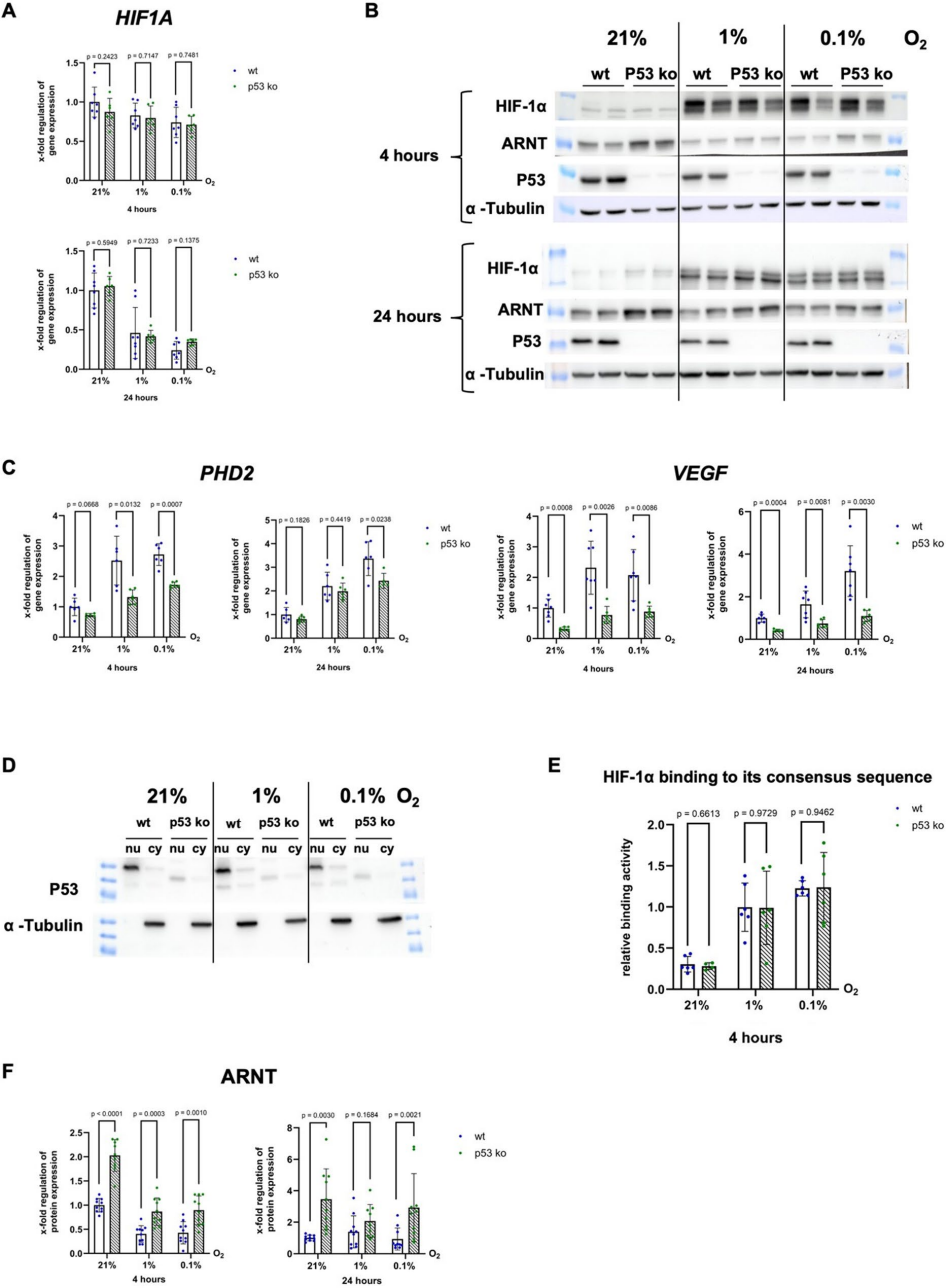
Loss of p53 does not alter HIF-1α protein levels but diminishes its transcriptional activity. **A** qPCR analysis of mRNA-expression of *HIF1A* in HCT116 p53 wt and ko cells after four and 24 h under normoxia (21% O_2_), moderate (1% O_2_) and severe (0.1% O_2_) hypoxia. n (wt) = 5–8, N (wt) = 19–24. **B** Western Blot analysis of HIF-1α (*n* = 3, *N* = 6) and ARNT (n (wt/p53 ko) = 5, N (wt/p53 ko) = 10) protein in HCT116 p53 wt and ko cells after four and 24 h under normoxia, moderate and severe hypoxia. **C** qPCR analysis of mRNA-expression of HIF1-targets *PHD2* and *VEGF* in HCT116 p53 wt and ko cells after four and 24 h under normoxia, moderate and severe hypoxia. n (wt) = 6–7, N (wt) = 17–21, n (p53ko) = 5–6, N (p53 ko) = 15–18 (**D**) Western Blot analysis to check purity of nuclear extracts (nu) generated from HCT116 p53 wt and ko cells after four hours under normoxia, moderate and severe hypoxia. cy = cytosol extracts (**E**) HIF-1α transcription factor assay measuring binding activity of HIF-1α after four hours in HCT116 p53 wt and ko cells under moderate and severe hypoxia compared to a positive control. n (wt/p53 ko) = 3, N (wt) = 6, N (p53 ko) = 4–6. **F** Western Blot analysis of ARNT protein expression in HCT116 p53 wt and ko cells after four und 24 h under normoxia, moderate and severe hypoxia. n (wt/p53 ko) = 5, N (wt/p53 ko) = 10

We next examined whether the loss of p53 affects HIF-1α expression or activity, which is crucial for metabolic changes and cell survival under hypoxic conditions. A p53 knockout affected neither the mRNA (Fig. 3A) nor the protein (Fig. 3B) expression of HIF-1α. However, we observed significantly lower mRNA expression levels of the HIF-1 target genes *Prolyl Hydroxylase 2 (PHD2)* and *Vascular Endothelial Growth Factor (VEGF)* (Fig. 3C) in HCT116 p53 ko cells than in HCT116 p53 wt cells after four hours of incubation under hypoxia, suggesting impaired HIF-1-mediated transcription.

To assess HIF-1 binding activity at its consensus sequence, the hypoxia‒response element (HRE), we isolated nuclear extracts from HCT116 p53 wt and p53 ko cells after four hours of incubation under normoxia, moderate and severe hypoxia. The purity of the nuclear extracts was checked via Western blotting (Fig. 3D). A HIF-1α ELISA was subsequently used to quantify the HIF-1α concentration of every sample so that we were able to apply the same amount of HIF-1α protein on an ELISA plate, on the bottom of which the HRE oligonucleotide was immobilized. No significant differences in HRE-binding capacity were observed between HCT116 p53 wt and ko cells (Fig. 3E). Given these findings, we assessed whether downregulation of the ARNT protein, a crucial dimerization partner of HIF-1α, might be the reason for the reduced transcriptional activation of HIF-1 targets. However, a p53 knockout even led to a significant increase in ARNT protein levels, ruling out ARNT deficiency as a cause of reduced target gene activation (Fig. 3F).

### Compared to p53 wt cells, p53 ko cells more strongly upregulate glycolytic target genes of HIF-1 after 24 h of hypoxia

**Fig. 4:**
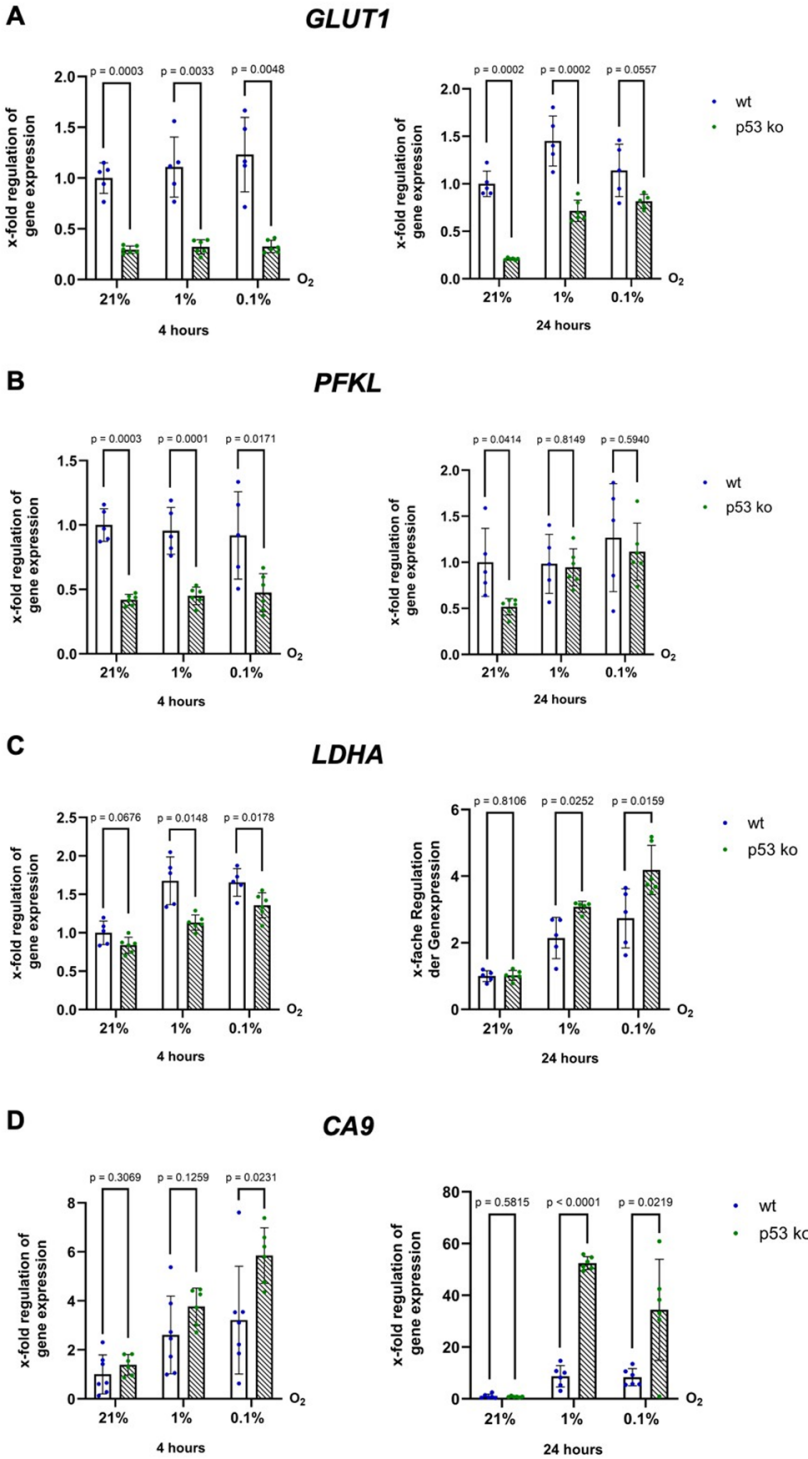
Enhanced induction of glycolytic HIF-1 targets in p53 ko cells under hypoxia. qPCR analysis of mRNA expression of HIF-1 glycolytic target genes (**A**) *GLUT1*, (**B**) *PFKL*, (**C**) *LDHA* and (**D**) *CA9* in HCT116 p53 wt and ko cells after four and 24 h under normoxia (21% O_2_), moderate (1% O_2_) and severe (0.1% O_2_) hypoxia. n (wt) = 6–8, N (wt) = 16–24, n (p53 ko) = 5–6, N (p53 ko) = 15–18

We then investigated the mRNA expression of metabolism related HIF-1 target gene *GLUT1* (Fig. 4A), an important glucose uptake transporter often upregulated in colorectal carcinomas; *PFKL* (Fig. 4B), the key enzyme of glycolysis; *LDHA* (Fig. 4C), which is responsible for metabolizing pyruvate to lactate in the context of anaerobic glycolysis; and *Carbonic Anhydrase IX (CA9)*, which contributes to maintaining acid‒base balance and thus fulfils an important role in the metabolism of cancer cells (Fig. 4D). After four hours of hypoxia, all genes except *CA9* were significantly downregulated in p53 ko cells compared to wt cells. Remarkably, after 24 h under hypoxia, p53 ko cells demonstrated robust upregulation of *GLUT1*, *PFKL*, *LDHA* and *CA9*, reaching or exceeding the expression levels in wt cells. This finding suggests a delayed but potent compensatory response in the absence of p53, which specifically targets glycolytic pathways, as *PHD2* and *VEGF* did not exhibit similar dynamics (Fig. 3C).

### A p53 knockout does not impair hypoxic glycolysis

**Fig. 5:**
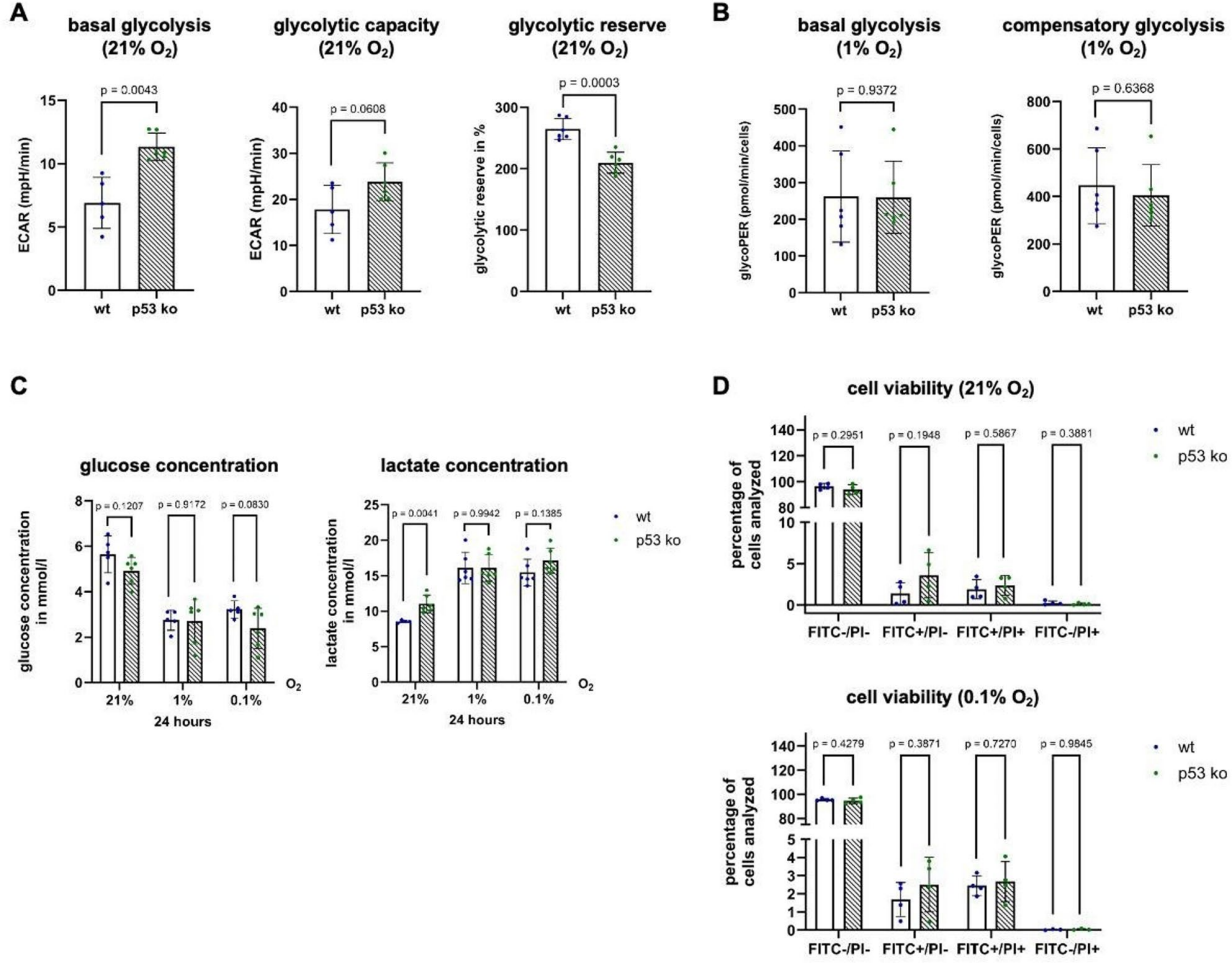
Glycolytic activity remains intact in p53 ko cells under hypoxia. **A** Glycolysis Stress Test of HCT116 p53 wt and ko cells under normoxia (21% O_2_). n (wt) = 5–6, N (wt) = 19–21, n (p53 ko) = 6, N (p53 ko) = 17–18 (**B**) Glycolytic Rate Test of HCT116 p53 wt and ko cells under moderate (1% O_2_) hypoxia. n (wt) = 6, N(wt) = 18, n (p53 ko) = 6, N (p53 ko) = 15 (**C**) Measurement of glucose and lactate concentration in the medium of HCT116 p53 wt and ko after 24 h under normoxia, moderate and severe (0.1% O_2_) hypoxia. n (wt) = 5–6, N (wt) = 15–18, n (p53 ko) = 6, N (p53 ko) = 18 (**D**) Annexin assay analysing apoptotic cells by binding to externalized phosphatidylserine. A decrease in Annexin V-negative cells is commonly interpreted as a reduction in the viable cell population. Cell viability of HCT116 p53 wt and ko cells analysed after 24 h under normoxia and severe hypoxia. n (wt/p53 ko) = 4, N (wt/p53 ko) = 4

To determine whether the potential reduction in the transcriptional activity of HIF-1 as a consequence of p53 loss might impair its glycolytic metabolism under hypoxia, we performed extracellular flux assays under normoxia as well as moderate hypoxia (1% O_2_). Under normoxia, we detected significantly higher basal glycolytic rates in HCT116 p53 ko cells than in HCT116 p53 wt cells. As the glycolytic capacity was similar compared to that of p53 wt cells, a p53 Knockout resulted in a reduced glycolytic reserve (Fig. 5A). Under hypoxia, no significant differences in glycolytic rates were observed between p53 wt and ko cells (Fig. 5B). These results were reflected in the measurements of the glucose and lactate concentrations in the medium of the cells after 24 h of incubation under normoxia, moderate and severe hypoxia (Fig. 5C). In addition, cell viability remained unaffected by p53 status under severe hypoxia (Fig. 5D).

### Preservation of glycolytic function in HIF1Ako colorectal carcinoma cells under hypoxia

**Fig. 6:**
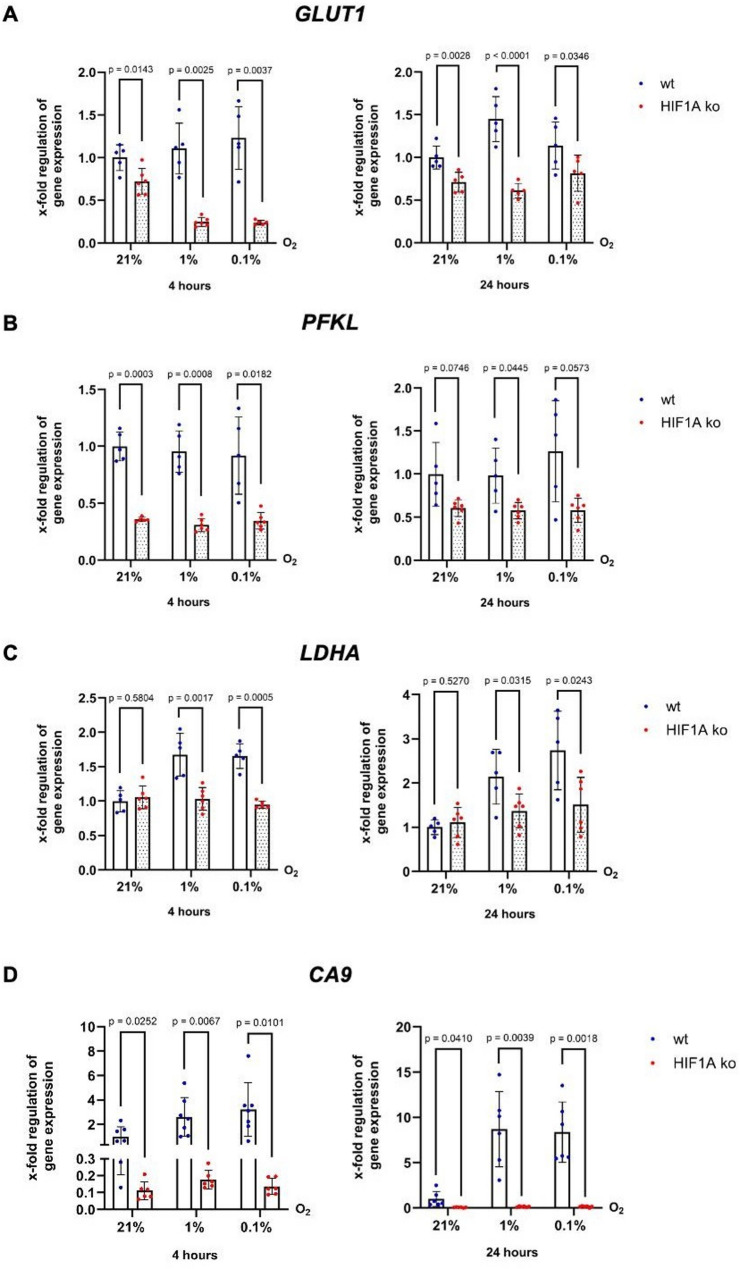
mRNA expression of HIF-1 target genes was significantly reduced in HIF1A ko cells. qPCR analysis of mRNA expression of HIF-1 glycolytic target genes (**A**) *GLUT1*, (**B**) *PFKL*, (**C**) *LDHA* and (**D**) *CA9* in HCT116 HIF1A wt and ko cells after four and 24 h under normoxia (21% O_2_), moderate (1% O_2_) and severe (0.1% O_2_) hypoxia. n (wt) = 6–8, N (wt) = 16–24, n (HIF1A ko) = 5–6, N (HIF1A ko) = 15–18

As reduced expression of glycolytic genes at the beginning of the hypoxic phase did not impair glycolytic metabolism in p53 ko cells compared to wt cells, we aimed to analyse the influence of a *HIF1A* knockout on the metabolic adaptation of colorectal carcinoma cells to hypoxia. Therefore, we generated HCT116 HIF1A ko cells with the help of a CRISPR/Cas system and achieved a knockout efficiency of over 99% at the mRNA level (Supplemental Fig. 1). Consequently, the mRNA expression of all the analysed HIF-1 target genes, including the glycolytic genes *GLUT1*, *PFKL* and *LDHA*, was significantly lower under hypoxia in HCT116 HIF1A ko cells than in HIF1A wt cells (Fig. 6).

**Fig. 7:**
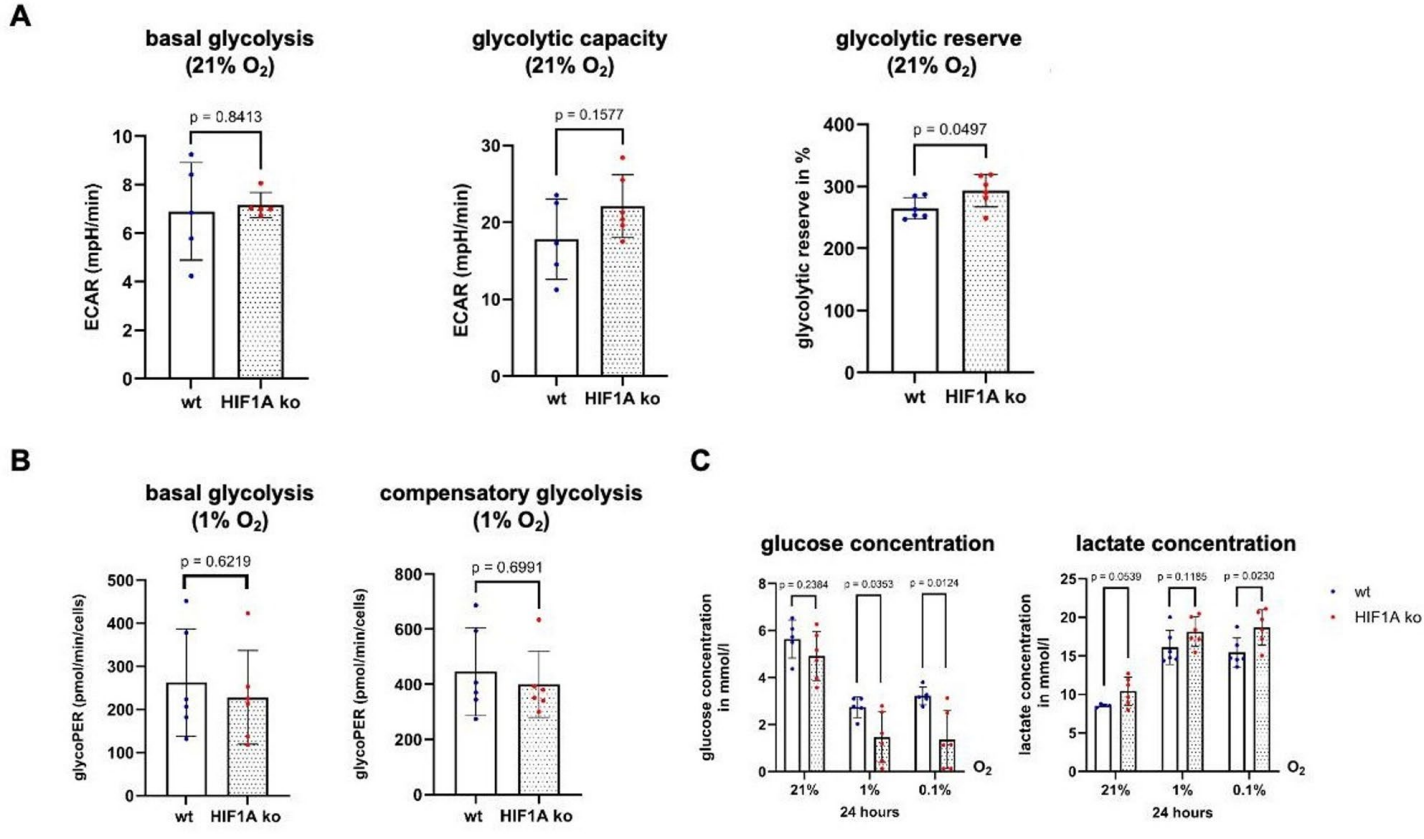
Glycolytic activity under hypoxia is maintained in colorectal carcinoma cells despite HIF-1α loss. **A** Glycolysis Stress Test of HCT116 HIF1A wt and ko cells under normoxia (21% O_2_). n (wt) = 5–6, N (wt) = 19–21, n (HIF1A ko) = 5–6, N (HIF1A ko) = 18–21 (**B**) Glycolytic Rate Test of HCT116 HIF1A wt and ko cells under moderate (1% O_2_) hypoxia. n (wt) = 6, N (wt) = 18, n (HIF1A ko) = 6, N (HIF1A ko) = 18 (**C**) Measurement of glucose and lactate concentration in the medium of HCT116 HIF1A wt and ko cells after 24 h under normoxia, moderate and severe (0.1% O_2_) hypoxia. n (wt) = 5–6, N (wt) = 15–18, n (HIF1A ko) = 6, N (HIF1A ko) = 17–18

Surprisingly, despite the inability of HCT116 HIF1A ko cells to upregulate the mRNA expression of glycolytic target genes under hypoxia, we could not recognize any restriction of glycolytic metabolism under normoxic (Fig. 7A) and hypoxic conditions (Fig. 7B), suggesting compensatory metabolic rewiring independent of HIF-1α. Compared to HCT116 HIF1A wt cells, HCT116 HIF1A ko cells even presented greater glucose consumption and lactate production after 24 h under hypoxia (Fig. 7C).

### Nutrient deficiency increases apoptotic cell death and p53 stabilization under hypoxia

**Fig. 8:**
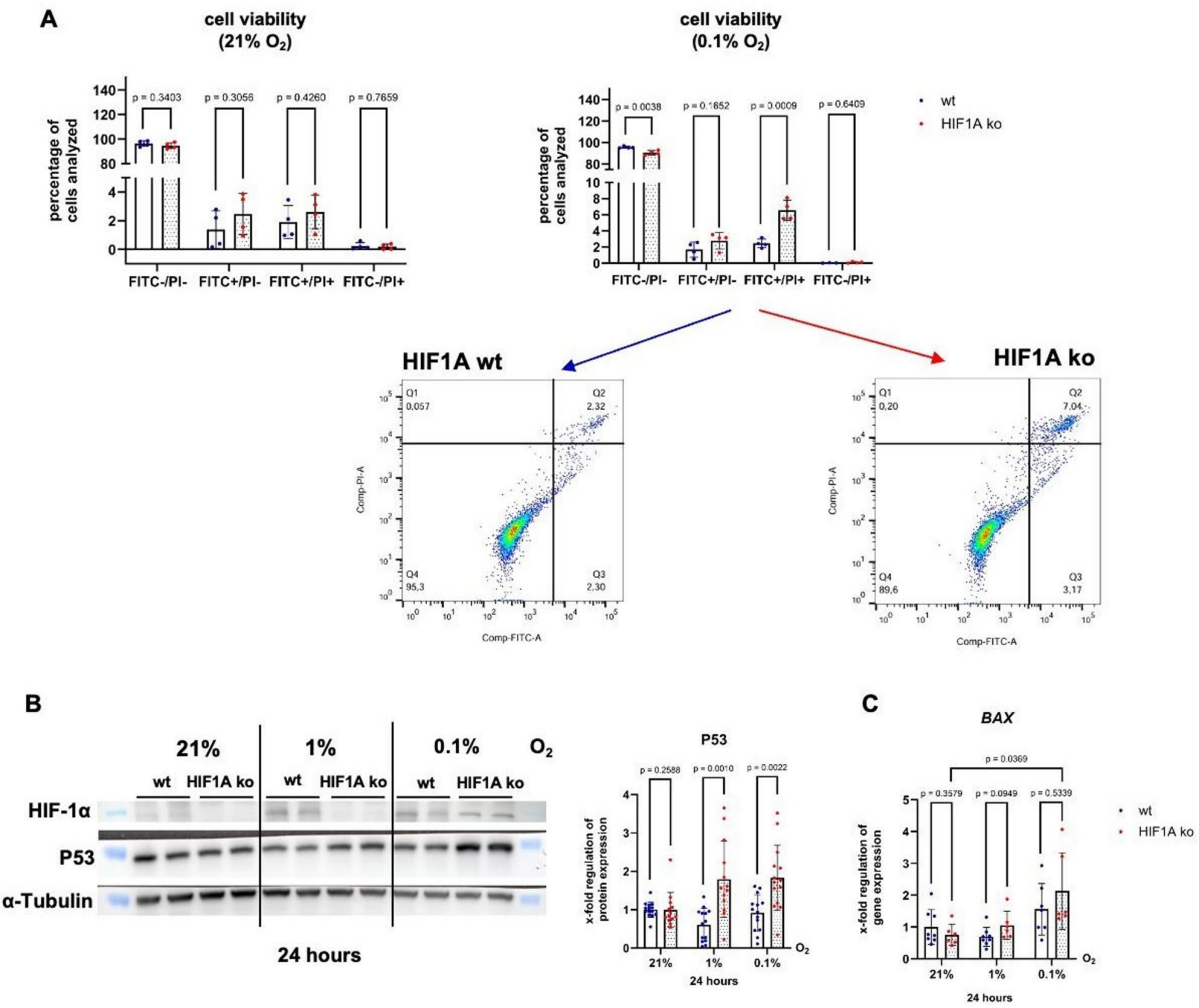
Nutrient deprivation enhances apoptosis and stabilizes p53 under hypoxia. **A** Annexin assay analysing apoptotic cells. A decrease in Annexin V-negative cells is commonly interpreted as a reduction in the viable cell population.HCT116 HIF1A wt and ko cells analysed after 24 h under normoxia (21% O_2_) and severe hypoxia (0.1% O_2_). n (wt/HIF1A ko) = 4, N (wt/HIF1A ko) = 4 (**B**) Western Blot analysis of p53 protein levels in HCT116 HIF1A wt and ko cells after 24 h under normoxia, moderate and severe hypoxia (technical replicates represented, n (wt/HIF1A ko) = 7, N (wt) = 13–14, N (HIF1A ko) = 14) (**C**) qPCR analysis of *BAX* mRNA expression in HCT116 HIF1A wt and ko cells after 24 h under normoxia, moderate and severe hypoxia. n (wt) = 7–8, N (wt) = 21–24, n (HIF1A ko) = 6, N (HIF1A ko) = 18

Given the low glucose levels in the medium of HCT116 HIF1A ko cells after 24 h of hypoxia in comparison to those in the medium of the HCT116 HIF1A wt cells, we wondered whether cell viability might be impaired. Flow cytometric analysis revealed no significant changes in cell viability under normoxia. Under severe hypoxia, a modest but significant increase in apoptosis was observed in HCT116 HIF1A ko cells compared to HCT116 wt cells (Fig. 8A). This increase was accompanied by elevated p53 protein levels (Fig. 8B) and increased *BAX* mRNA expression under hypoxic conditions (Fig. 8C), indicating stress-induced activation of the p53 pathway in the absence of HIF-1α.

## Discussion

This study demonstrated that p53 plays a multifaceted and context-dependent role in modulating the cellular response to hypoxia, with distinct effects depending on the severity and duration of oxygen deprivation. Most notably, we showed that severe hypoxia (0.1% O₂) restored p53 protein levels (Fig. 1B) despite the transcriptional repression of *TP53* (Fig. 1A), possibly through posttranslational mechanisms. Immunofluorescence analysis (Fig. 1D) indicated nuclear accumulation of p53 under hypoxia. However, we cannot distinguish increased total p53 protein from enhanced nuclear translocation in the absence of nuclear fractionation. This finding supports the hypothesis of Koumenis et al. [19] that cellular stress under extreme hypoxia activates p53 as a stress sensor via ATM/ATR signalling and Ser15 phosphorylation (Fig. 1C) [13]. However, for activation of the ATM kinase DNA breakage is necessary. Hammond et al. [20] did not detect DNA breakage in RKO cells under severe hypoxia, though replication arrest leading to activation of the ATR kinase [13]. Hence, the upstream signals driving p53 Ser15 phosphorylation under severe hypoxia remain incompletely defined. In particular, activation of ATM can be triggered by DNA damage or mitochondrial reactive oxygen species (ROS), both of which have been linked to hypoxic stress. In the present study, we did not directly assess ATM activation, DNA damage markers, or mitochondrial ROS levels. To further address this point, we analyzed the mRNA expression of *ATR* and *ATM*. While *ATR* expression was significantly downregulated under hypoxia, *ATM* mRNA levels remained unchanged (Supplemental Fig. 2). These findings do not provide evidence for a transcriptional upregulation of canonical DNA damage signalling pathways, although effects at the level of kinase activation cannot be excluded. In addition, metabolic flux analyses revealed reduced mitochondrial activity under hypoxic conditions (Supplemental Fig. 3). While decreased mitochondrial respiration under hypoxia could potentially limit overall ROS production, hypoxia-associated alterations in electron transport chain efficiency have also been reported to promote mitochondrial ROS generation [21, 22]. Therefore, a contribution of mitochondrial ROS or DNA damage signalling to p53 stabilization cannot be excluded. These considerations highlight an important limitation of our study and indicate that future work will be required to directly assess the involvement of ATM activation, DNA damage, and ROS in hypoxia-induced p53 regulation. Furthermore, compensatory downregulation of the mRNA expression of *TP53* in response to increased p53 protein stabilization must be considered.

Importantly, the kinetics of p53 mRNA and protein stabilization in HCT116 cells differ from those in previously described models, such as MCF-7 cells and MEFs. Our study provides a novel quantitative and time-resolved analysis of p53 dynamics specifically in colorectal carcinoma cells, thereby extending previous observations. For example, Koumenis et al. (2001) reported early stabilization of the p53 protein after 8 h under severe hypoxia in MCF-7 cells, as well as hypoxic p53 mRNA expression levels, which were similar to normoxic levels. However, our study focused on colorectal carcinoma cells, which are highly resistant to severe hypoxia, as our cell viability analysis indicated (Fig. 5C). There are several working groups who were not able to detect hypoxic p53 protein stabilization in multiple cell lines [14, 23, 24]. Consistent with these findings, we observed that p53 protein stabilization in HCT116 cells under severe hypoxia was highly variable. Specifically, p53 protein levels were in some cases lower and in others higher than those observed under normoxic conditions, resulting overall in average levels that did not differ from normoxia. As described further below, p53 is able to support cell survival in situations of mild stress. Irrecoverable cell damage, however, activates p53 as promoter of cell death. The inability to find p53 stabilization under hypoxia might point out that the hypoxic stress is not high enough to produce irreversible cell damage. This hypothesis could explain why Pan et al. could only observe increased p53 protein accumulation in HCT116 cells under severe hypoxia after increasing cell density. Taken together, these findings emphasize that generalizations regarding p53 stabilization under hypoxia should be made cautiously. We want to point out that, factors such as cell density and nutrient availability are critical for p53 stabilization, in addition to the robustness of the cell type against hypoxia [14]. Nutrient depletion can activate p53 via different molecular pathways (e.g., the AMPK pathway), as summarized by Humpton and Vousden [25]. Similarly, compared to HIF1A wt cells, HCT116 HIF1A ko cells, which presented lower glucose concentrations after 24 h under hypoxia (Fig. 7C), also showed higher p53 protein levels (Fig. 8B) and increased apoptotic cell death rates (Fig. 8A).

It has been proposed that, under hypoxia, p53 exhibits reduced canonical transactivation activity and may preferentially mediate stress responses through transrepression [19, 20, 26]. However, our data challenge a strictly transcriptionally inactive model of p53 under hypoxia. We demonstrated that, in HCT116 cells, p53 protein stabilization following 24 h of severe hypoxia was associated with the upregulation of canonical target genes such as *P21* and *BAX* compared to moderate hypoxia (Fig. 2). These findings indicate that p53 remains transcriptionally active and contributes to cell fate decisions in oxygen-deprived environments. These findings align with previous reports that described p53 as a molecular switch balancing survival and apoptosis under hypoxic stress [25, 27]. The complete loss of this transcriptional activation in p53 knockout cells confirms that this response is p53-dependent (Fig. 2D). While a role for transrepression of genes by p53 cannot be excluded, our findings indicate that p53 retains significant transcriptional function under hypoxia.

Although HCT116 cells are resistant to hypoxia-induced apoptosis, a p53 knockout did not improve survival under hypoxic conditions (Fig. 5D). This indicates that p53 is not the dominant determinant of survival in this context when an intact HIF-1 mediated adaptive response is present. Importantly, this observation does not contradict the increased apoptotic cell death observed in HIF-1α-deficient cells (Fig. 8), as these experimental settings reflect distinct biological contexts. While p53 knockout cells retain functional HIF-1 dependent adaptive signalling, HIF-1α-deficient cells lack a central hypoxia response pathway, resulting in increased cellular stress and enhanced activation of p53-associated apoptotic programs. Thus, HIF-1α primarily supports cellular adaptation and survival under hypoxia, whereas p53 acts as a context-dependent regulator of cell fate depending on stress severity and cellular adaptability. This context-dependent role of p53 is further supported by previous findings demonstrating that p53 can also contribute to cell survival under hypoxic conditions. Feng et al. (2011) showed that p53-mediated repression of the HIF-1 target gene BNIP3 protects HCT116 cells from hypoxia-induced cell death. This highlights that p53 is not exclusively pro-apoptotic, but can also limit cell death by selectively modulating HIF-1 target gene expression, thereby contributing to cellular adaptation in a stress-dependent manner. Together, these observations support a model in which p53 and HIF-1α function within an integrated regulatory network balancing hypoxic adaptation and apoptosis, rather than acting as strictly opposing determinants of cell survival. To understand the impact of p53 on metabolic adaptation under hypoxia, we examined its influence on the stability and activity of HIF-1, the central transcription factor that mediates the cellular response to hypoxia. Interestingly, while p53 deficiency had no effect on HIF-1α protein levels (Fig. 3B), it significantly impaired the early induction of canonical HIF-1 driven target genes for angiogenesis and oxygen sensing (*VEGF*, *PHD2*) after 4 h of hypoxia (Fig. 3C). Importantly, this lack of effect on HIF-1α protein levels indicates that p53 does not regulate HIF-1α abundance under these conditions. Nevertheless, in addition to *VEGF* and *PHD2*, we also observed reduced expression of further established HIF-1 target genes involved in glycolytic metabolism, including *GLUT1*, *PFKL*, and *LDHA*. These genes represent canonical markers of HIF-1 transcriptional output, and their coordinated downregulation suggests that p53 loss affects HIF-1 functional activity rather than its protein stability or abundance. However, this effect appears to be context-dependent and most prominent at early hypoxic time points.

We acknowledge that two time points cannot fully capture the dynamic continuum of hypoxic adaptation. Our classification of “early” and “prolonged” hypoxia refers specifically to the 4 h and 24 h time points studied here. Concerning the influence of p53 on the transcriptional activity of HIF-1, we disprove the hypothesis of Ravi et al. [15] that p53 ko cells exhibit increased *VEGF* mRNA expression. Instead, we strengthen the observation of Farhang Ghahrameni et al. [16] who reported that p53 binding to the *VEGF* promoter under hypoxia is important for the activation of *VEGF* mRNA expression. This transcriptional defect in p53 ko cells occurred despite normal HIF-1α DNA-binding capacity (Fig. 3E) and ARNT availability (Fig. 3F). Thus, we could not find any evidence for the observation of Madan et al. [17] that p53 enhances the binding of HIF-1 to the HRE. These findings suggest that p53 may regulate HIF-1 transcriptional output downstream of DNA binding, potentially through effects on co-activator recruitment, chromatin accessibility, or promoter-specific transcriptional competence [28, 29]. However, the precise mechanism underlying this selective impairment remains unresolved and warrants further investigation.

To place these findings into a broader metabolic context, it is important to consider the well-established role of p53 in regulating cellular metabolism under normoxic conditions. Under normoxia, p53 is known to promote mitochondrial respiration and repress glycolysis through several mechanisms, including downregulation of key regulators of glucose uptake such as *GLUT1* and *GLUT4*, as shown by Schwartzenberg-Bar-Yoseph et al. [6] in osteosarcoma- and rhabdomyosarcoma-derived cells. In our study, p53 ko cells presented lower expression of *GLUT1* as well as *PFKL* under normoxia than p53 wt cells did (Fig. 4). Hence, our results indicate that the influence of p53 on glycolytic gene expression under normoxia may be cell type specific. The basal expression of *GLUT1* under normoxic conditions is regulated by several transcription factors, including Sp1 [30, 31] and c-Myc [32]. In addition, signalling pathways that modulate HIF-1, Sp1, and c-Myc, most notably the PI3K/AKT/mTOR pathway, play a central role in shaping the metabolic phenotype by controlling the expression of glycolytic genes as well as the translocation of GLUT transporters to the plasma membrane [33, 34].

Given the highly interconnected nature of cellular signalling networks, the loss of a single signalling component can result in diverse, context-dependent effects. For instance, p53 induces PTEN (phosphatase and tensin homolog) [35], a key negative regulator of the PI3K pathway [36]. Inhibition of PI3K signalling leads to reduced AKT phosphorylation and consequently diminished activation of mTOR [37]. Moreover, p53 can suppress mTOR activity through additional mechanisms, including activation of AMPK and its downstream target TSC2 [38], as well as induction of REDD1 [39].

Consistent with these regulatory roles, loss of p53 is generally associated with increased AKT and mTOR activity. Both the PI3K pathway and mTOR have been shown to promote c-Myc activity, a central regulator of glycolysis that drives the expression of numerous glycolytic genes [40]. Based on this, one might expect an upregulation of glycolytic target genes in p53-deficient cells.

However, mTOR activation also triggers phosphorylation of S6K, which in turn inhibits PI3K signalling via IRS1, establishing a negative feedback loop [41]. Consequently, hyperactivation of the PI3K pathway following p53 loss may paradoxically lead to feedback-mediated attenuation of PI3K signalling and its downstream effectors. Taken together, these complex regulatory interactions make it difficult to predict the net impact of p53 loss on glycolytic gene expression, as the strength and dynamics of compensatory feedback mechanisms are likely to vary in a cell type–specific manner. Our findings challenge the established view of p53 as a transcription factor closely exclusively supporting mitochondrial respiration.

Despite decreased *GLUT1* mRNA expression levels compared to those in p53 wt cells, p53 ko cells demonstrated higher glycolysis rates under normoxia (Fig. 5A), also known as the Warburg effect, reinforcing the importance of posttranscriptional mechanisms for glycolytic flux.

Higher basal glycolysis in p53 knockout cells under normoxia can be explained by the broader metabolic control exerted by p53 beyond transcriptional regulation of GLUT1 and PFKL alone [42, 43]. Wild-type p53 represses multiple nodes of the glycolytic program and promotes mitochondrial respiration. Consequently, loss of p53 shifts cellular metabolism toward increased aerobic glycolysis. In addition to TIGAR-mediated control of fructose-2,6-bisphosphate levels and PFK1 activity, p53 also restrains glycolysis through regulators such as PFKFB3 and through inhibition of alternative glucose uptake pathways [43, 44]. Moreover, p53 loss can unleash pro-glycolytic signaling programs, including NF-κB–dependent metabolic regulation, and enhance glucose uptake via compensatory transporters or increased transporter trafficking [45]. As a result, glycolytic flux can be substantially increased in p53-deficient cells even in the absence of marked transcriptional upregulation of GLUT1 or PFKL. Thus, the elevated basal glycolysis observed under normoxia reflects metabolic rewiring and post-translational control rather than direct correspondence with individual gene expression levels.

To date, the regulation of glycolytic gene expression by p53 has been primarily examined under normoxia. The implications of p53 loss for metabolic adaptation to hypoxia were unclear. We are the first to reveal an altered temporal pattern of glycolytic gene expression due to a p53 knockout under hypoxia, which further highlights the dynamic role of p53 in the regulation of metabolism (Fig. 4). After four hours of hypoxia, p53-deficient cells exhibited an attenuated transcriptional response concerning glycolytic target genes of HIF-1, which matched our observations regarding other HIF-1 targets responsible for angiogenesis and oxygen sensing (*PHD2*, *VEGF*) in p53 ko cells. However, after 24 h under hypoxia, p53 ko cells exhibited significant upregulation of the HIF-1 target genes *LDHA* and *GLUT1*, which are related to glycolytic flux, glucose uptake, NAD⁺ regeneration and lactate production central to anaerobic metabolism. The delayed upregulation of glycolytic target genes suggests that compensatory pathways are engaged during prolonged hypoxia in p53-deficient cells, likely as a consequence of impaired HIF-1–dependent transcription. Consistent with this, Toshiro Kawauchi et al. did not observe an increase in *GLUT1* mRNA expression in p53 ko MEF cells and instead proposed a model in which loss of p53 promotes IKK-mediated activation of NF-κB, thereby enhancing glycolysis [46]. The importance of IKK for the induction of HIF-1 target genes under hypoxic conditions was previously highlighted by Josep Rius et al., further supporting a link between inflammatory signalling and metabolic adaptation [47].

Moreover, Katsumi Nakayama and colleagues demonstrated that full activation of NF-κB under hypoxia is delayed, becoming prominent only after 24 h and reaching maximal levels at later time points [48]. In conclusion, the observed temporal shift suggests that NF-κB may compensate for declining HIF-1 activity during prolonged hypoxia. In addition, p53 and NF-κB compete for the transcriptional coactivator CREB-binding protein (CBP); thus, loss of p53 may facilitate increased NF-κB access to CBP, further enhancing NF-κB–dependent transcriptional programs [49].

Nevertheless, it is striking that *GLUT1* mRNA expression is significantly reduced after 24 h under hypoxia in p53 ko cells compared to p53 wt cells whereas LDHA expression is significantly increased. Notably, this differential regulation of individual glycolytic genes under hypoxia does not indicate a functional inconsistency (Fig. 5).

At first glance, the divergent regulation of *LDHA* and *GLUT1* at 24 h of hypoxia appears contradictory because LDHA-driven lactate production requires sustained glycolytic flux. However, this pattern can be reconciled mechanistically. The apparent dissociation between LDHA induction and the expression of upstream glycolytic regulators under hypoxia does not represent a functional contradiction but rather reflects hierarchical and multi-layered metabolic control. LDHA is a strongly hypoxia-inducible target of HIF signalling, whose transcriptional activation can occur even when glucose uptake and early glycolytic steps are not proportionally upregulated [50, 51]. In parallel, hypoxia can stabilize p53 and enhance its transrepressive functions, including repression of glycolytic genes and activation of targets such as RRAD, which has been shown to inhibit GLUT1 trafficking to the plasma membrane [52]. Under these conditions, LDHA expression may increase while effective glucose uptake remains constrained at the level of GLUT1 transcription and/or surface availability, such that LDHA induction does not automatically translate into a marked increase in net glycolytic flux. Instead, elevated LDHA may primarily serve to prevent pyruvate accumulation and to support NAD⁺ regeneration for the available glycolytic throughput, rather than driving high-capacity lactate export.

In this context, the limited transcriptional response of PFKL in p53 knockout cells after 24 h of hypoxia is also mechanistically plausible. Although PFKL is a HIF-1 target, its induction is strongly context dependent and influenced by additional regulatory inputs, including HIF cofactors and metabolic feedback mechanisms [27]. Moreover, PFK1 activity is predominantly controlled post-translationally through allosteric regulation by ATP, AMP, citrate, and especially fructose-2,6-bisphosphate, as well as through p53-dependent pathways involving TIGAR [53, 54] (explained in the further discussion). Consequently, modest or absent changes in PFKL mRNA do not preclude substantial modulation of glycolytic flux. Together, these mechanisms explain why LDHA can function as a relatively direct hypoxic readout, while GLUT1 and PFKL represent regulatory nodes where hypoxia, p53 signalling, and post-transcriptional control intersect, allowing their expression and functional impact on glycolysis to diverge from LDHA despite the biochemical requirement of LDHA for sustained glycolytic metabolism.

Our data suggest, but do not formally prove, that p53 contributes to the early activation of HIF-1 target genes. Definitive proof will require rescue or mutant re-expression approaches, which are an important goal for future work. Further studies will be required to determine whether the temporal pattern observed here is conserved across other colorectal cancer models, including cells with p53 gain-of-function mutants such as R175H and R273H. Another explanatory approach for the observation that p53 ko cells were able to perform similar glycolysis rates as p53 wt cells despite reduced gene expression might be derived from the work of Bensaad et al. [5], who investigated the allosteric effects of the p53 target gene *TIGAR* on glycolytic genes. TIGAR functions as a bisphosphatase and can downregulate glycolysis via the degradation of fructose-2,6-bisphosphate, an essential stimulator of the phosphofructokinase (PFKL), the key enzyme of aerobic glycolysis. In p53 ko cells, *TIGAR* expression is possibly reduced, enabling increased activity of PFKL. The importance of allosteric effects becomes clearer when considering that p53 ko cells have the same glycolytic capacity as p53 wt cells under normoxia (Fig. 5A).

Surprisingly, the loss of *HIF1A* did not impair glycolytic function under hypoxia (Fig. 7), even though all the tested HIF-1 target genes, including genes encoding essential glycolytic enzymes, were markedly repressed (Fig. 6). This observation, which was also made in K562 cells [55], reveals a potential HIF-1-independent mechanism supporting glycolysis under hypoxia, which may include the activation of alternative transcription factors such as P53 and MYC [56] or the posttranscriptional regulation of glycolytic enzymes. It also emphasizes that transcriptional upregulation of metabolic genes is not always required to sustain their activity under stress. For example, Valli et al. [57] performed metabolic flux analysis in spheroids deriving from HCT116 HIF1A ko cells with a hypoxic inner mass. Their metabolic flux analysis showed that despite lack of HIF-1α, HCT116 cells were able to maintain glycolytic flux possibly by HIF-2 dependent upregulation of *GLUT14*, another glucose uptake transporter. In our study, the protein stabilization of the HIF-2α-subunit was also significantly increased in HCT116 HIF1A ko cells compared to HCT116 HIF1A wt cells strengthening the observation of Valli et al. and bringing another HIF isoform into the play for metabolic adaptation to hypoxia (Supplemental Fig. 1). Besides, the enzyme normally responsible for the speed-determining step of glycolysis, the PFKL, did not seem to limit the glycolytic process [57]. A reason for this observation could be delivered by Golinska et al. who were also not able to detect a reduced glucose uptake in ANRT-deficient hepatomas. Their study points out that neither HIF-1 nor HIF-2 are necessary for the increase in glycolytic flux under hypoxia. Instead, they suggest stronger activation of PFK as a result of higher accumulation of AMP in the absence of HIF [58]. Our study complements well with the studies mentioned above as we are the first using extracellular flux analysis to show that these changes do not impact glycolytic rate and particularly compensatory glycolytic rate of a single layer of colorectal carcinoma cells under moderate hypoxia (1% O_2_) whereas Valli et al. conducted metabolic flux analysis in HCT116 HIF1A ko and wt spheroids with a hypoxic inner mass. Nevertheless, further metabolic flux, ATP production and isotope tracing experiments will be necessary to examine compensatory pathways and mechanisms for preserved glycolysis under hypoxia despite lack of HIF-1α.Although the glycolysis rates of HIF1A ko cells were similar to those of HIF1A wt cells, their glucose consumption and lactate production were significantly greater (Fig. 7C), possibly due to enhanced proliferation. Our observations support the hypothesis of Gordan et al. [59] that HIF-1α inhibits cell cycle progression in HCT116 cells by suppressing the protooncogene c-Myc. On the other hand, c-Myc can promote p53 protein stabilization through p14ARF activation [60]. These molecular mechanisms could explain why a *HIF1A* loss not only accelerates cell cycle progression but also enhances p53 activation and *BAX* expression, leading to increased apoptotic cell death rates (Fig. 8). HIF-1 and p53 are integrated in this complex network of protooncogenes and tumour suppressors, balancing survival and death under extreme oxygen and nutrient limitations. While HIF-1 promotes adaptation to hypoxia, p53 appears to limit survival under irrecoverable stress.

In conclusion, our findings redefine p53 not simply as an antagonist of HIF-1 or a passive responder to hypoxia but also as a temporal gatekeeper of hypoxic adaptation. p53 enables early HIF-1-driven gene induction, modulates metabolic responses, and promotes apoptosis under persistent stress, highlighting its integrative, stage-specific role in the hypoxic stress response. We demonstrate for the first time that p53 is required for timely activation but not maintenance, of hypoxia-responsive gene expression.

Moreover, colorectal cancer cells can compensate for the loss of both p53 and HIF-1α to preserve glycolysis, indicating the existence of HIF-independent survival circuits under hypoxia. These results suggest that p53-deficient tumour cells may be initially metabolically vulnerable under hypoxia but can evolve alternative adaptation strategies over time. Therefore, therapeutic targeting of these compensatory pathways, rather than single transcriptional regulators, could offer more effective strategies against therapy-resistant, hypoxic, and p53-mutant solid tumours.

**Fig. 9:**
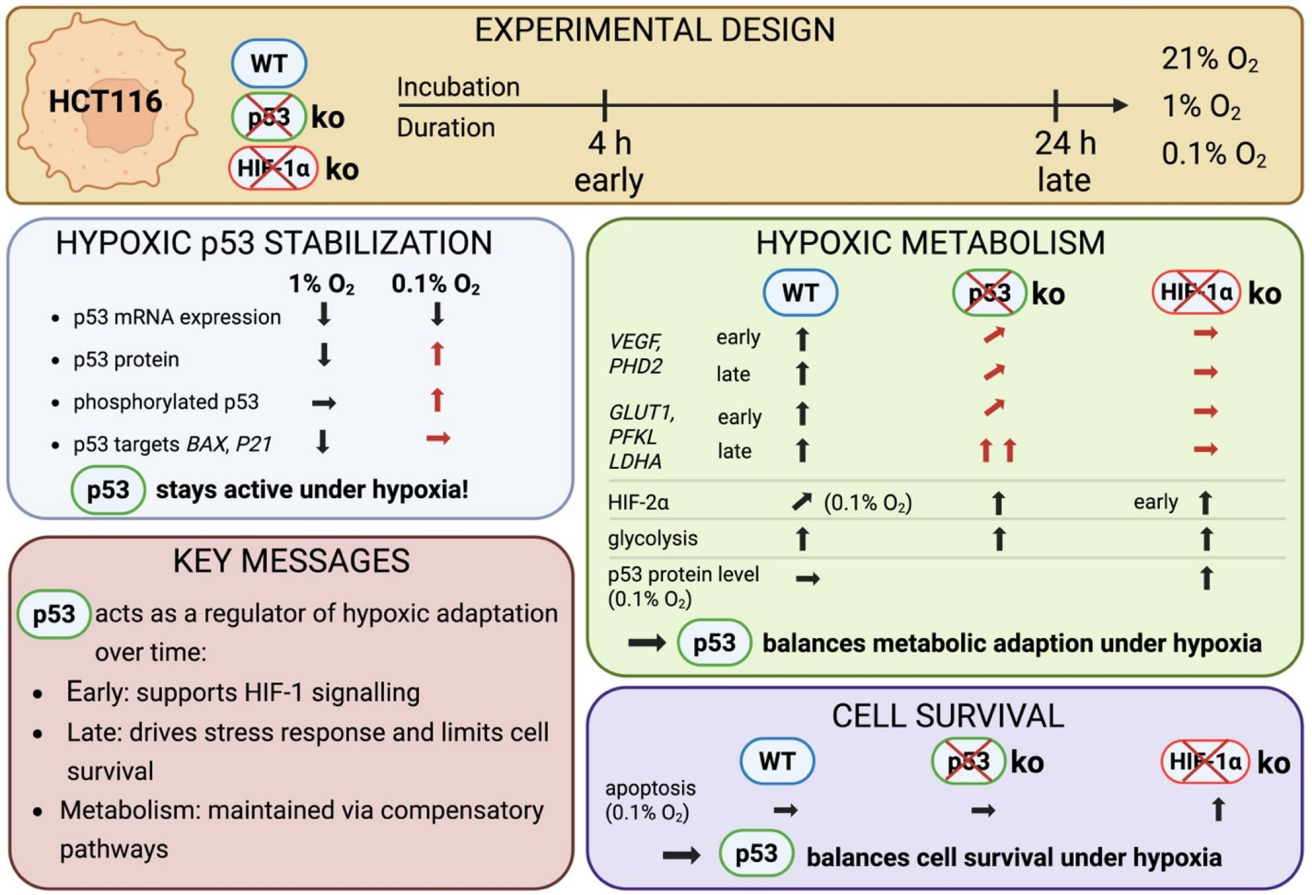
Schematic summary of p53-dependent and -independent regulation of hypoxic adaptation in HCT116 cells. Schematic overview of the experimental design and key findings. HCT116 wild-type (WT), p53 knockout (p53 KO), and HIF1A knockout (HIF-1α KO) cells were analysed under normoxia (21% O₂), moderate hypoxia (1% O₂), and severe hypoxia (0.1% O₂) for or early (4 h) and late (24 h) time points. Under hypoxia, p53 protein levels and phosphorylation increase despite reduced mRNA expression, leading to sustained activation of target genes (e.g., BAX, P21). Metabolic analysis shows induction of hypoxia-responsive genes (VEGF, PHD2) and glycolytic enzymes (GLUT1, PFKL, LDHA), with p53 modulating the magnitude and timing of these responses. HIF-2α contributes particularly under severe hypoxia (0.1% O₂). Functionally, p53 supports early hypoxic signaling, promotes stress responses at later stages, and balances metabolic adaptation and cell survival. Overall, p53 acts as a key regulator of hypoxic adaptation by coordinating transcriptional activity, metabolism, and apoptosis. Created in https://BioRender.com

Figure 9 shows the schematic summary of p53-dependent and -independent regulation of hypoxic adaptation in colorectal cancer cells.

## Supplementary Information

Supplementary Material 1.

## Data Availability

All data supporting the findings of this study are available within the paper.
