# Supplementary material for: P53 – a new player in the metabolic adaptation of colorectal carcinoma cells under hypoxia
Source: BMC Cancer. 2026 May 19;26:628. doi: 10.1186/s12885-026-16184-y (PMC13185365; doi:10.1186/s12885-026-16184-y)
Supplement: Supplementary file 1 — Supplementary Material 1. [file 12885_2026_16184_MOESM1_ESM.docx]

**Supplemental figures**

**Supplemental figure 1
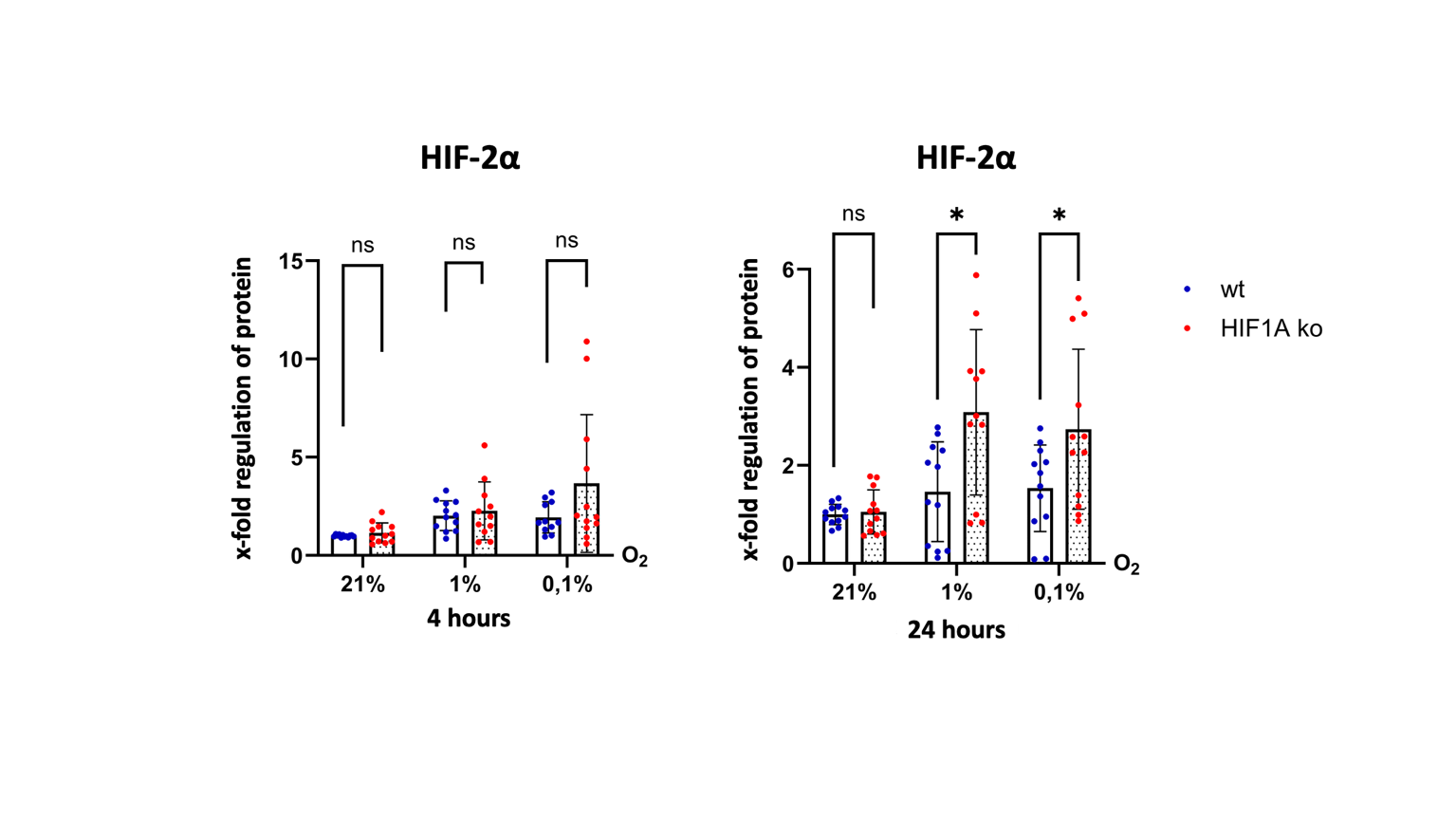
**

**Supplemental figure 1: Increased HIF-2α protein in HIF1A ko HCT116 cells.** Western Blot analysis of HIF-2 protein expression after four and 24 hours under normoxia (21% O2), moderate (1% O2) and severe (0.1% O2) hypoxia.

**Supplemental figure 2**

**
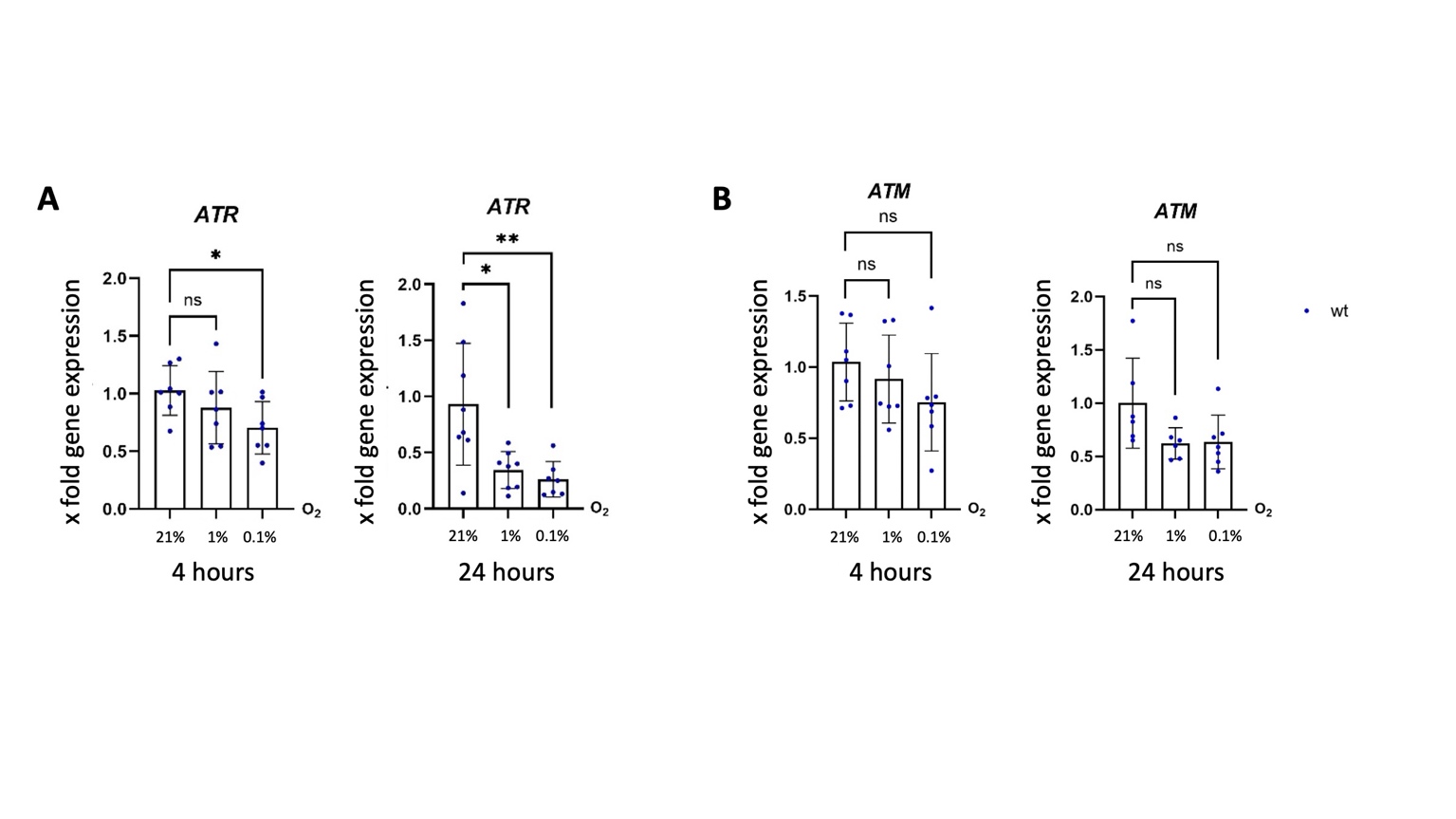
**

**Supplemental figure 2: Hypoxia-dependent downregulation of *ATR* expression but not altered *ATM* expression in HCT116 wild-type cells.** Expression of ATR (A) and ATM (B) mRNA (A) in HCT116 wild-type cells after 4 and 24 hours under normoxia (21% O₂), moderate hypoxia (1% O₂), and severe hypoxia (0.1% O₂). Quantitative PCR analysis revealed a significant downregulation of ATR mRNA expression after 4 hours of severe and 24 hours of hypoxic incubation (n = 11), whereas ATM mRNA levels remained unchanged under all conditions.

**Supplemental figure 3**

**
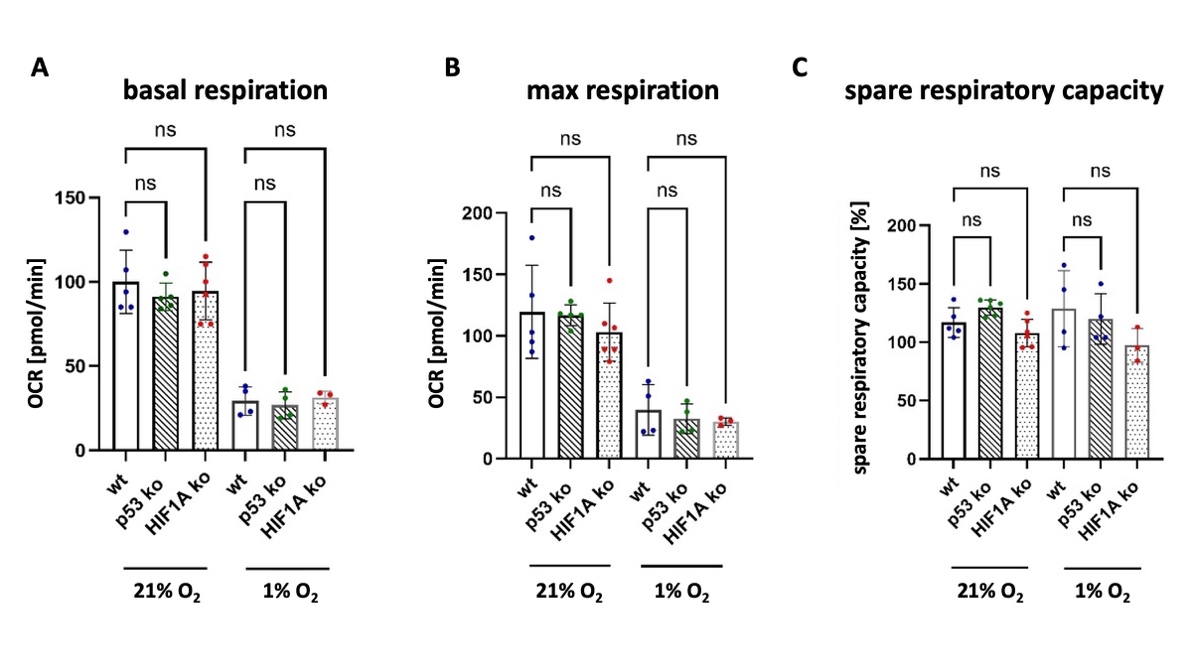
**

**Supplemental figure 3: Loss of p53 or HIF1A does not affect mitochondrial respiration in HCT116 cells under normoxia or hypoxia.** We compared mitochondrial stress test results for HCT116 wild-type (WT), p53 knockout (KO), and HIF1A knockout (KO) cells under normoxia (21% O₂) and moderate hypoxia (1% O₂). Neither the p53 knockout nor the HIF1A knockout alters the basal (A) or maximal (B) respiratory rate under normoxia or hypoxia. Therefore, the respiratory capacity (C) of the knockout cell lines does not differ from that of the wild-type cell line. WT and p53 KO: n = 5 under normoxia and n = 4 under hypoxia. HIF1A KO: n = 6 under normoxia and n = 3 under hypoxia.

**Supplemental figure 4**

**
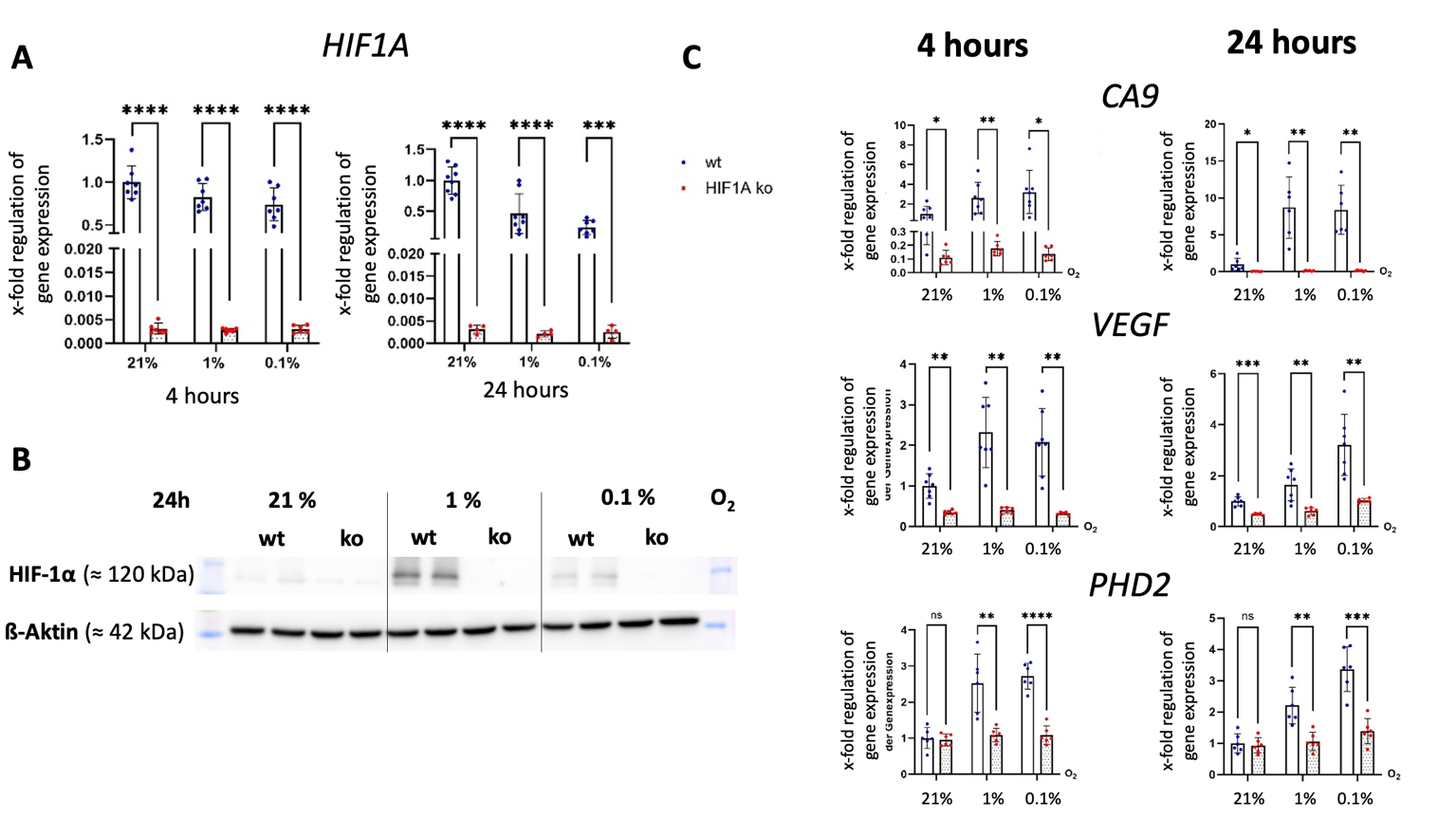
**

**Supplemental figure 4: *HIF1A* ko validation.** (A) qPCR of showed a strong downregulation of *HIF1A* gene expression after CRIPR-Cas9 knockout. (B) Western blot analysis revealed a clear knockout of HIF-1α under the applied conditions. Example picture of a Western blot. (C) Target genes of HIF-1α were highly downregulated after knockout procedure under all applied conditions

**Supplemental Western blot single pictures**

# Western Blots for Figure 1 B

V1 wt 4 hours


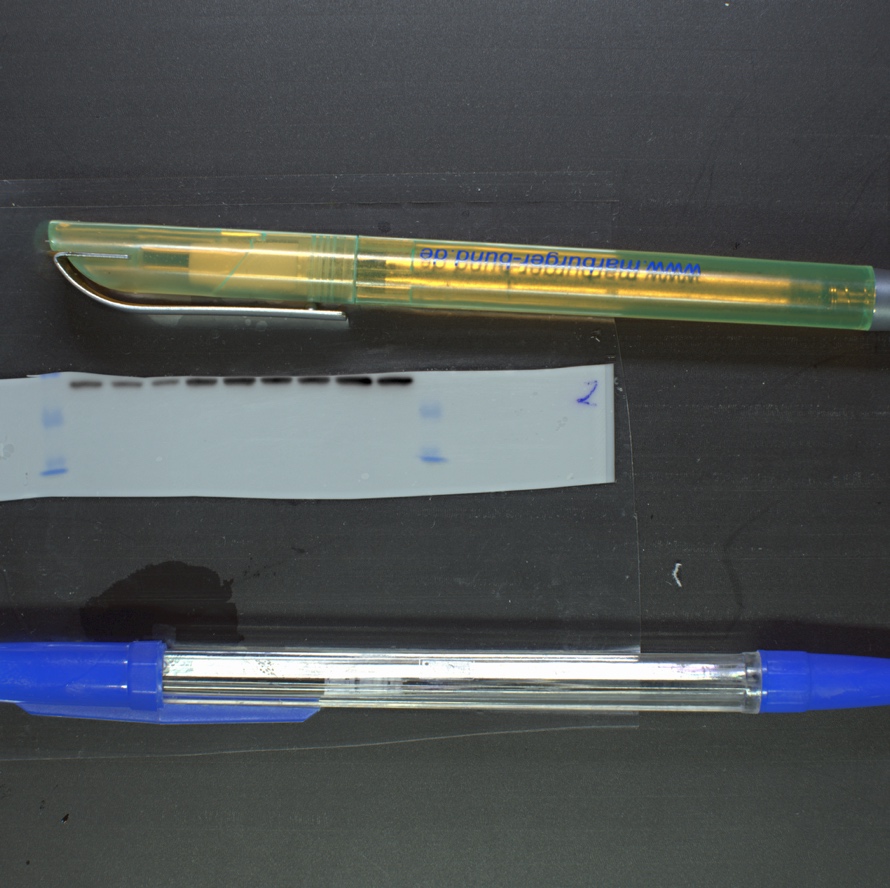

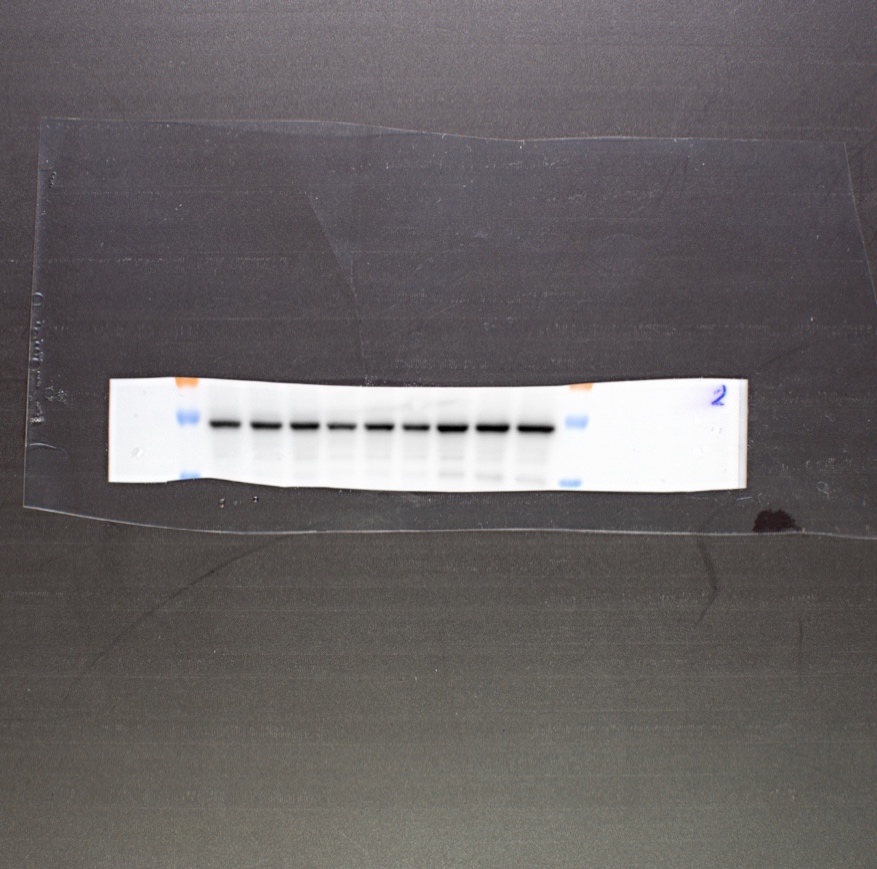

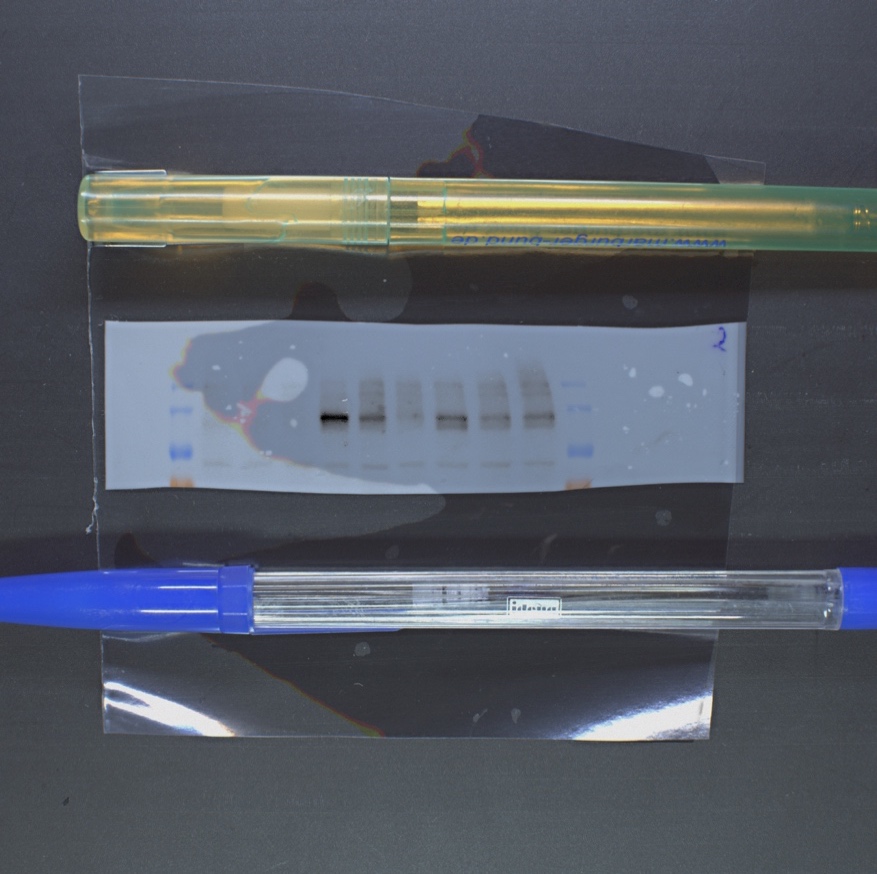

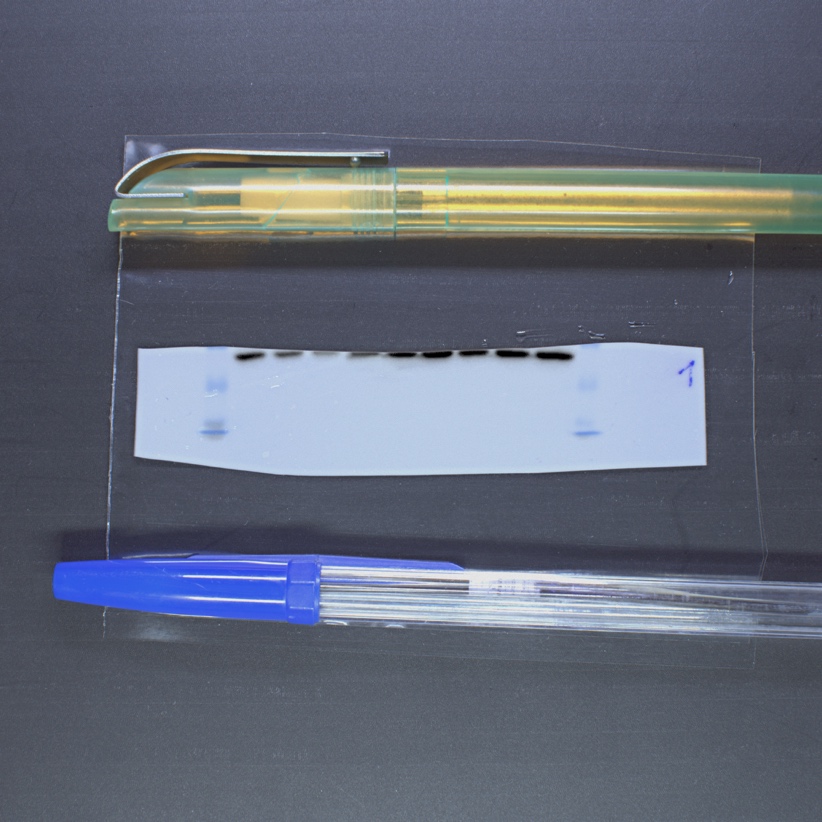

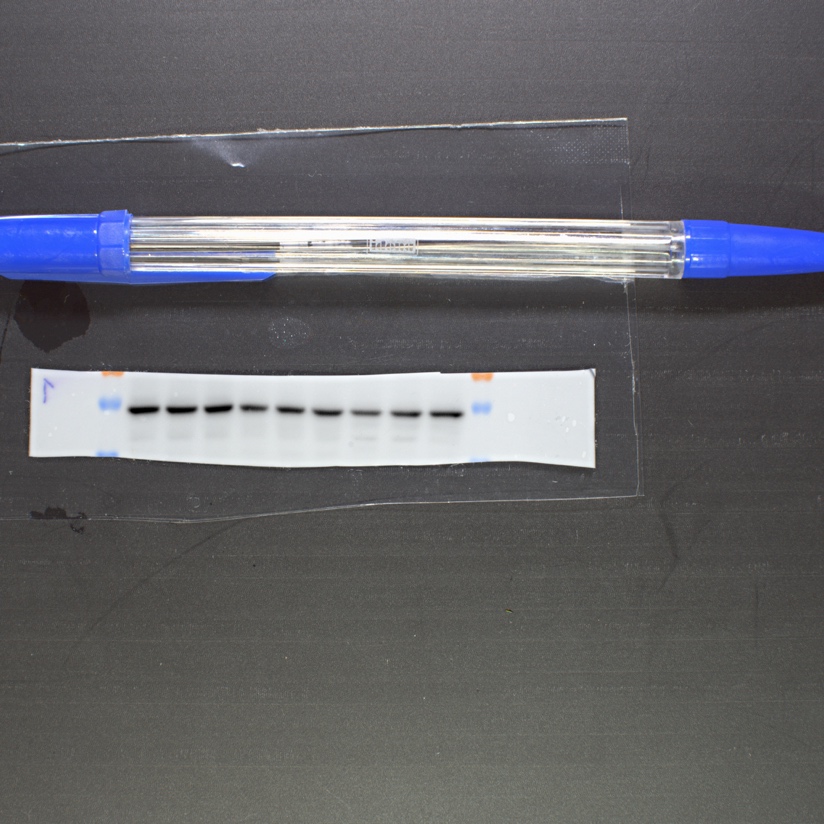

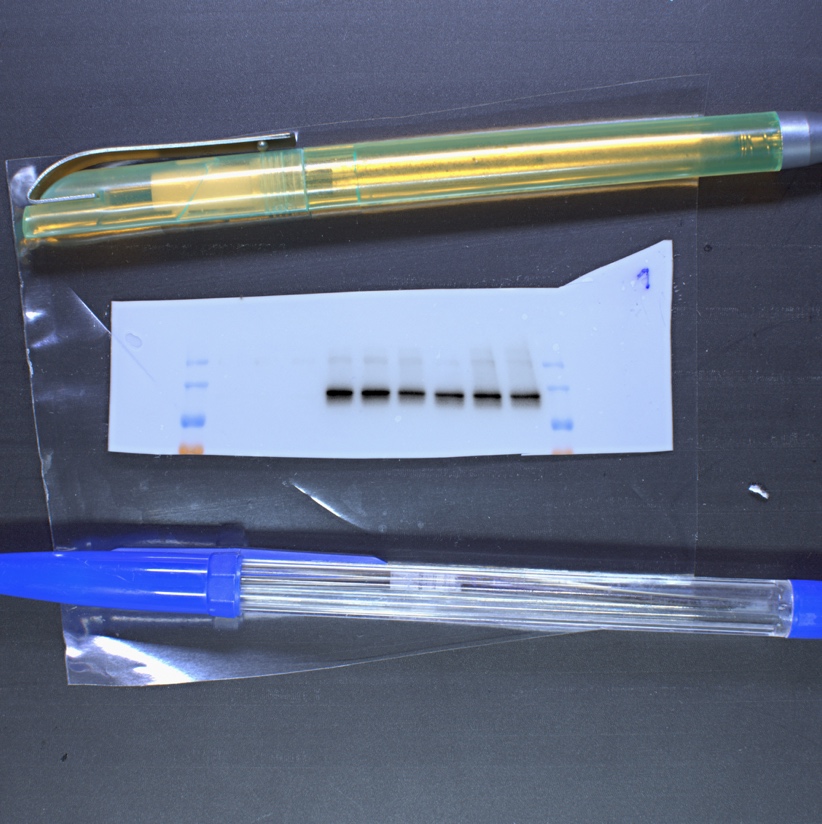


V1 wt 24 hours

HIF1A

P53

RPLP01

RPLP01

P53

HIF1A

V2 wt 4 hours


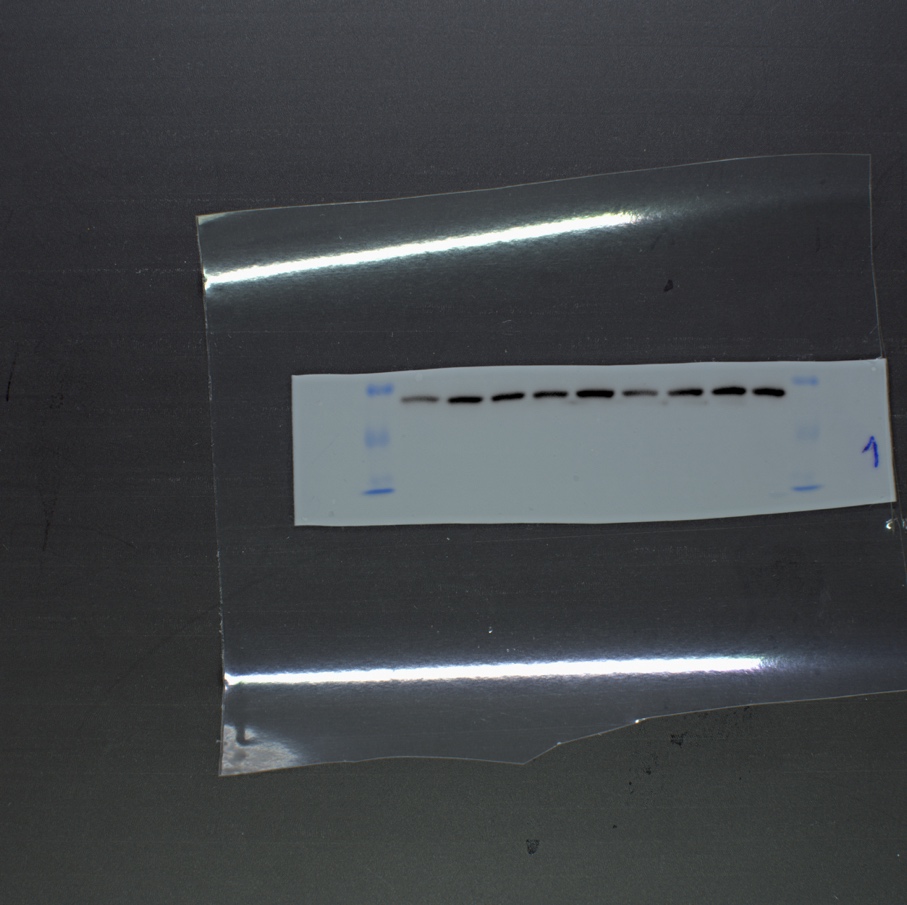

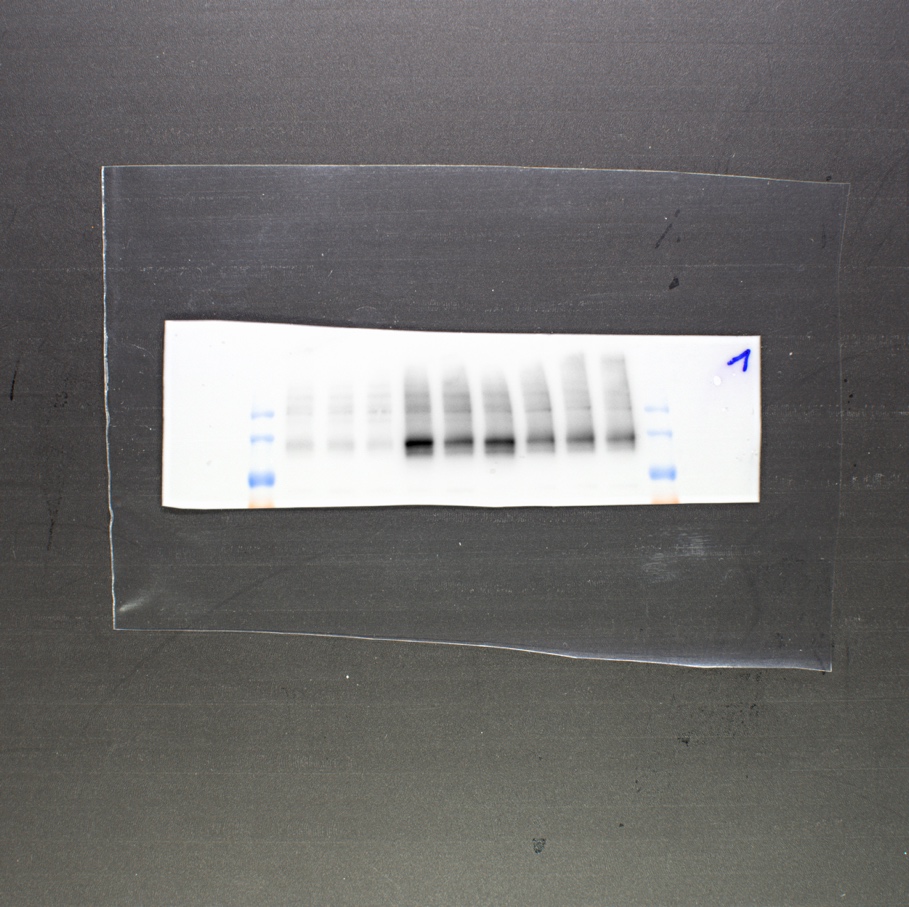

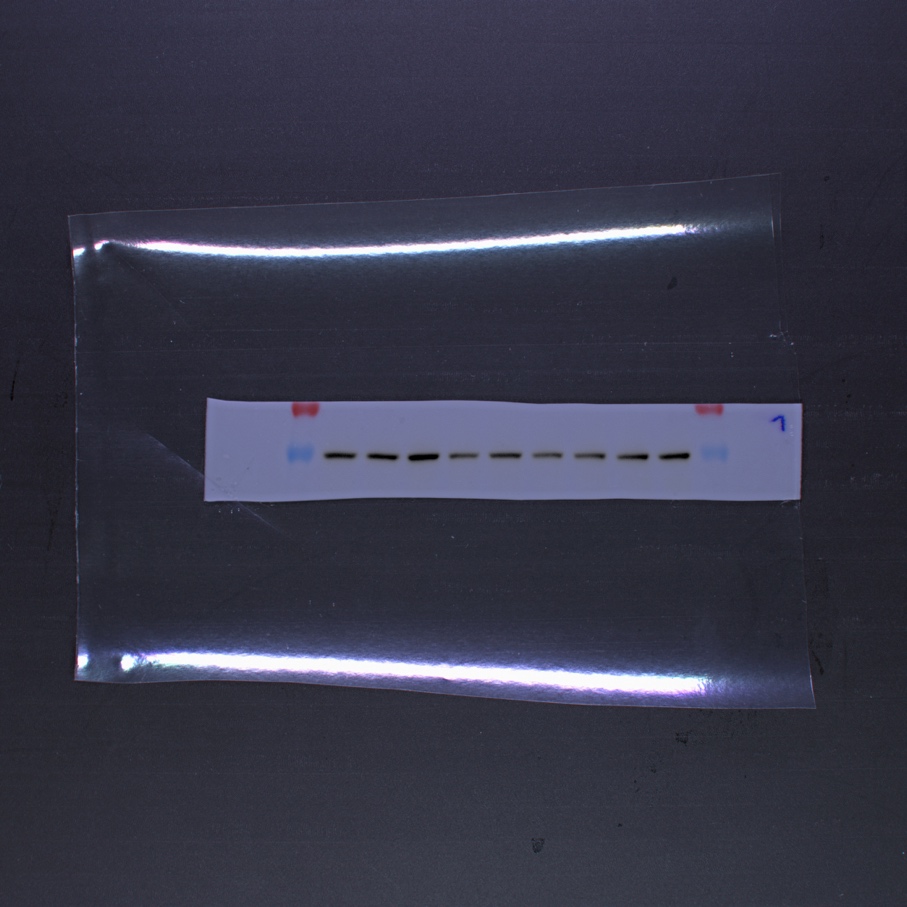


HIF1A

RPLP01

P53

**
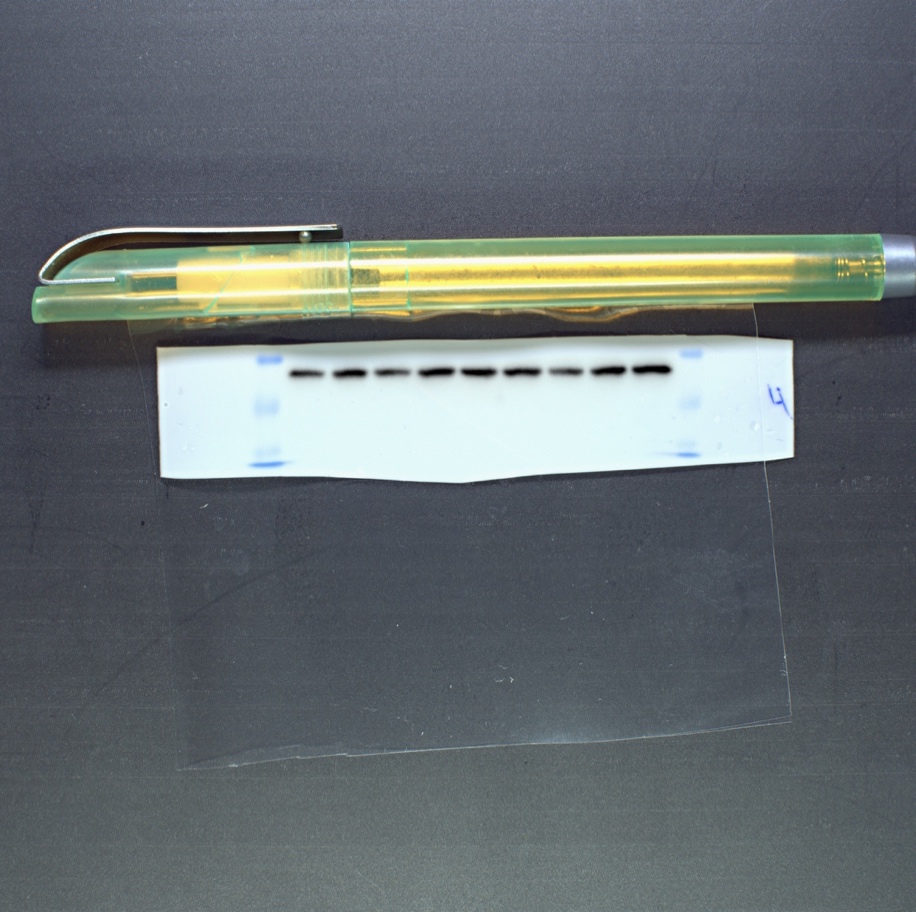

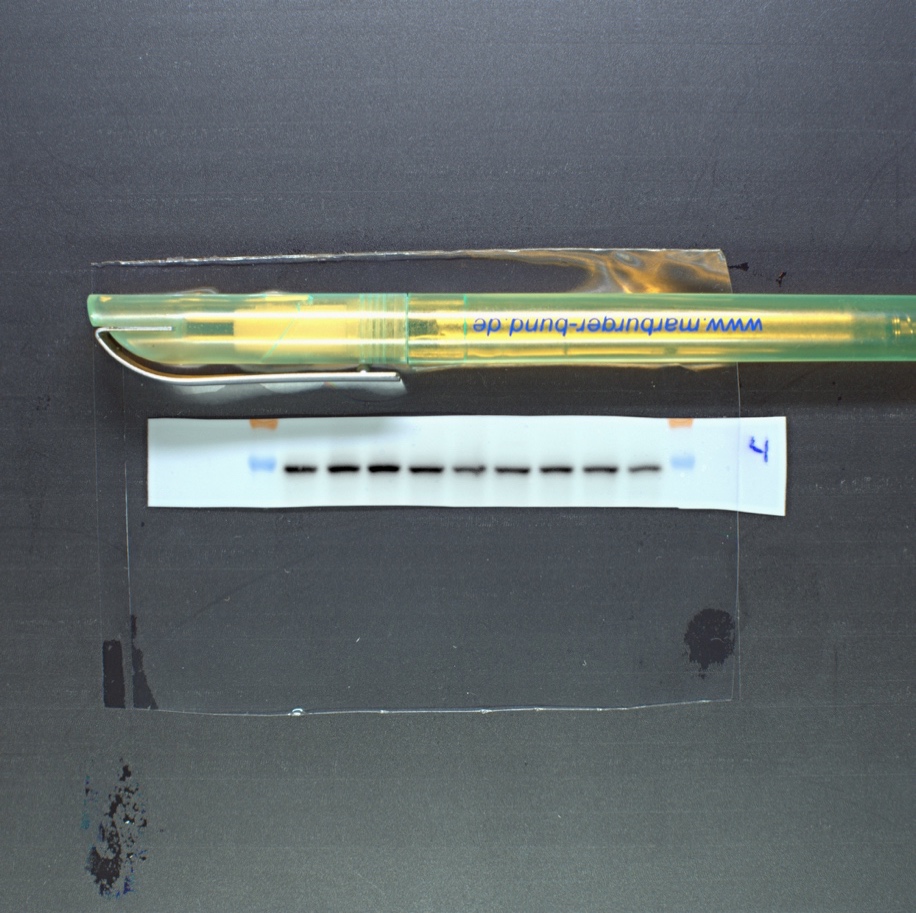

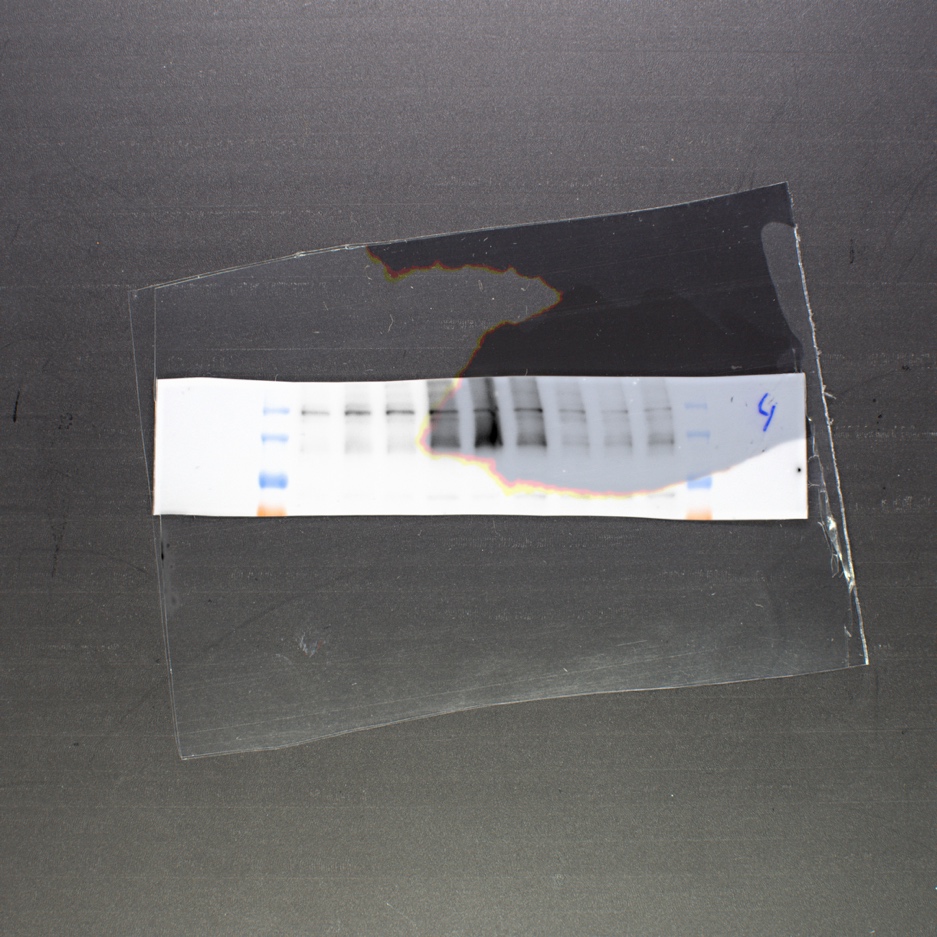

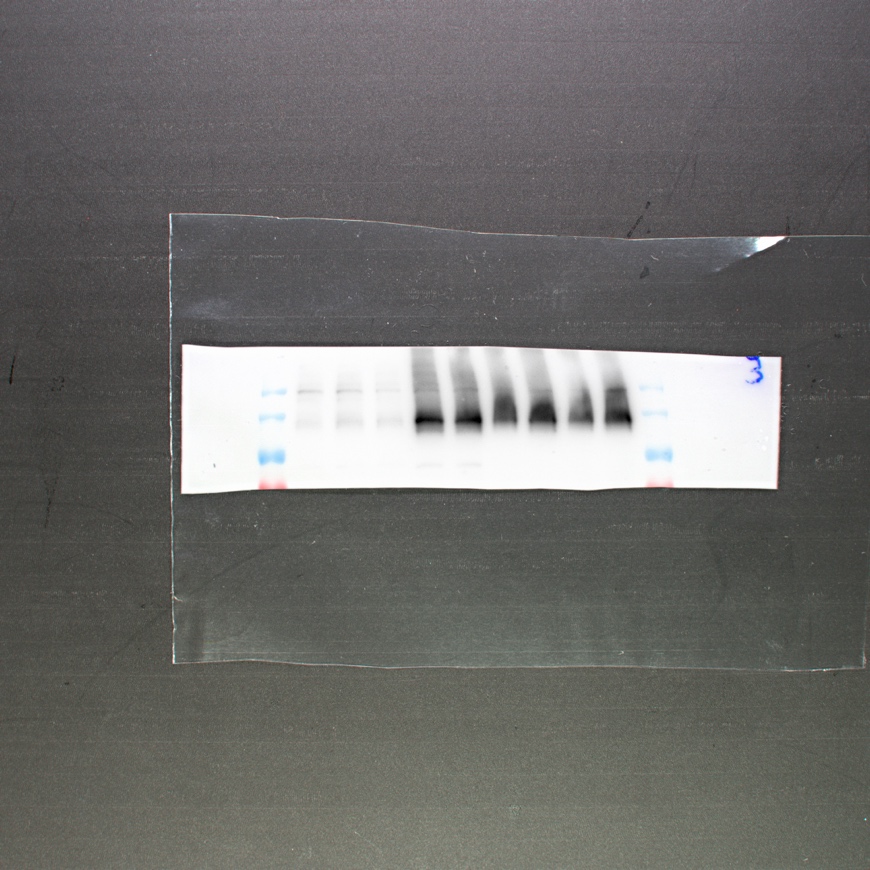

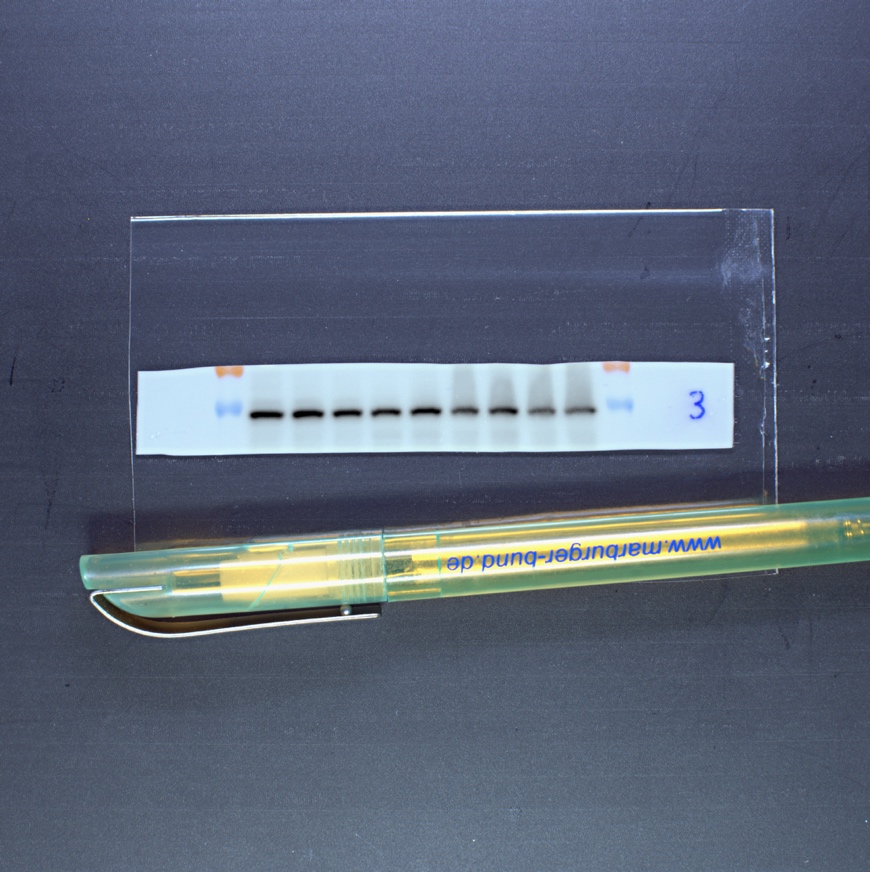

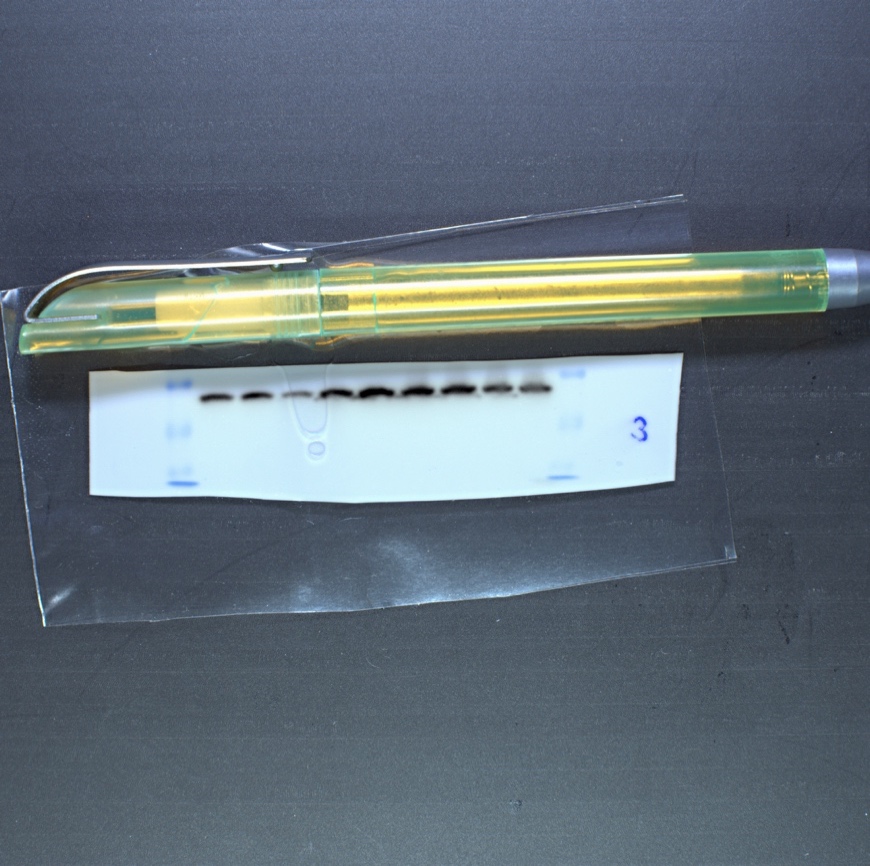

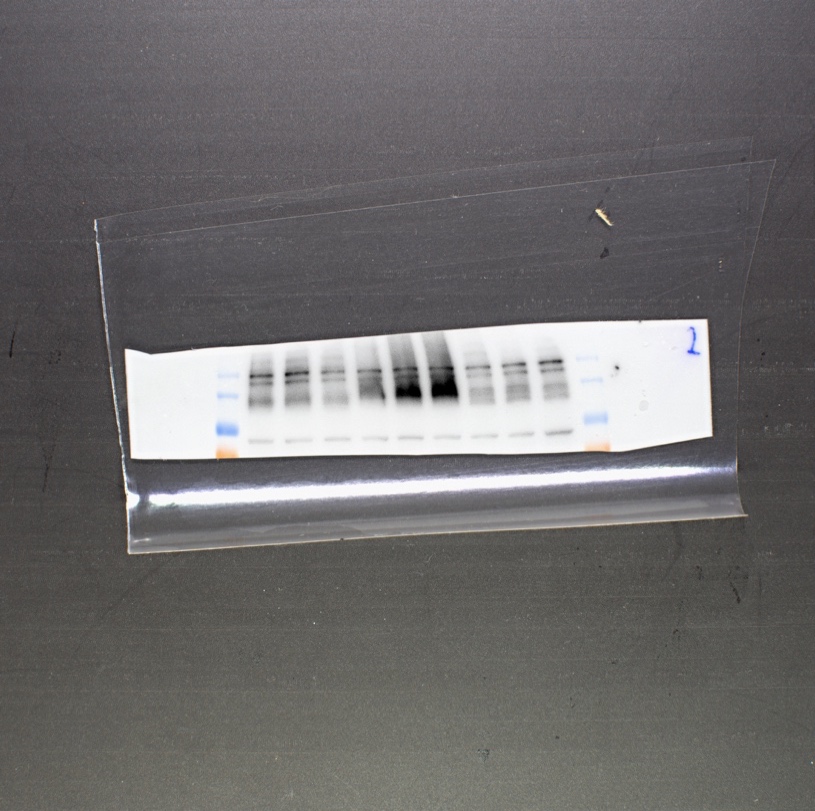

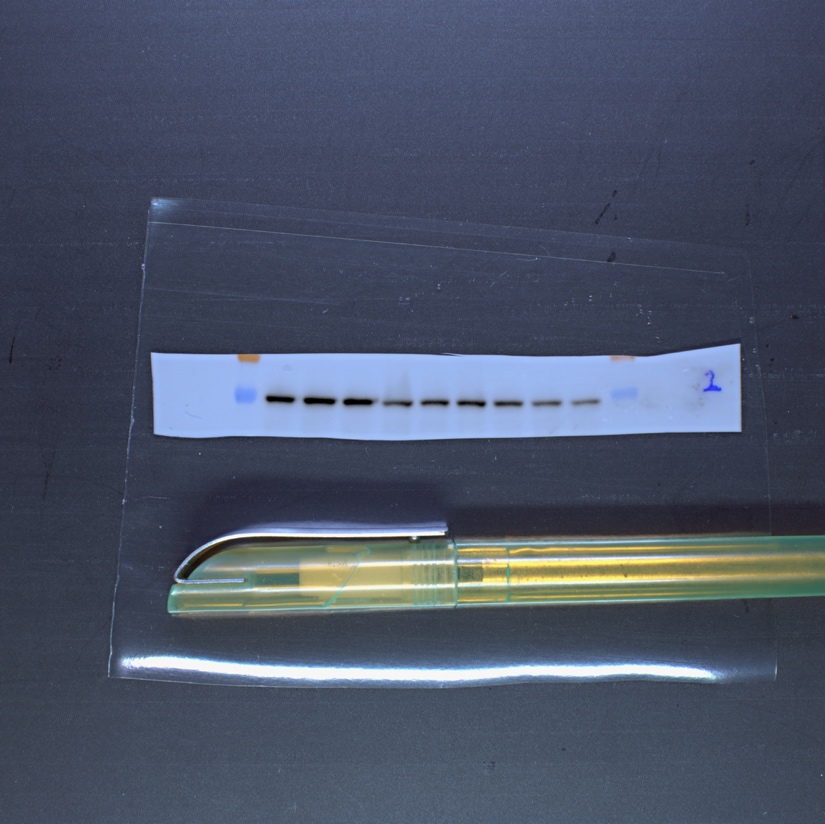

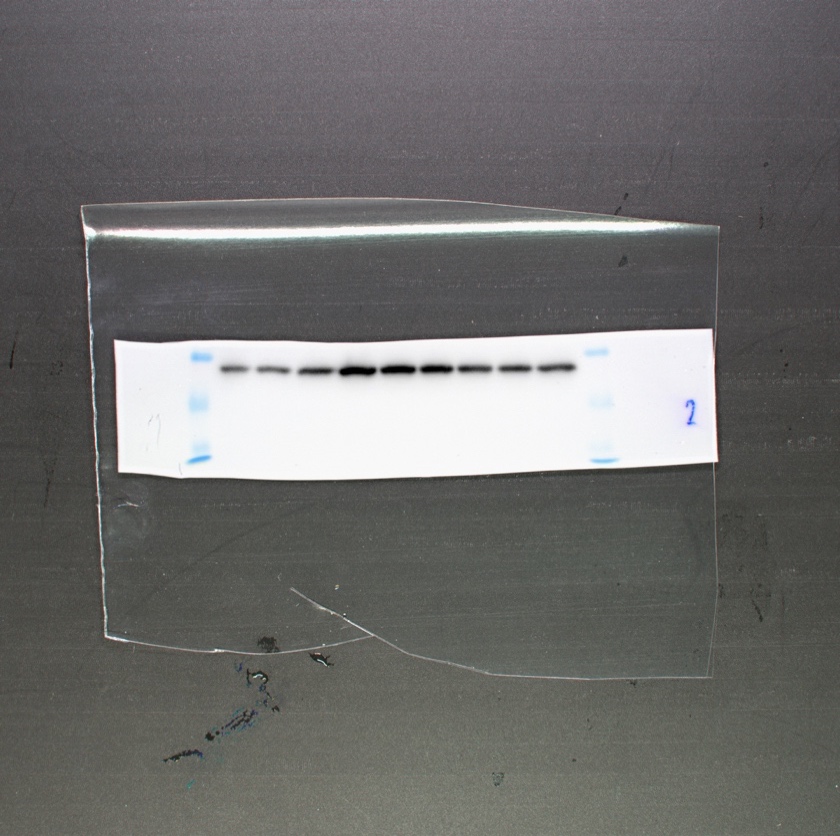
**

RPLP01

P53

HIF1A

RPLP01

P53

HIF1A

RPLP01

P53

HIF1A

HIF1A

P53

RPLP01

V2 wt 24 hours

V3 wt 4 hours

V3 wt 24 hours

V 4 wt 4 hours

**
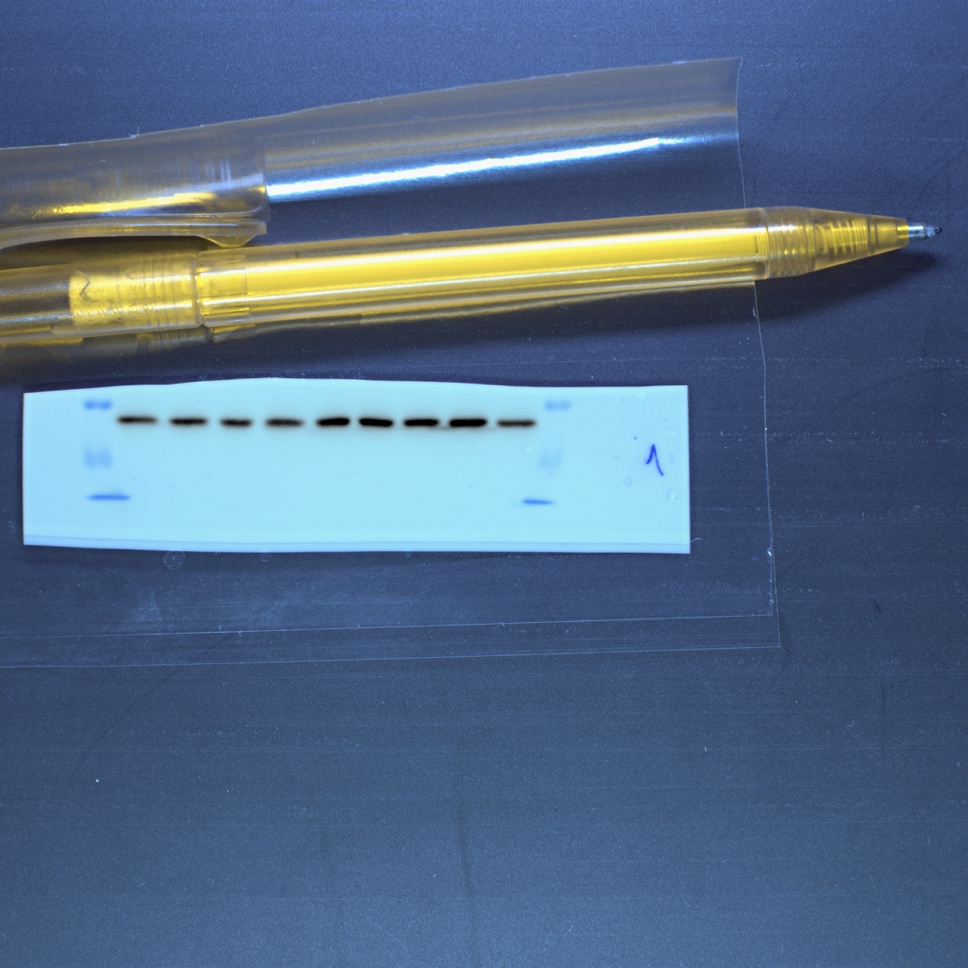

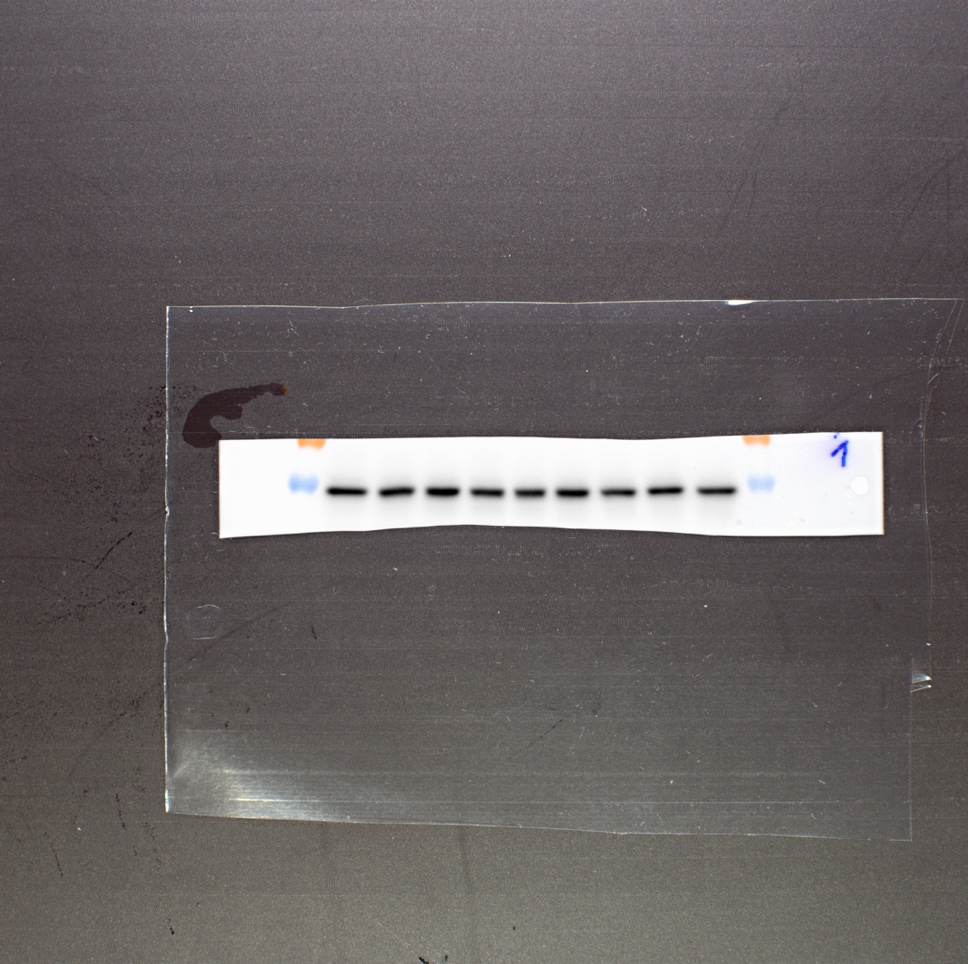

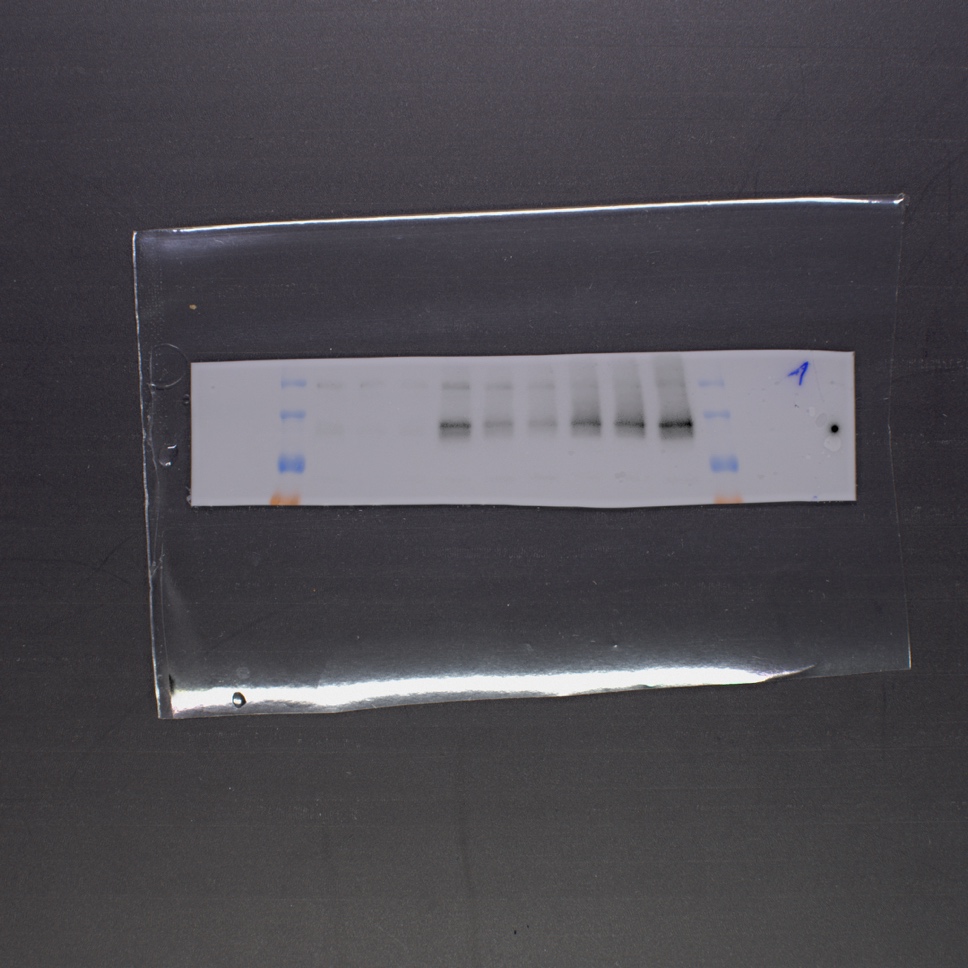
**

HIF1A

P53

RPLP01

V4 wt 24 hours

**
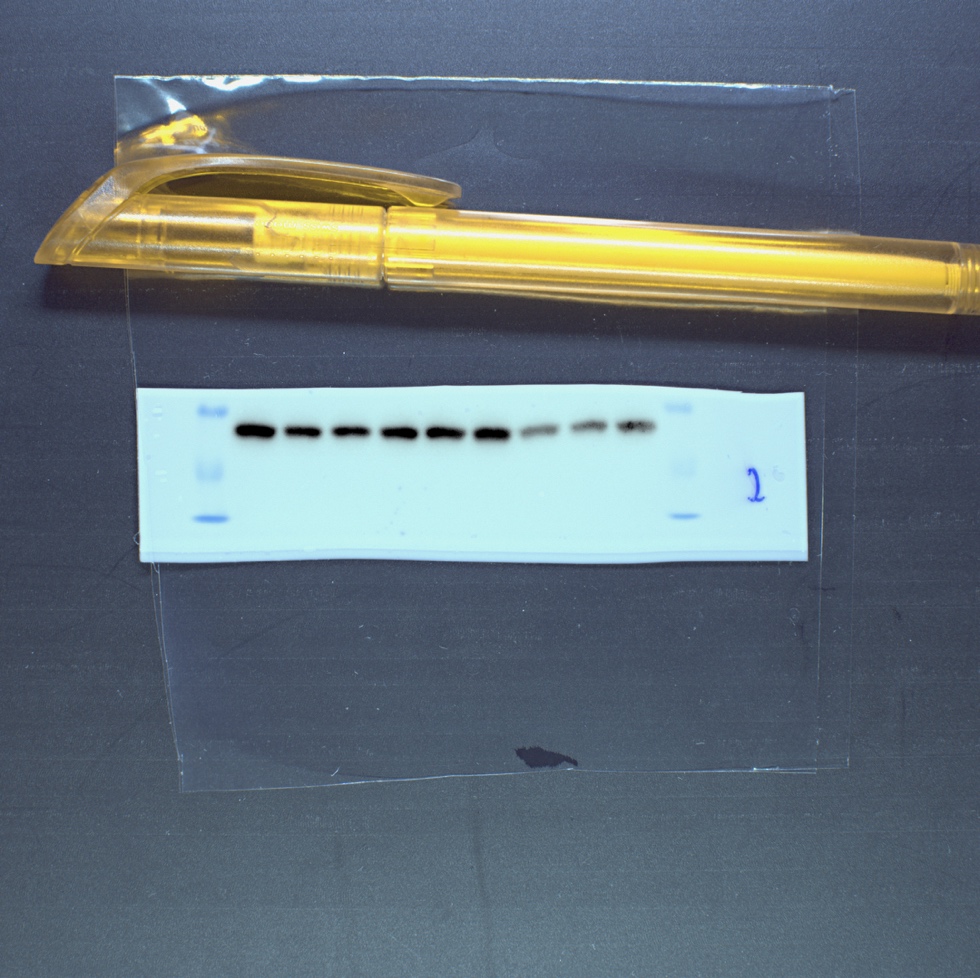

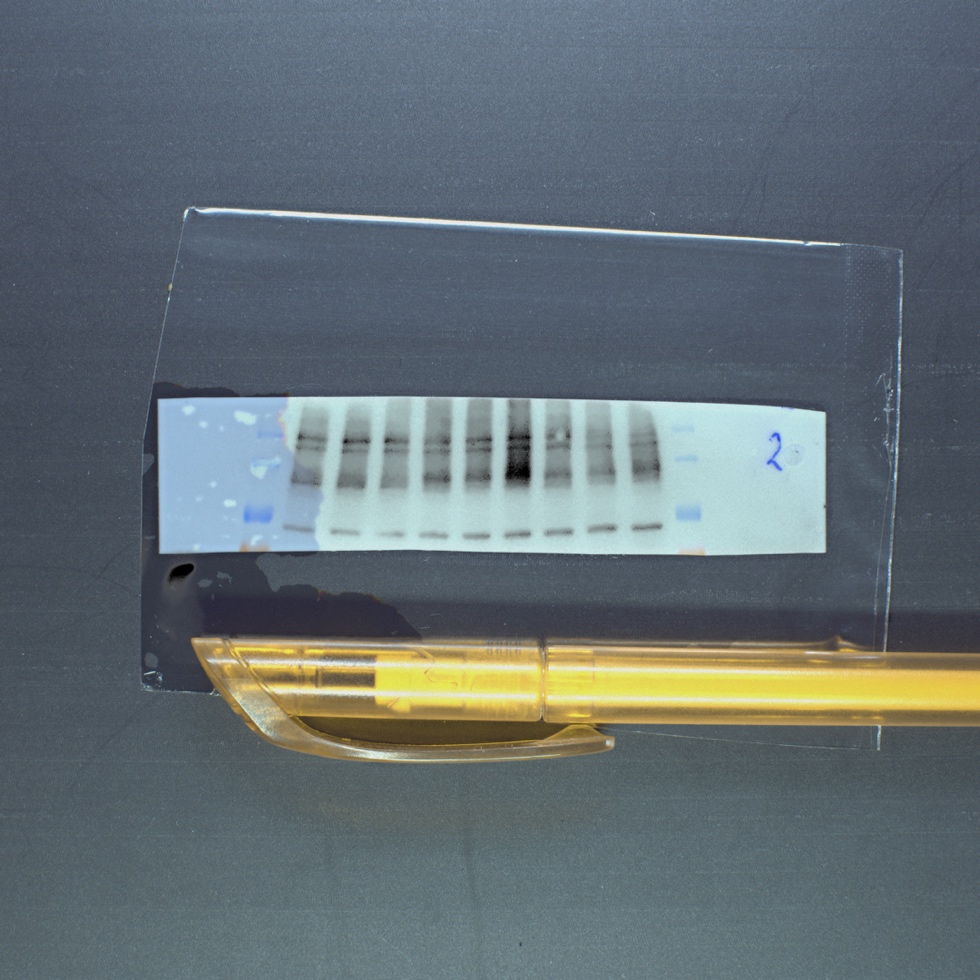

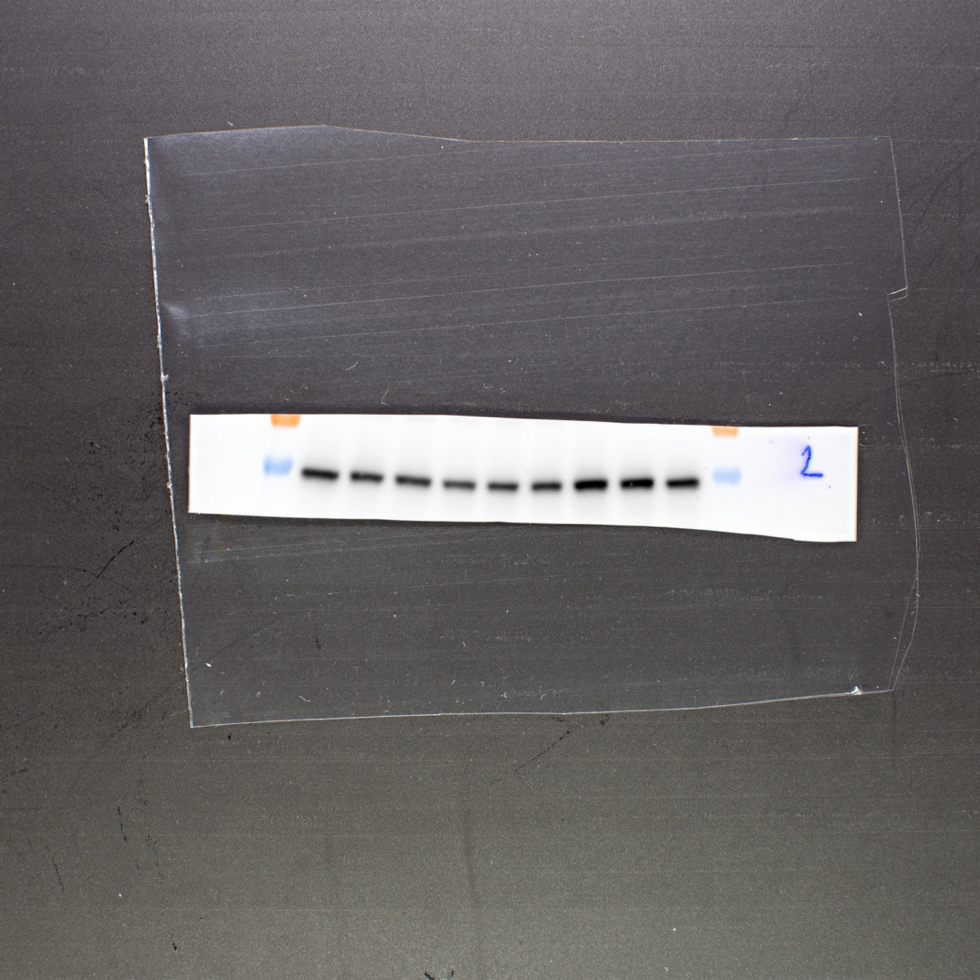
**

HIF1A

P53

RPLP01

# Western Blots for Figure 1 B/C

V1 wt 4 hours – phospho p53

**
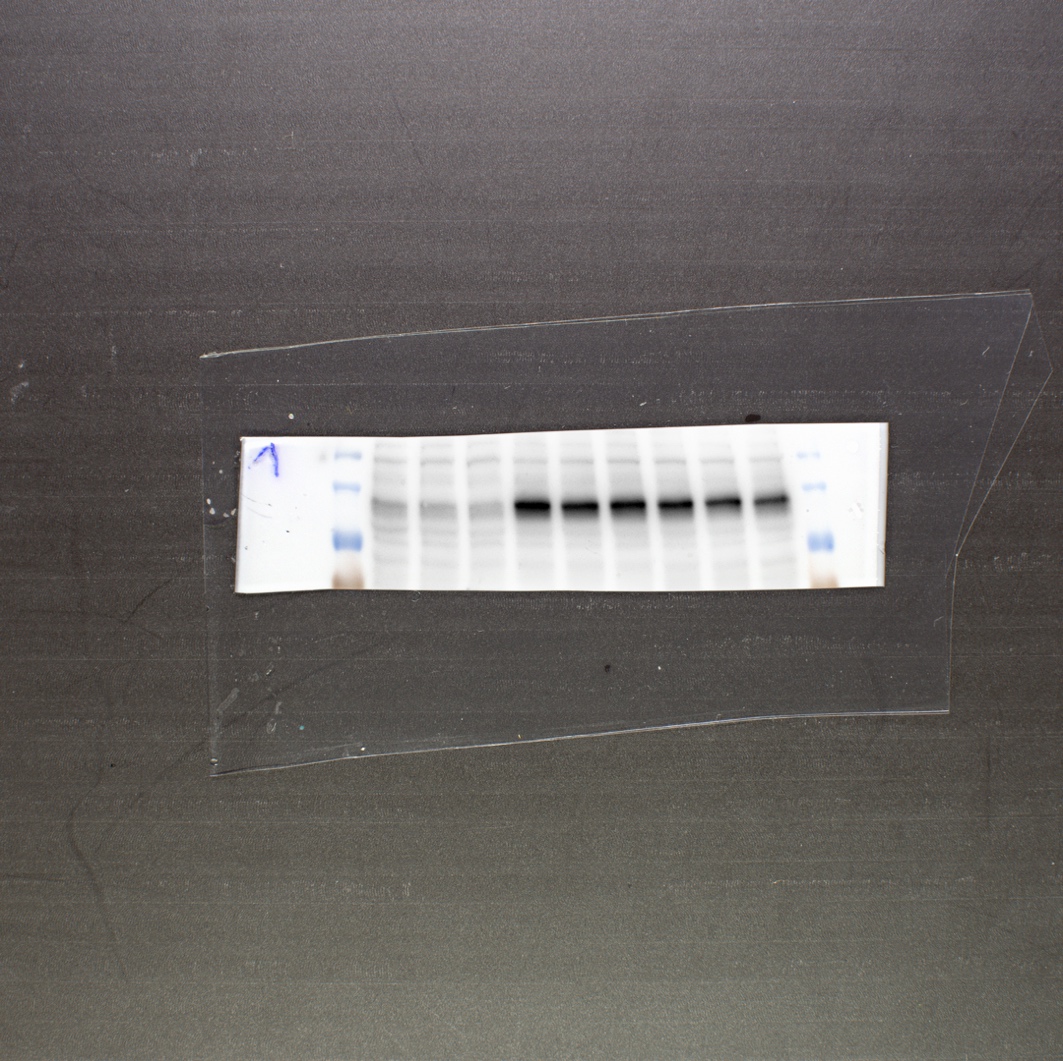
**

**
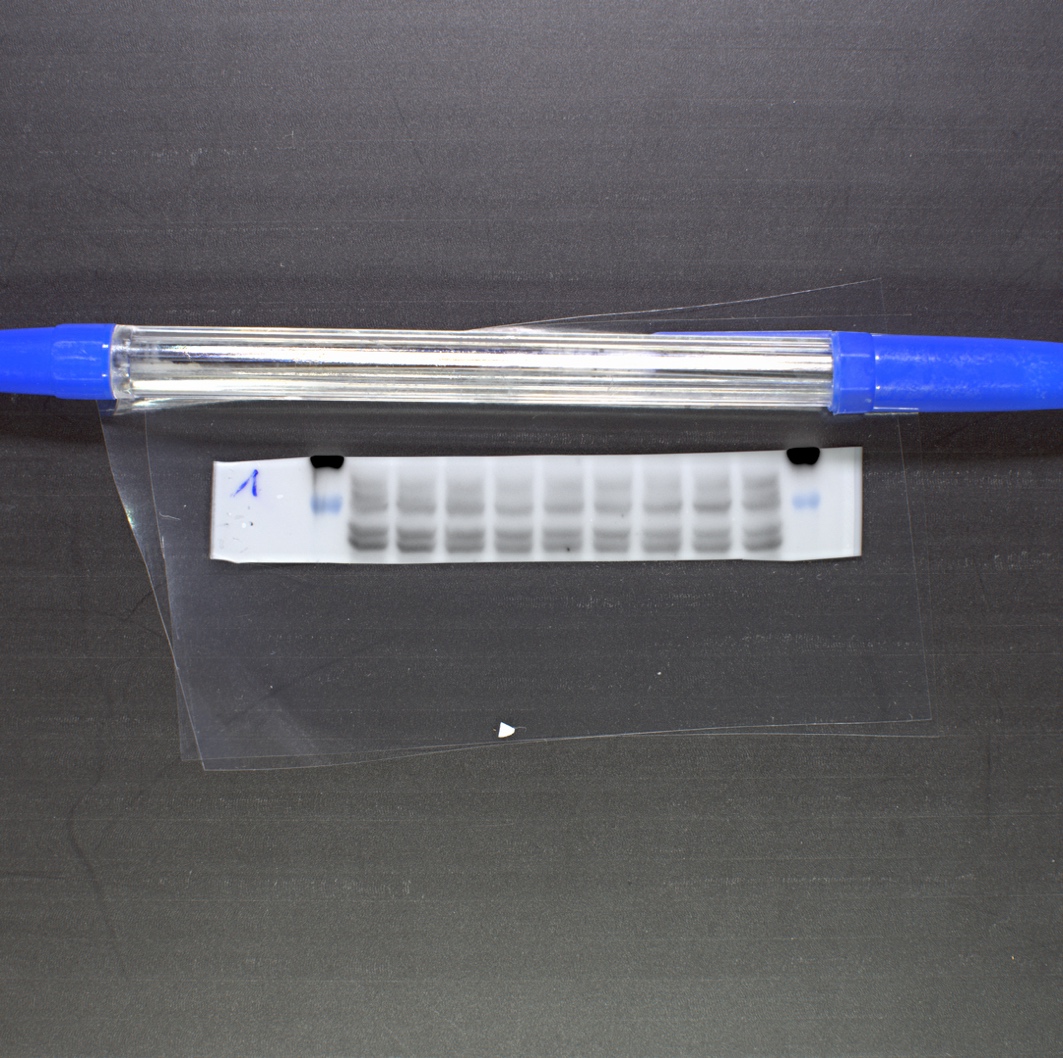

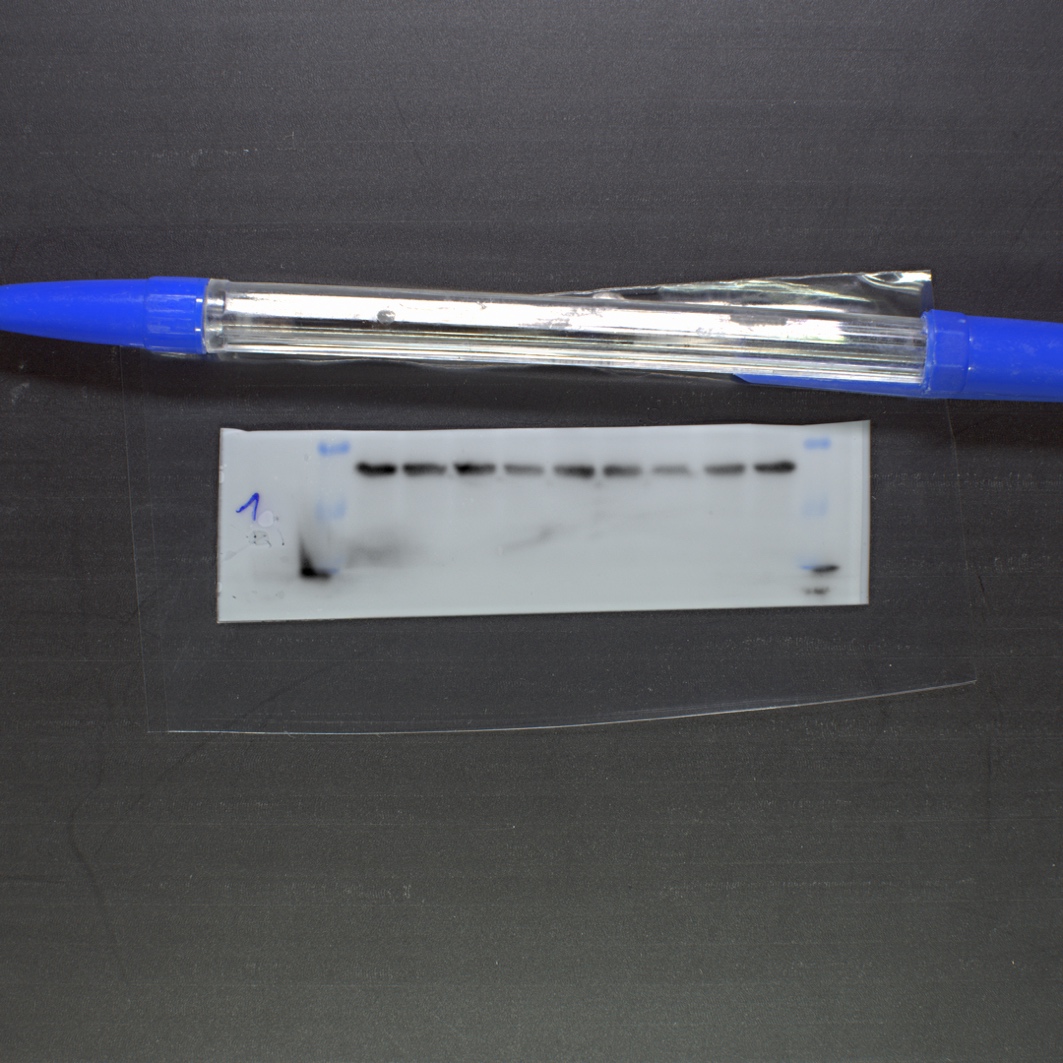
**

Phospho P53

RPLP01

HIF1A

V1 wt 24 hours - phospho p53

HIF1A

**
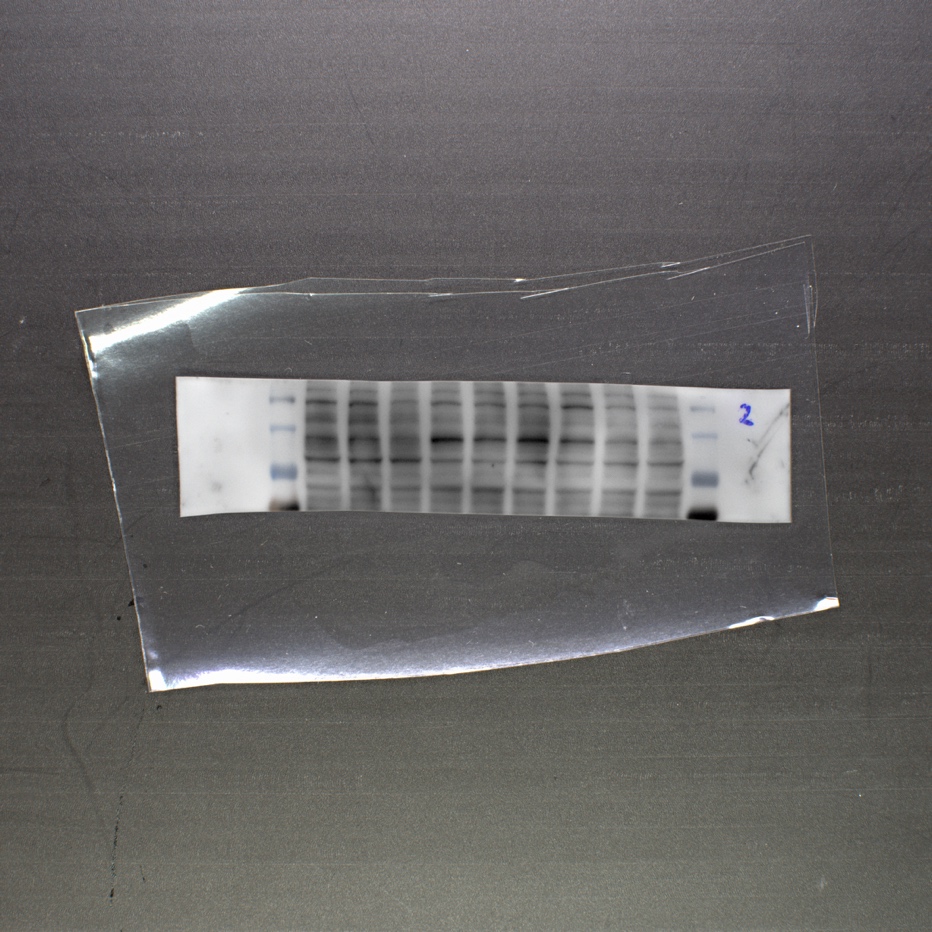
**

**
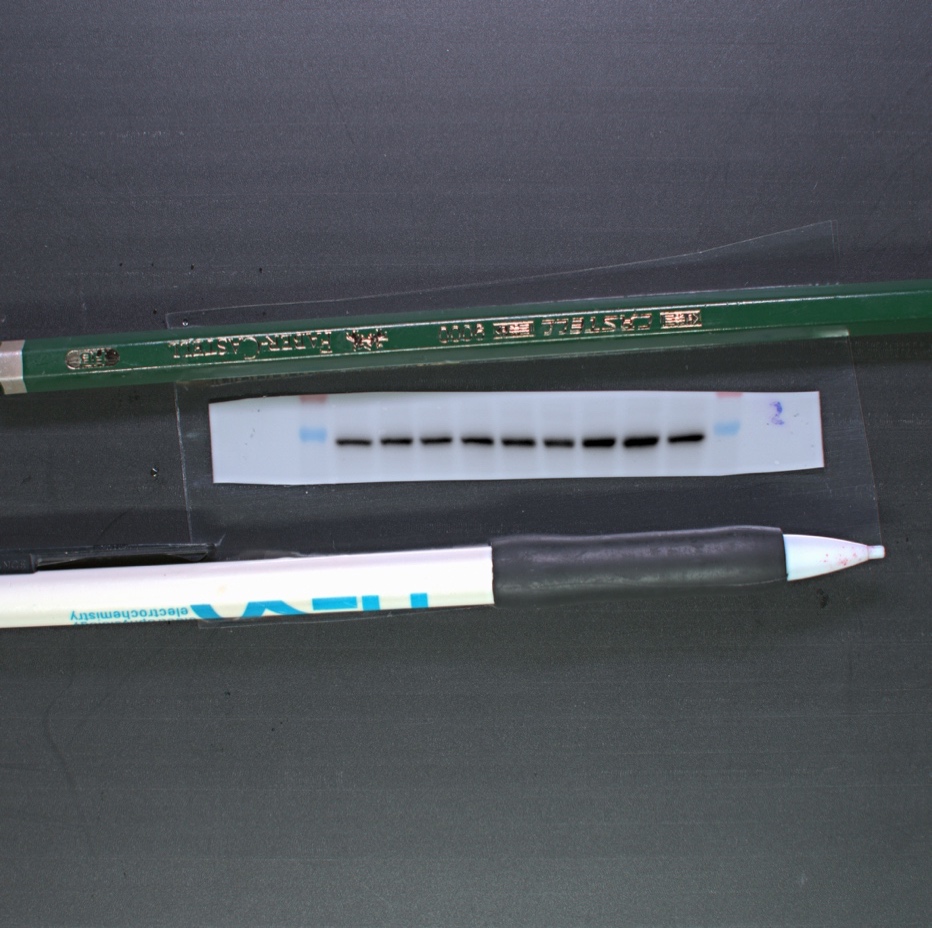

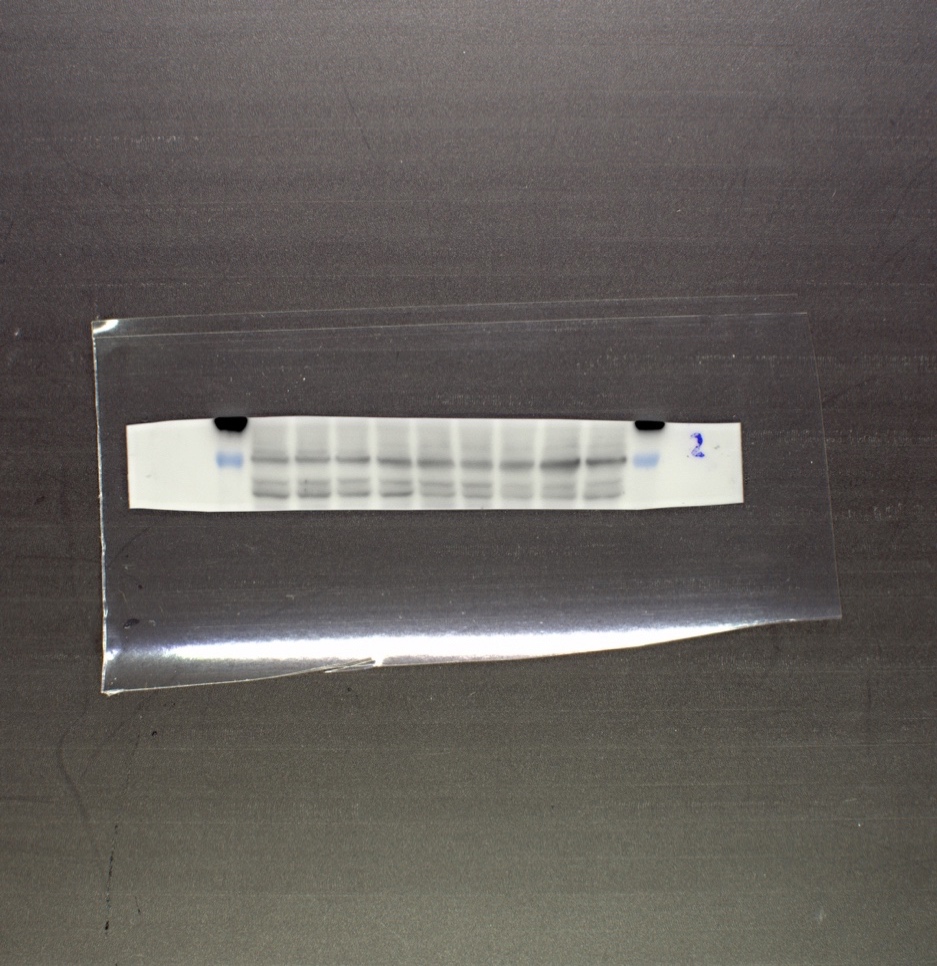
**

Phospho P53

P53

**
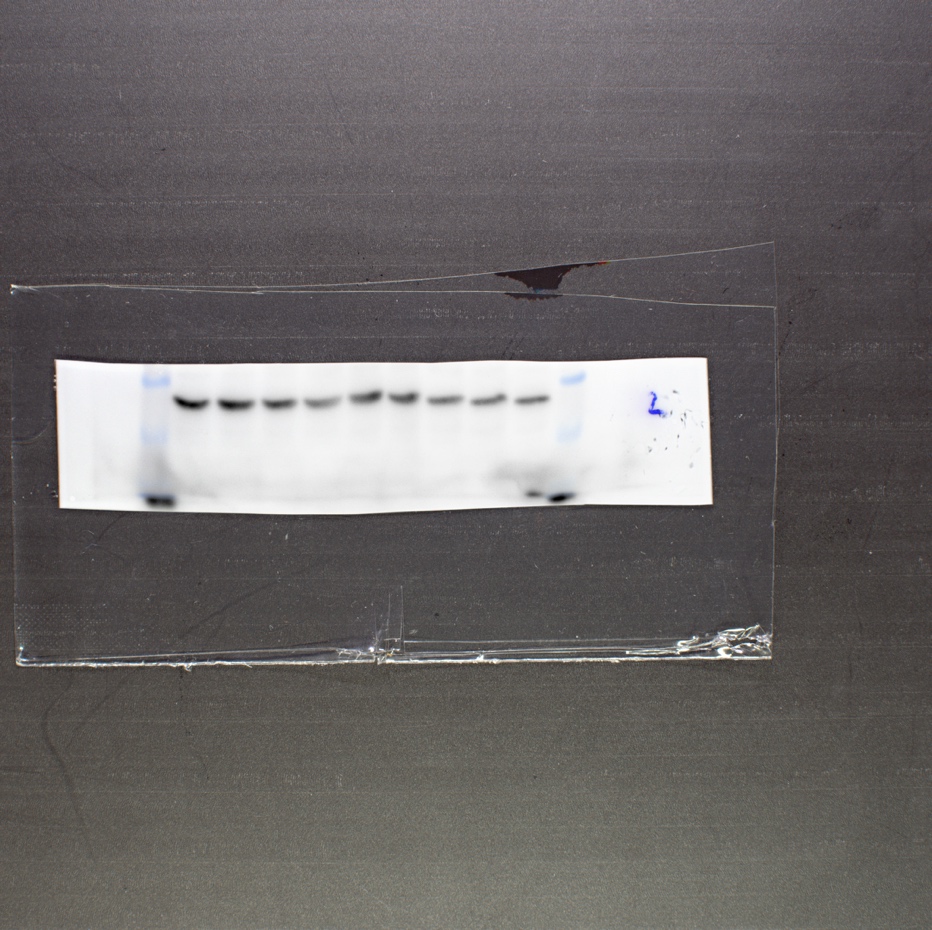
**

RPLP01

**
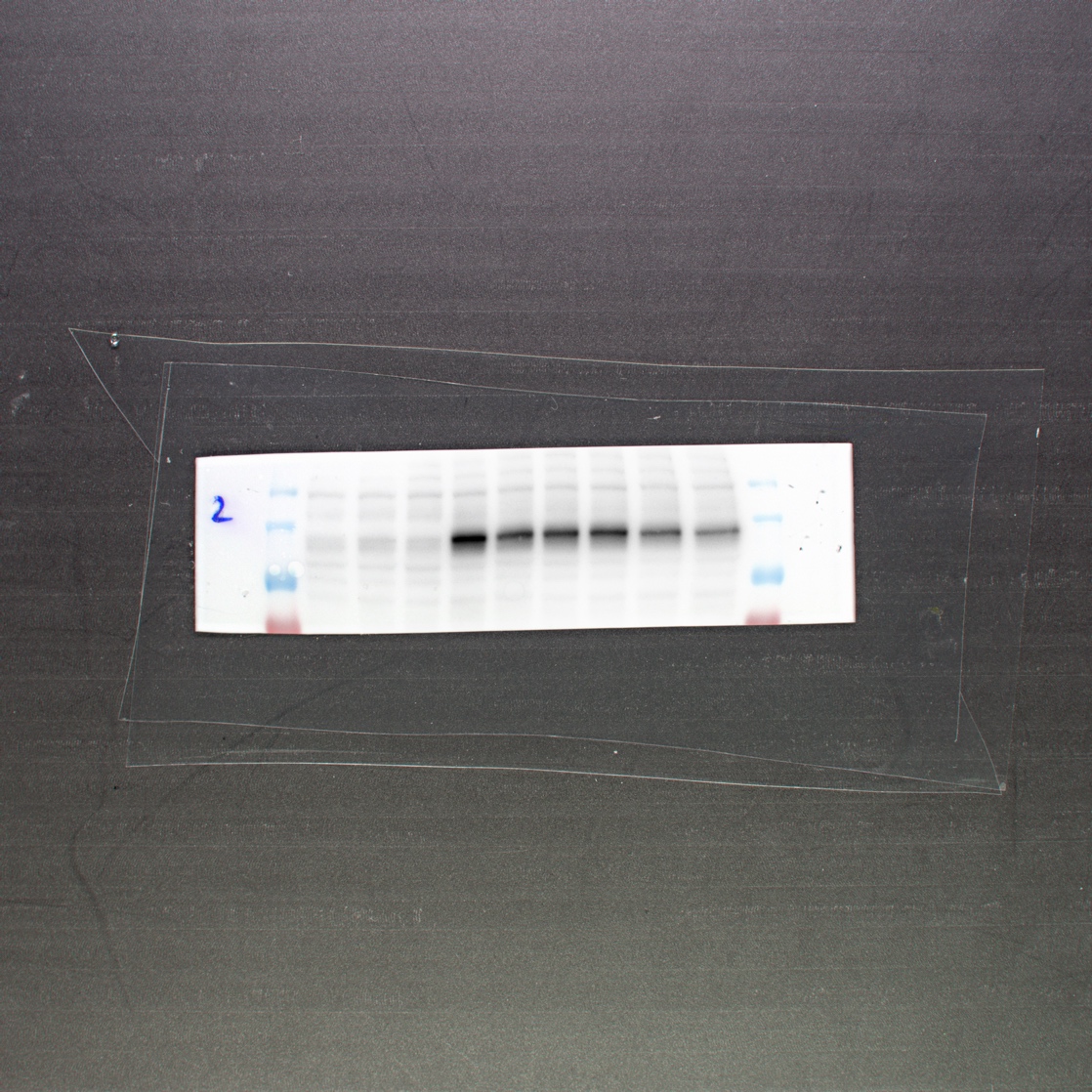
**

V2 wt 4 hours – phospho p53

HIF1A

**
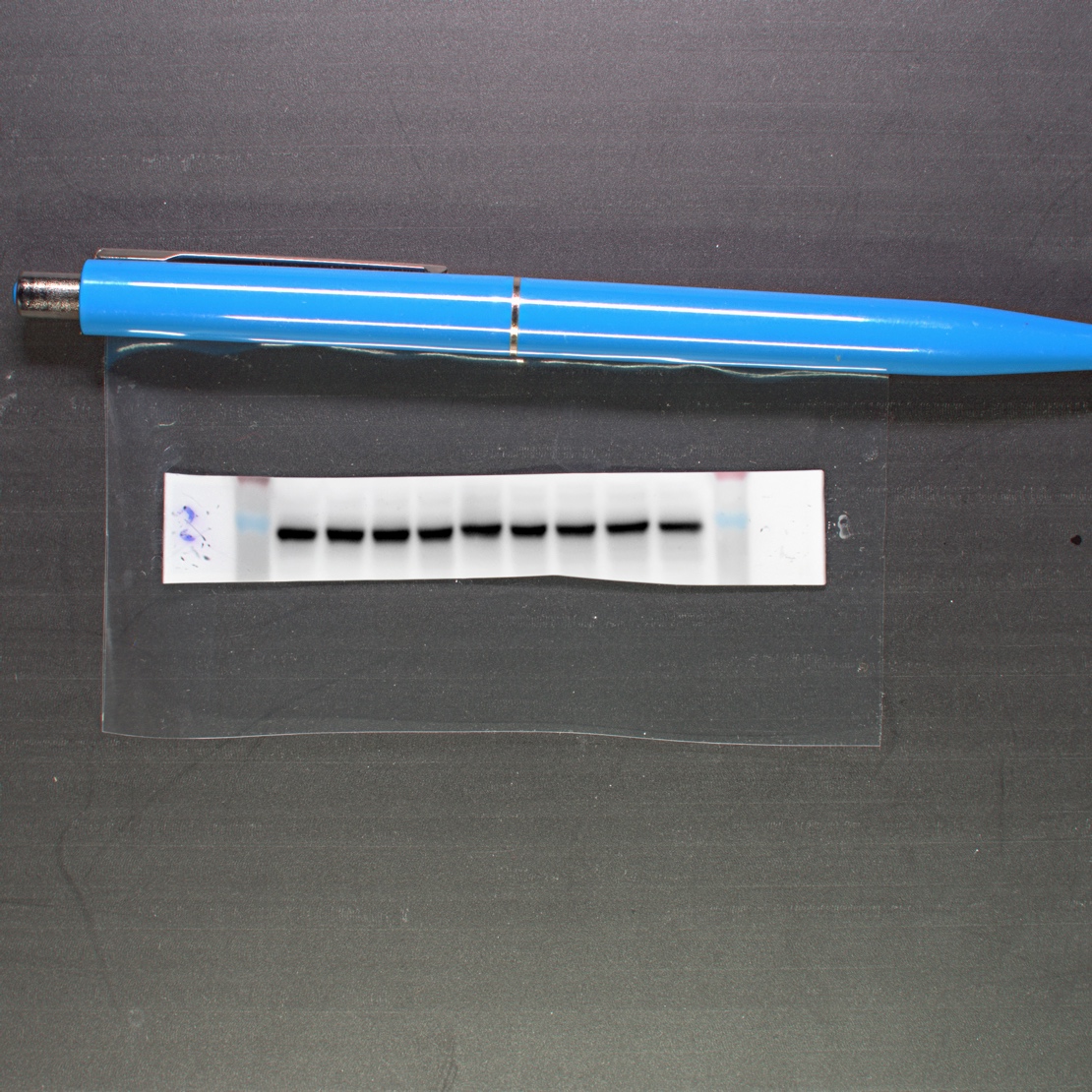
**

Phospho P53

**
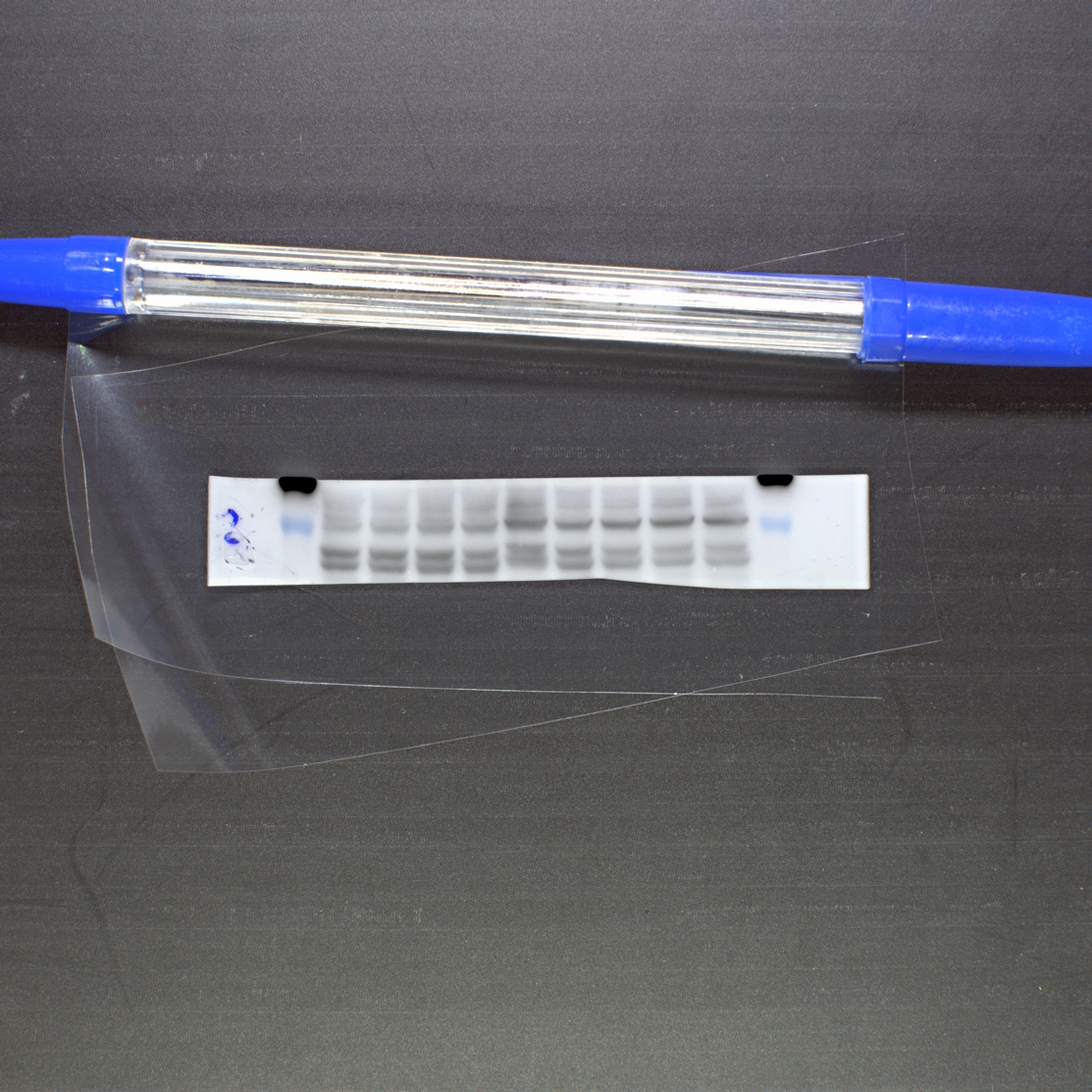
**

P53

**
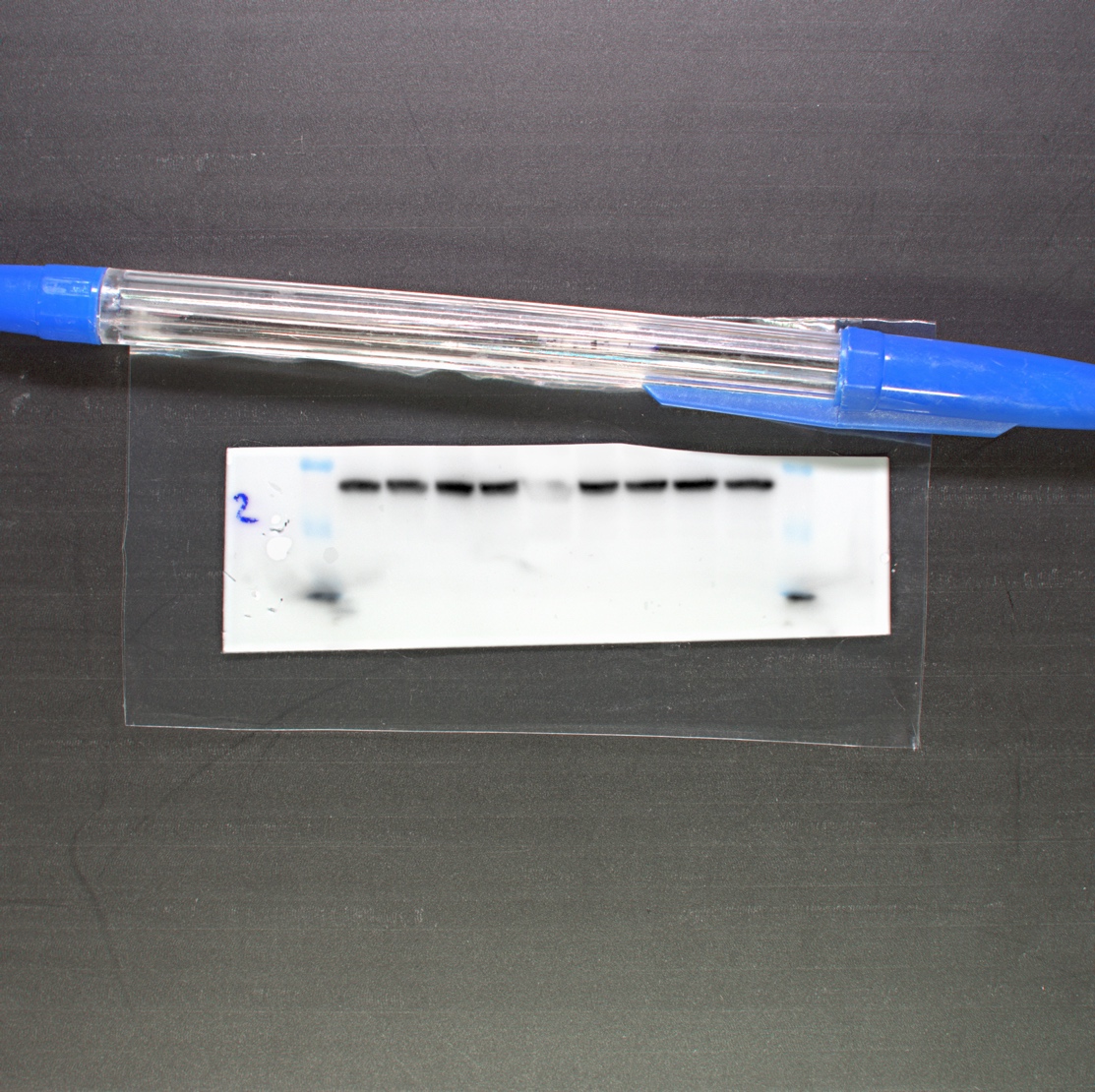
**

RPLP01

V2 wt 24 hours – phospho p53

**
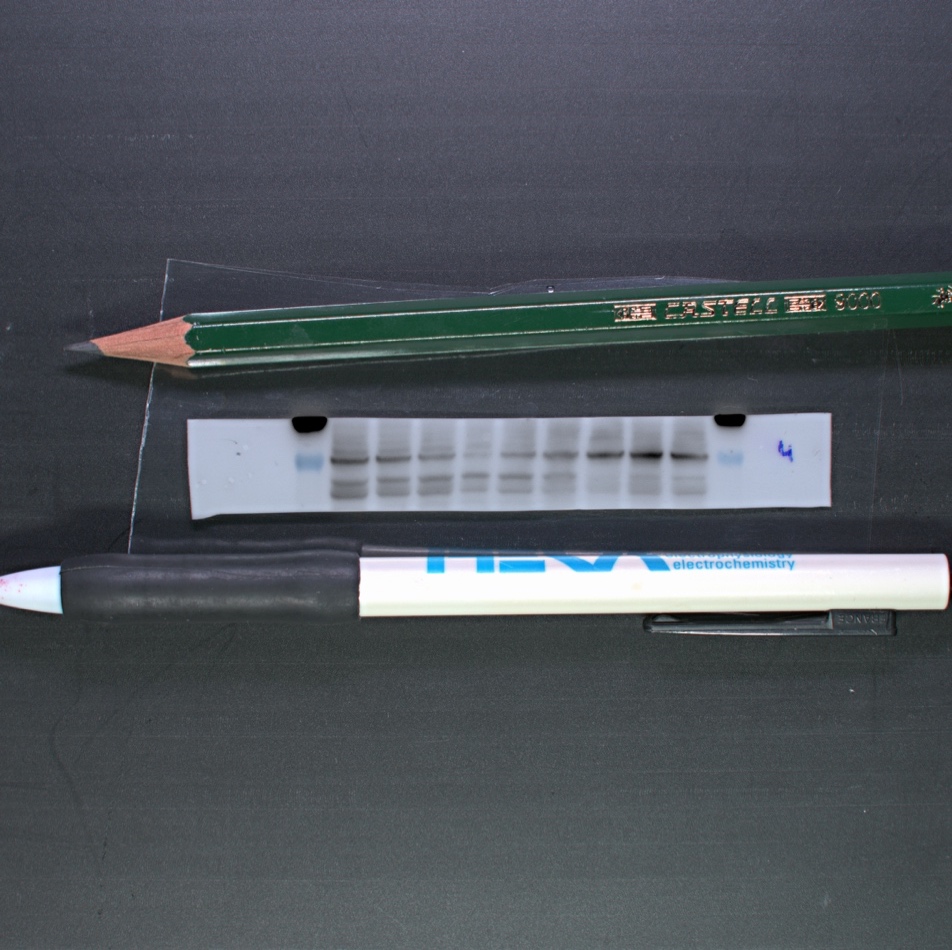

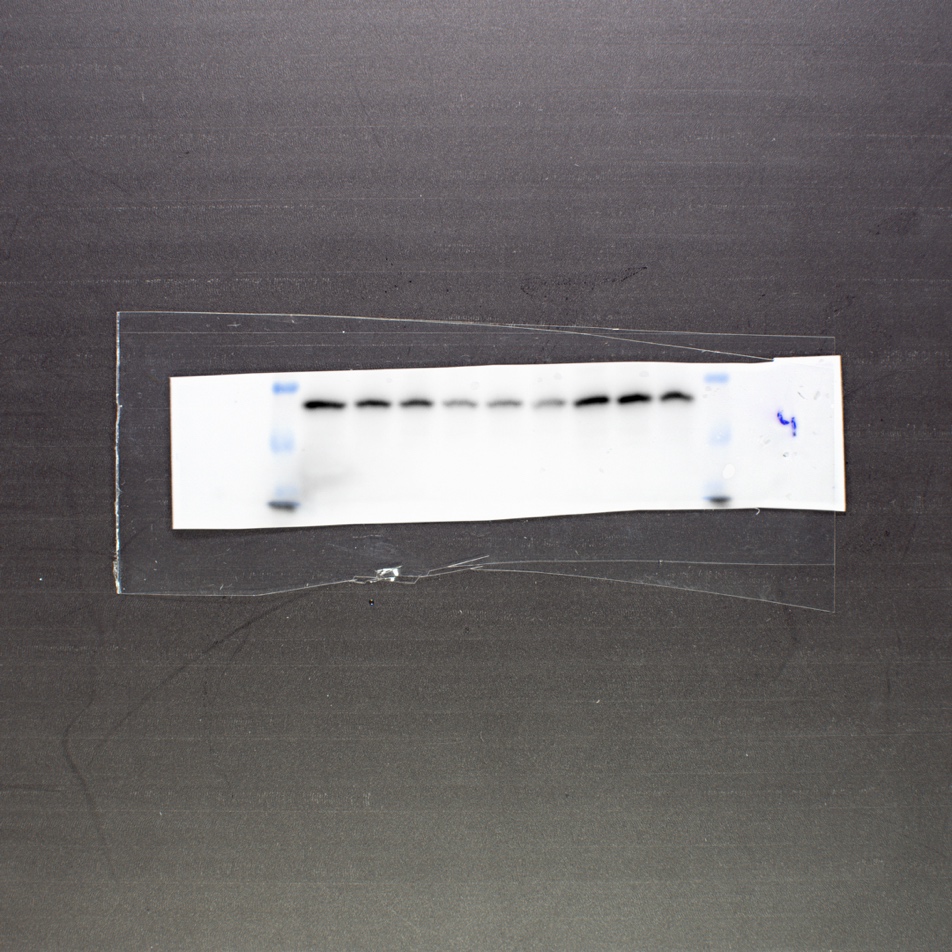

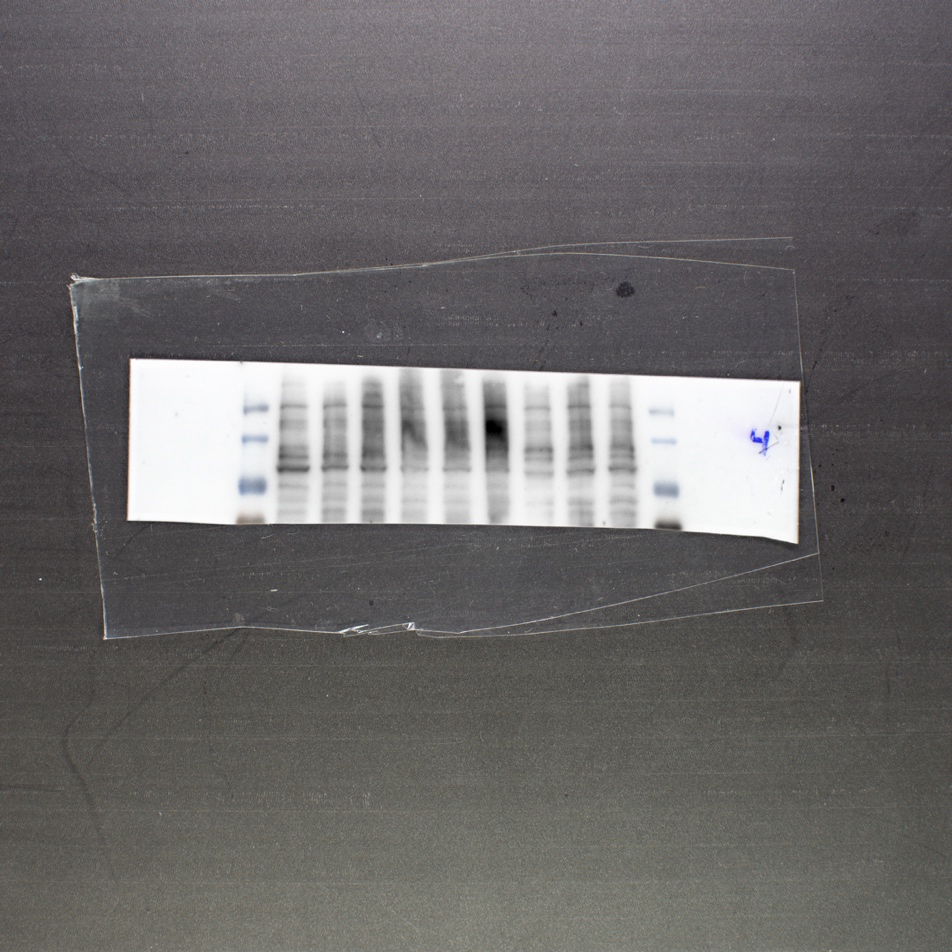
**

HIF1A

Phospho P53

**
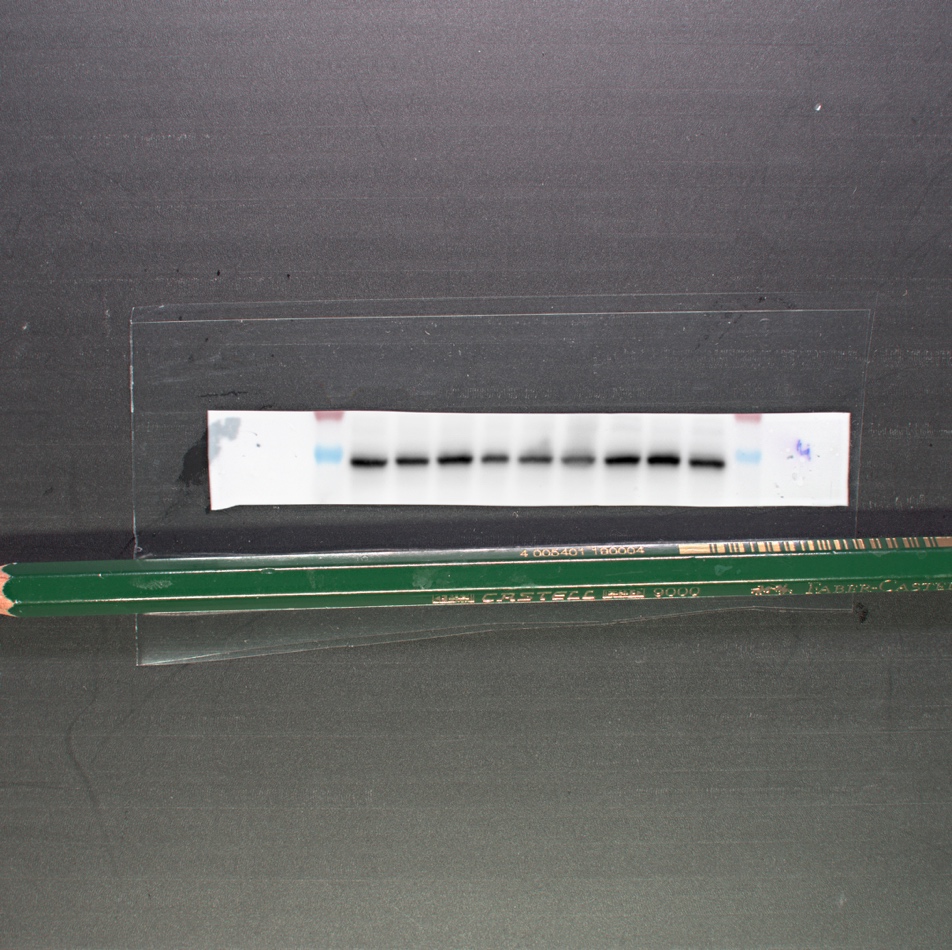
**

P53

RPLP01

V3 wt 4 hours – phospho p53

**
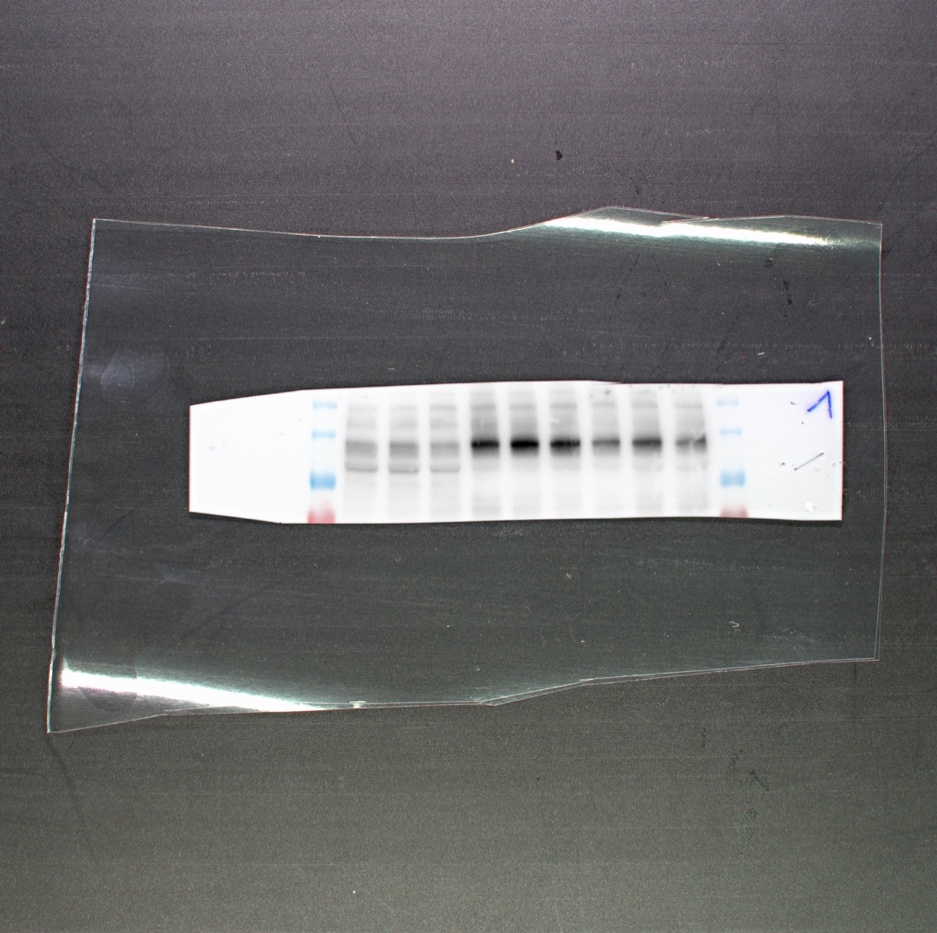
**

HIF1A

Phospho P53

**
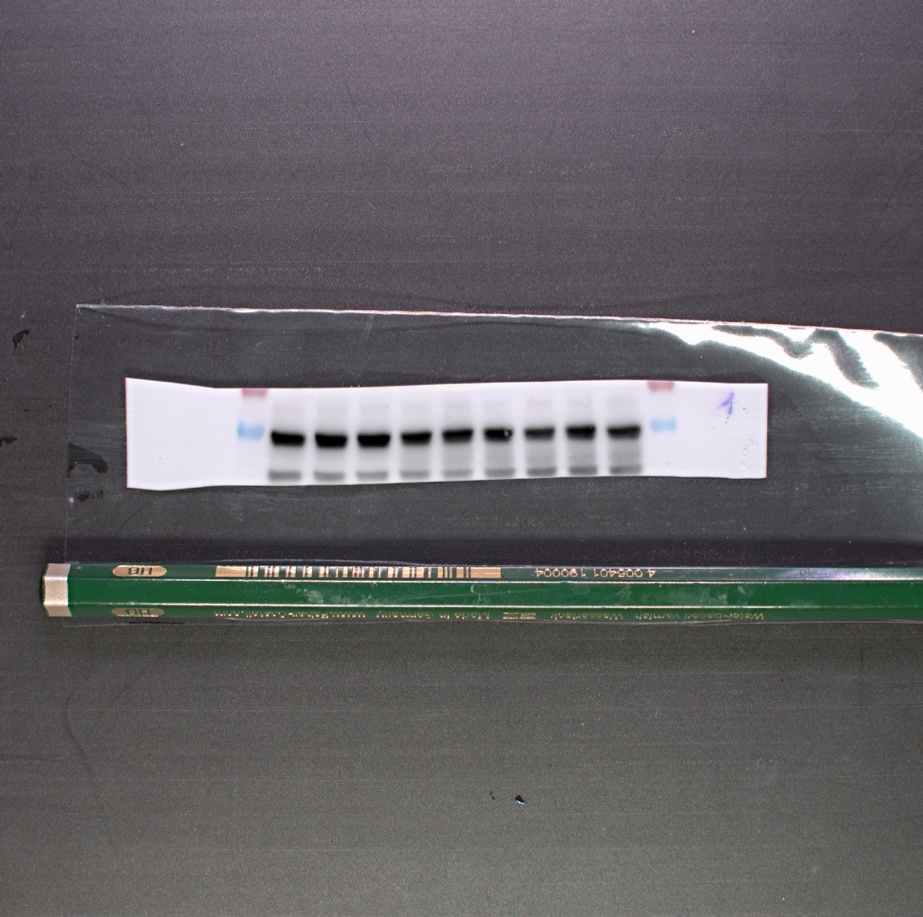

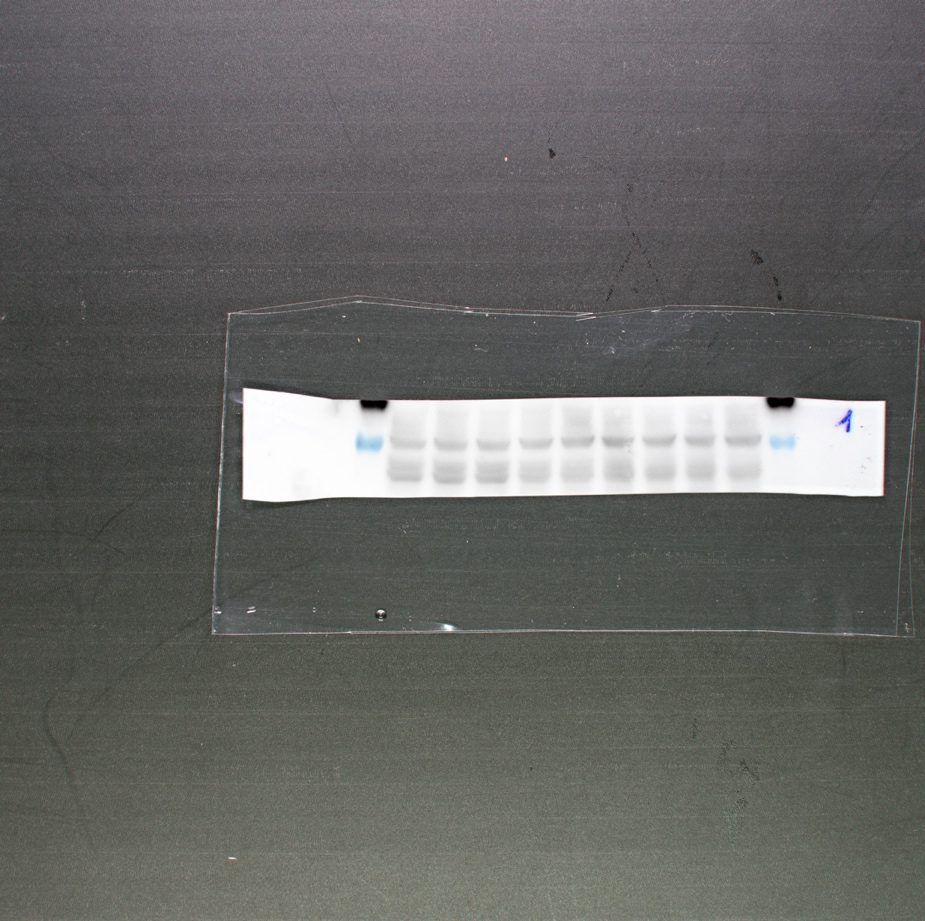
**

P53

**
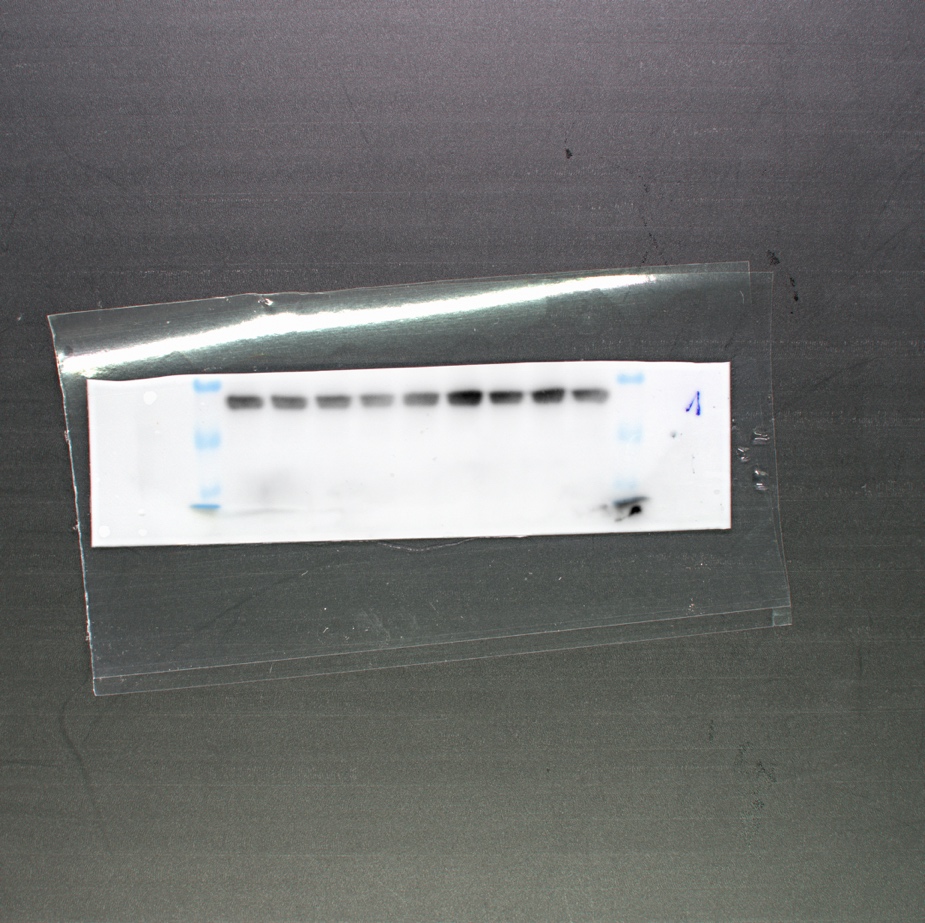
**

RPLP01

V3 wt 24 hours – phospho p53

**
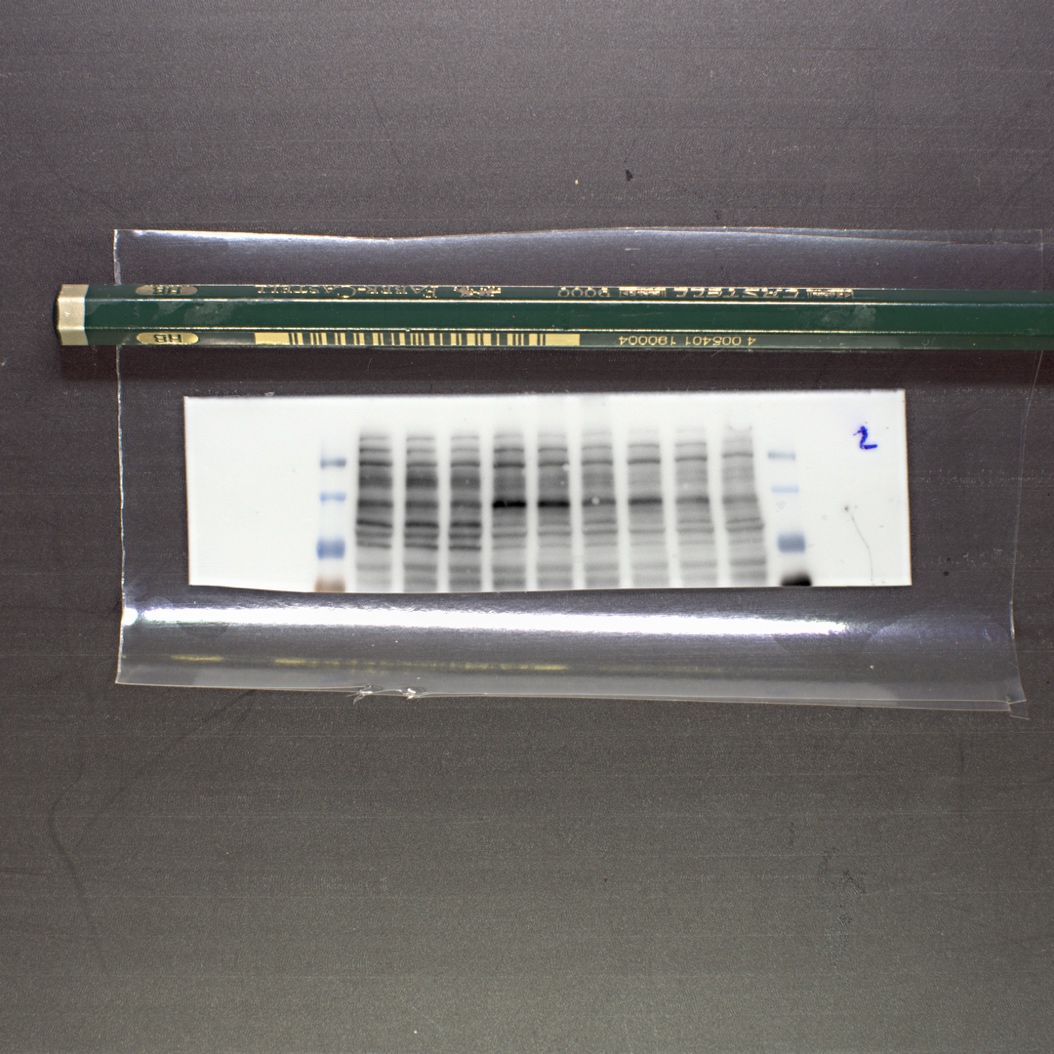
**

HIF1A

Phospho P53

**
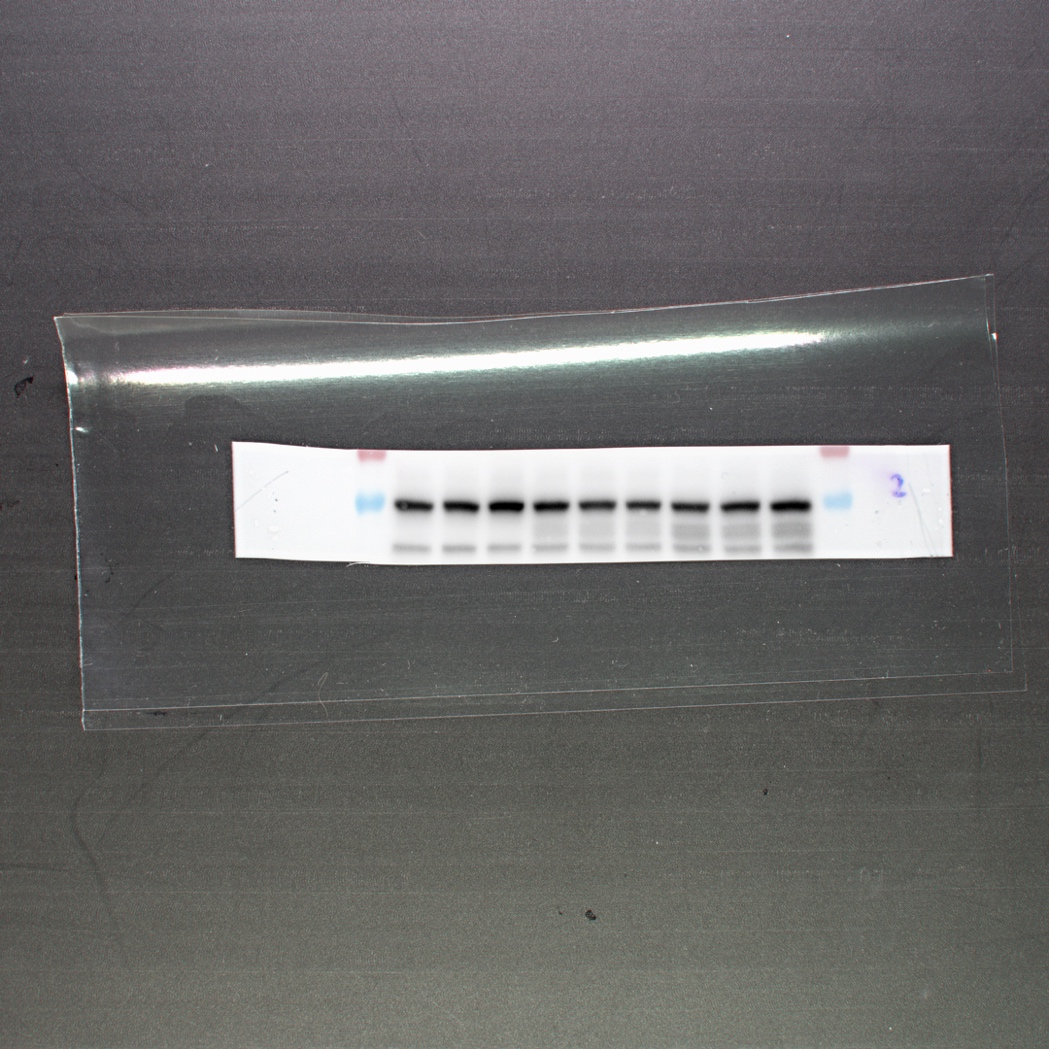

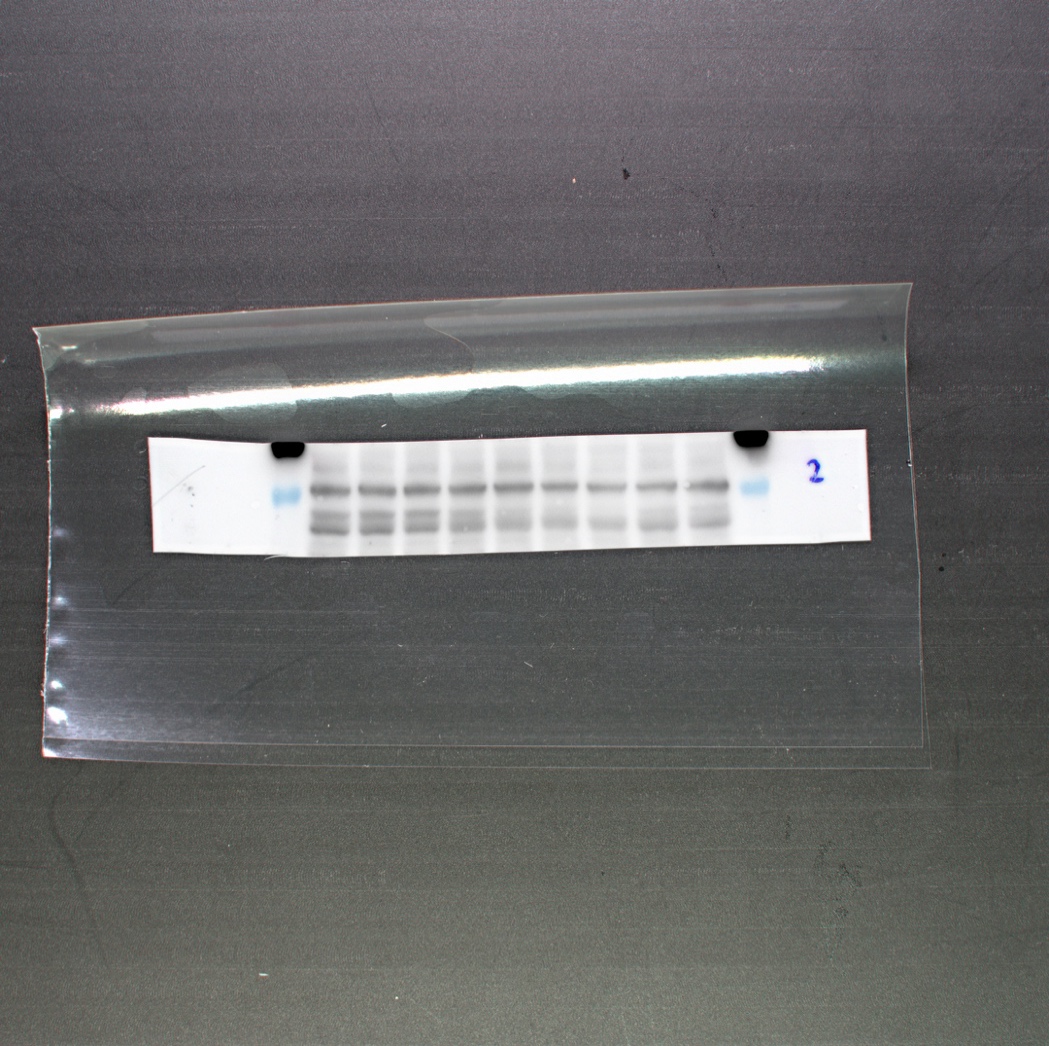
**

P53

**
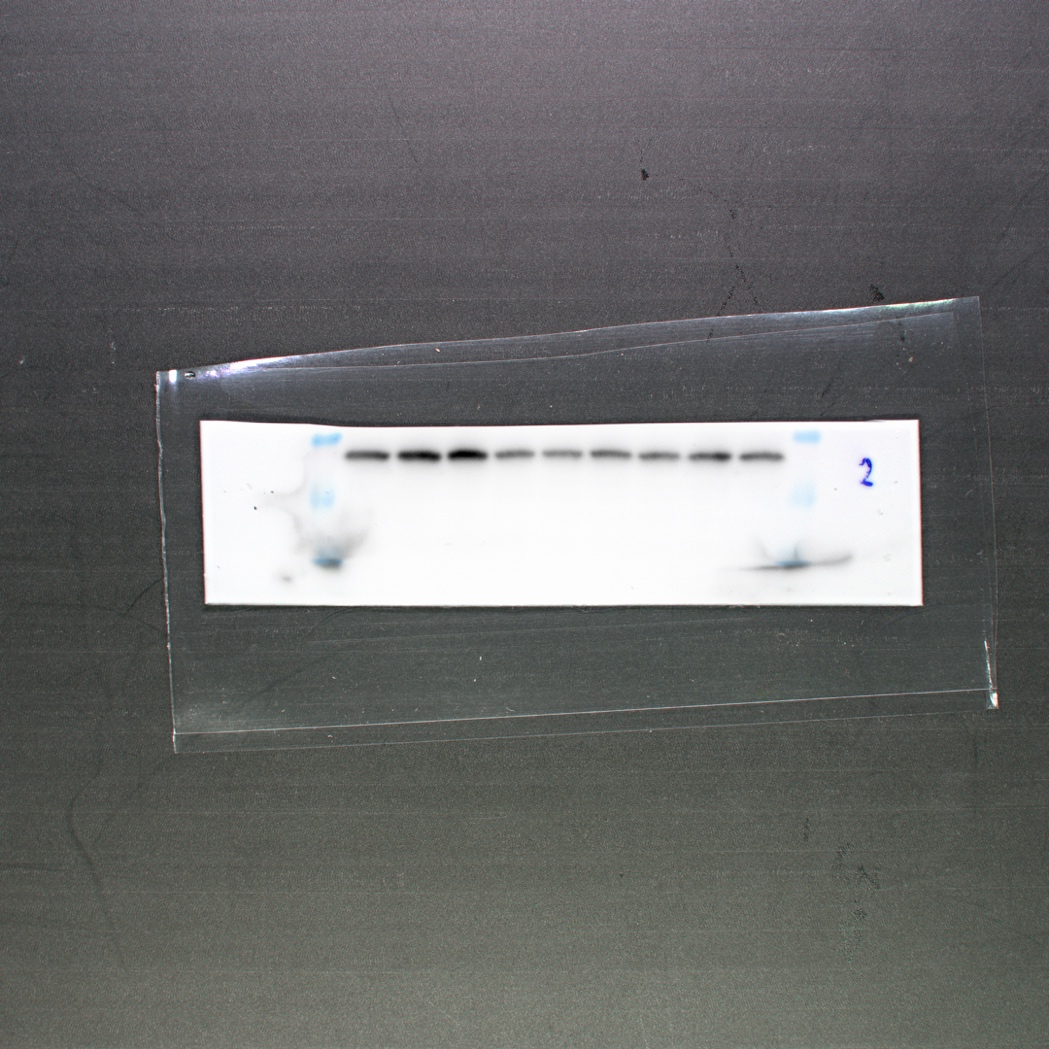
**

RPLP01

V4 wt 4 hours – phospho p53

**
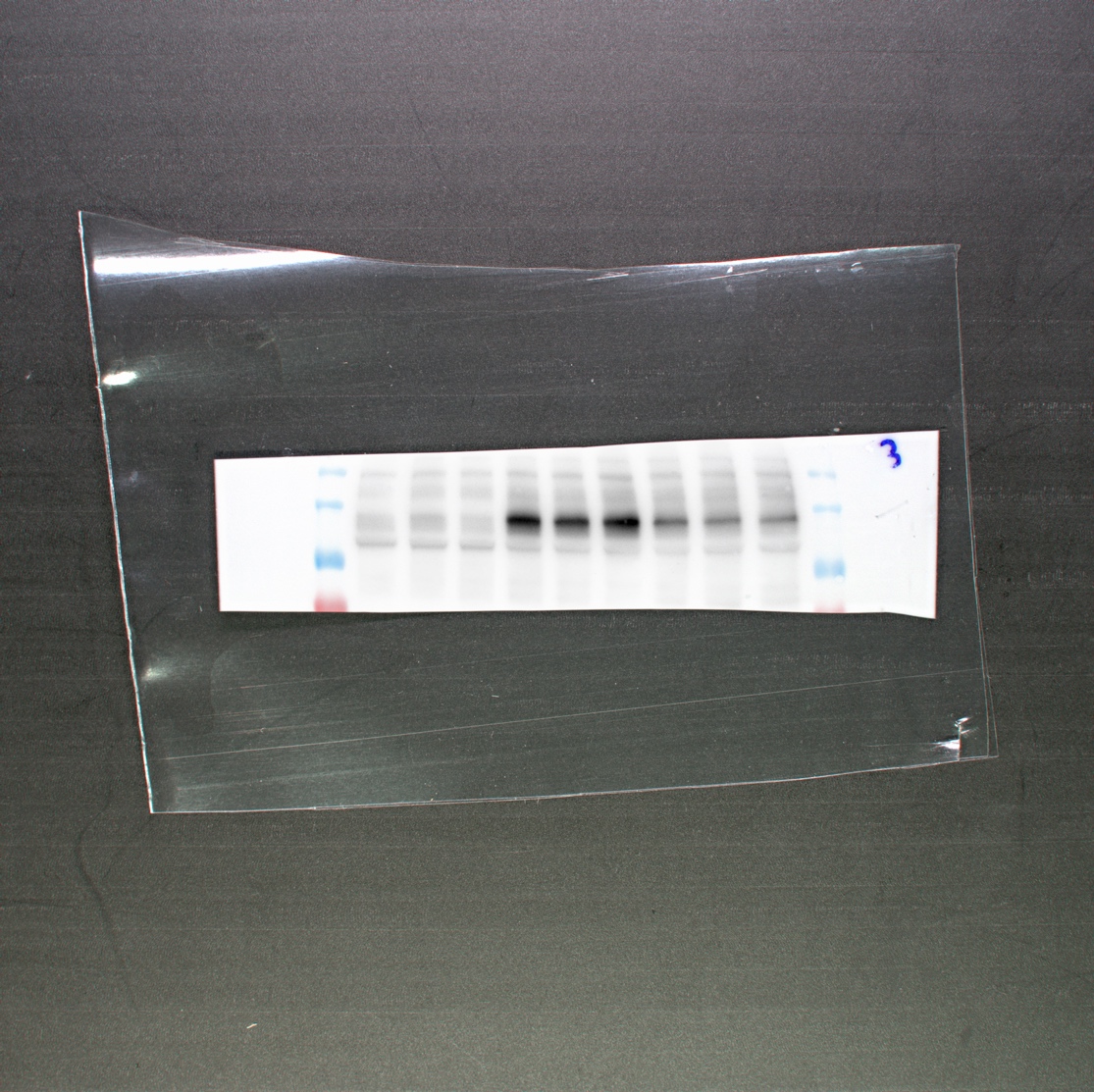
**

HIF1A

Phospho P53

**
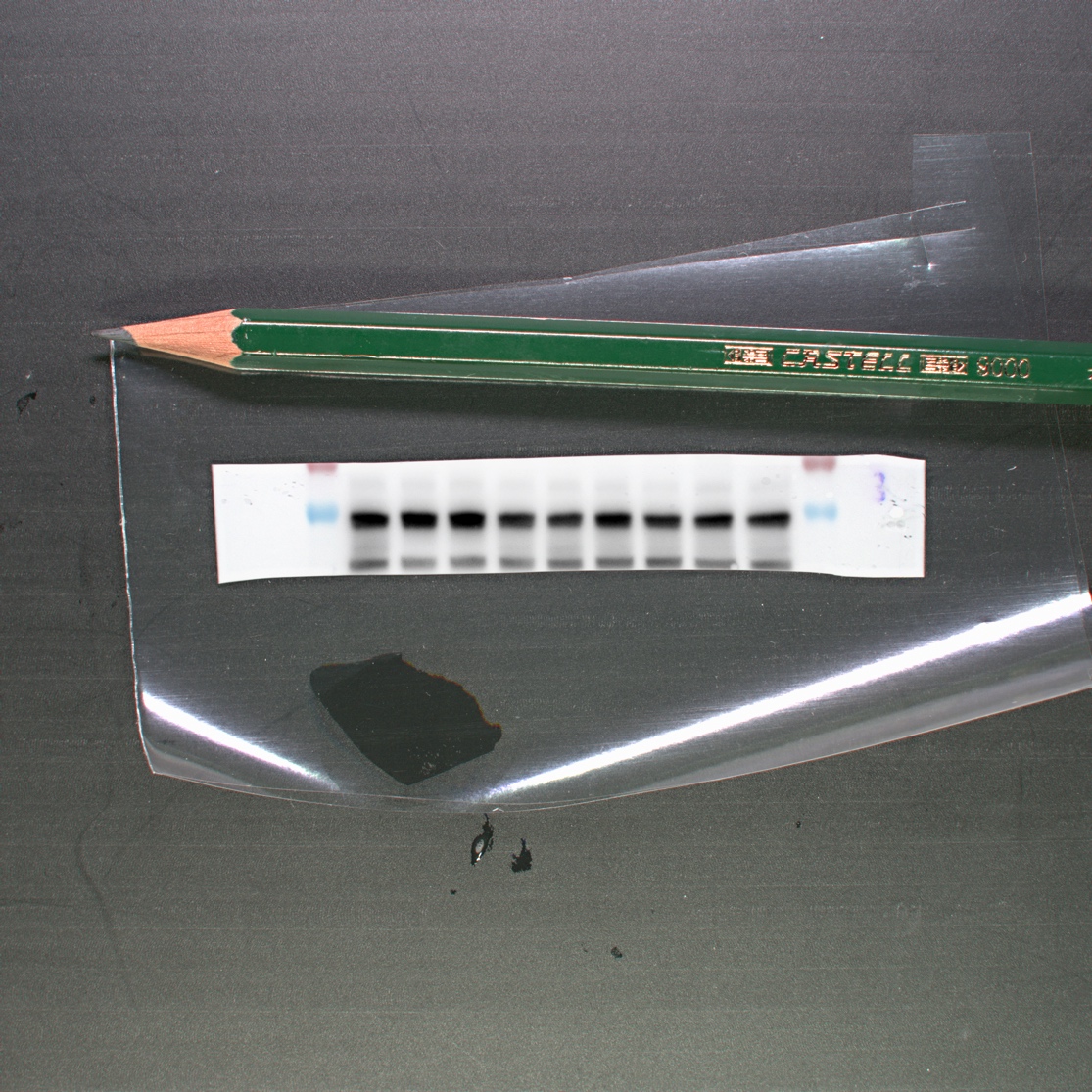

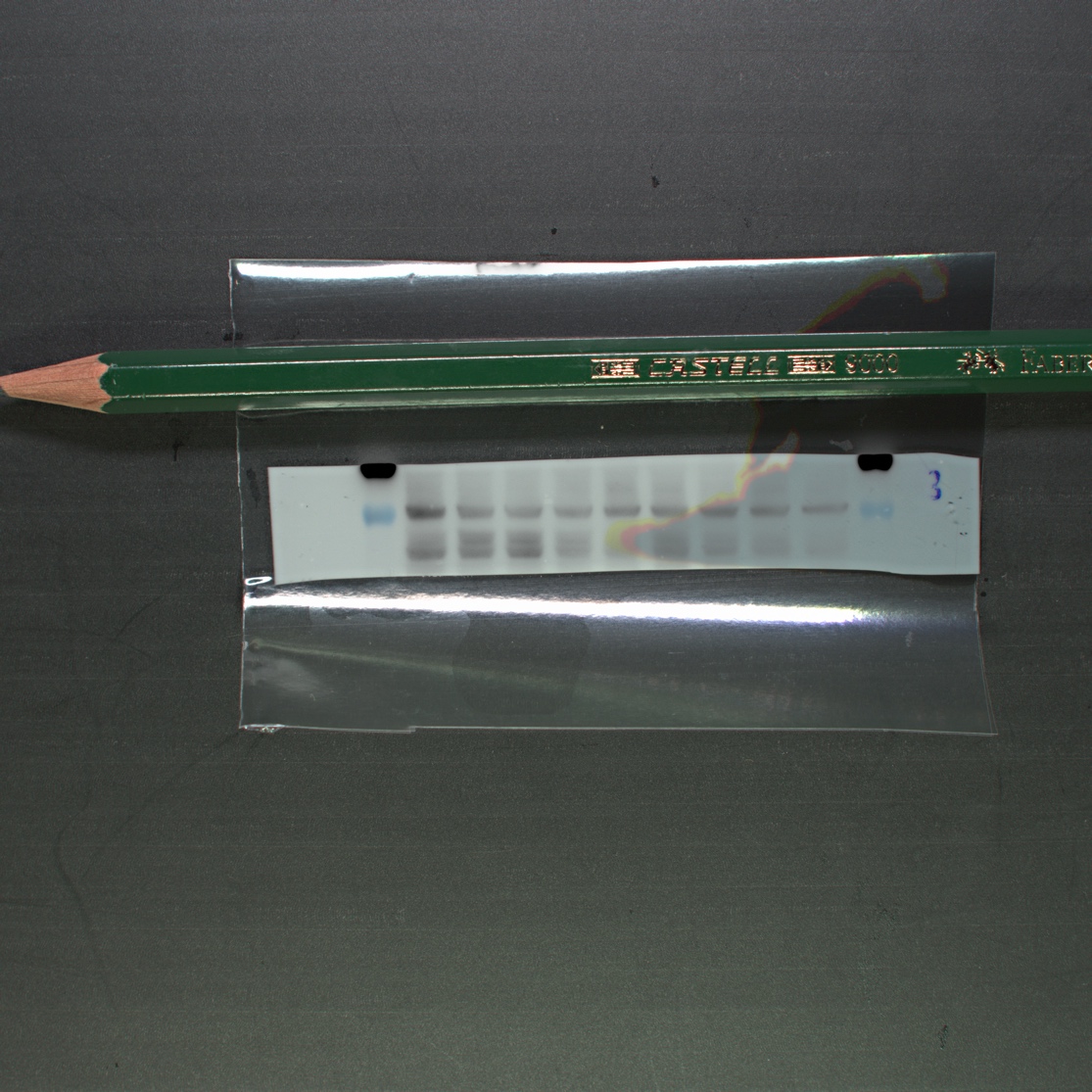
**

P53

**
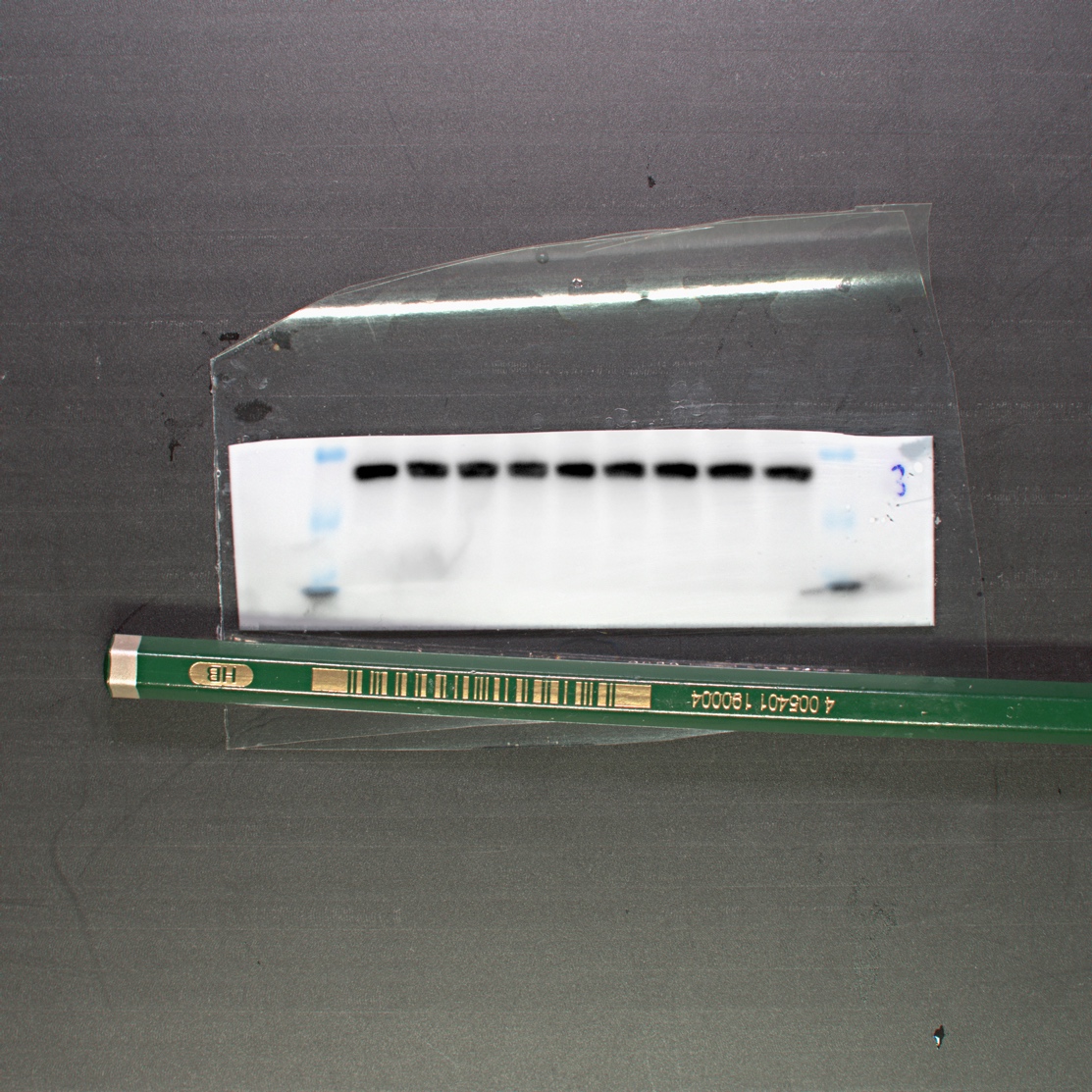
**

RPLP01

V4 wt 24 hours – phospho p53

**
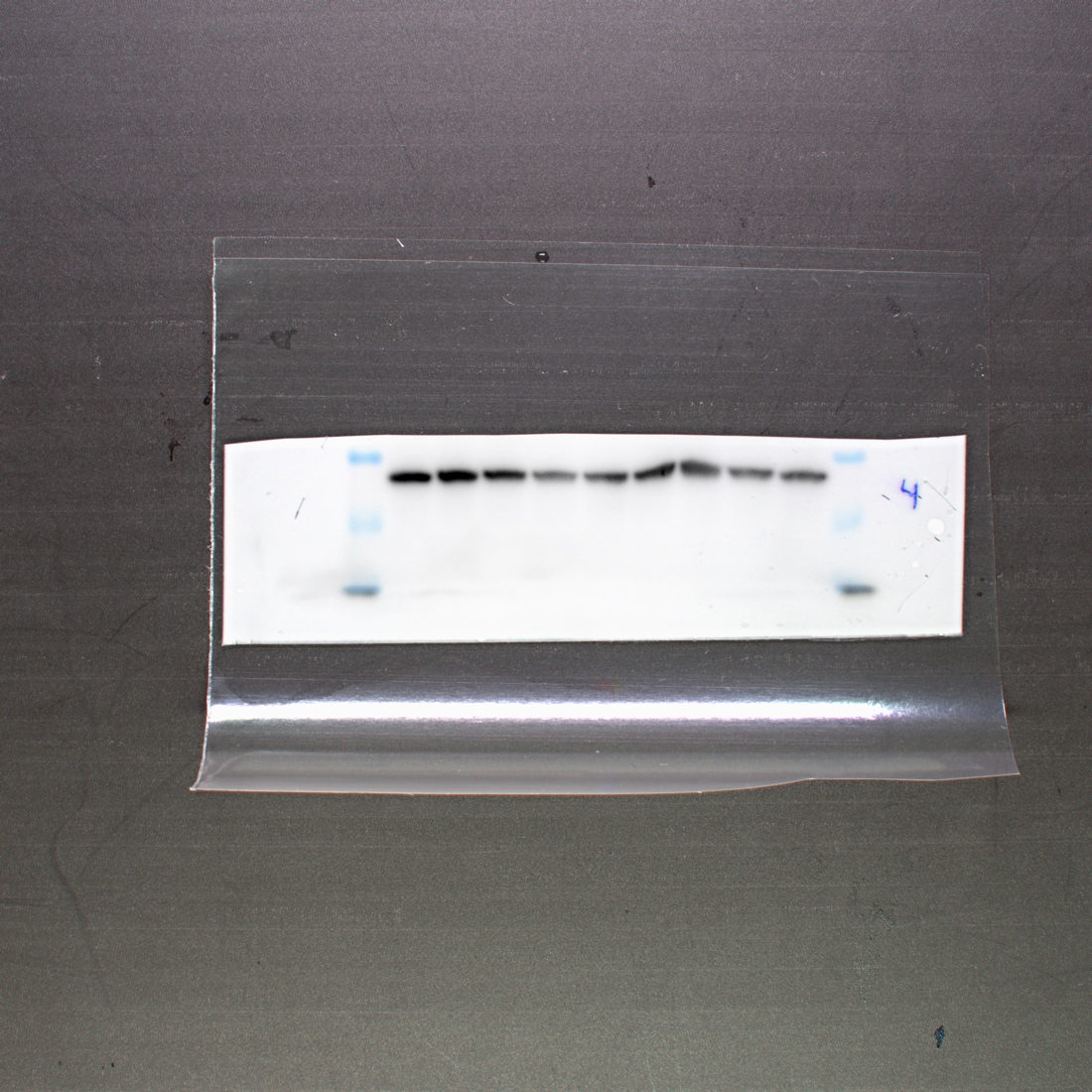

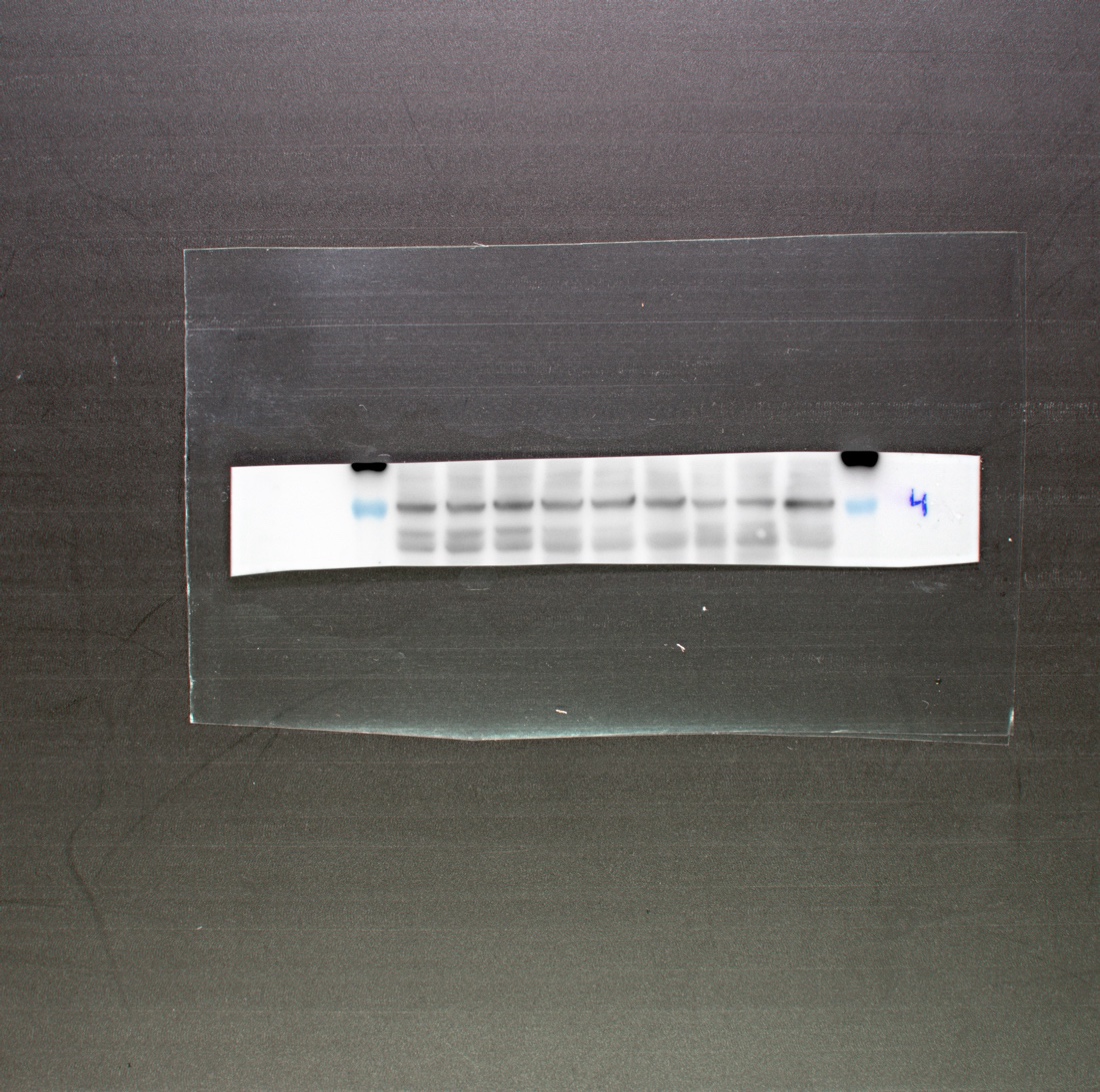

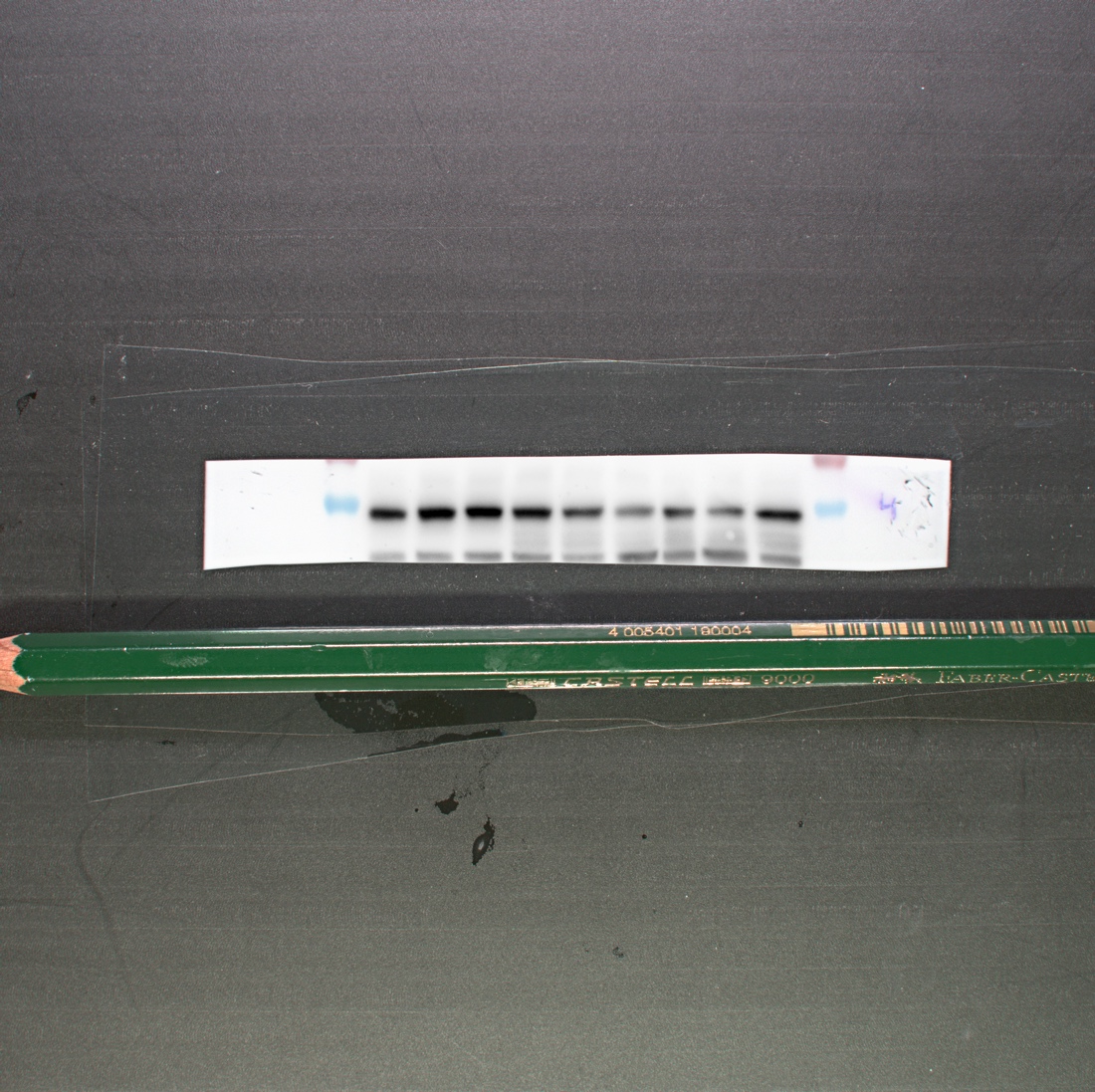

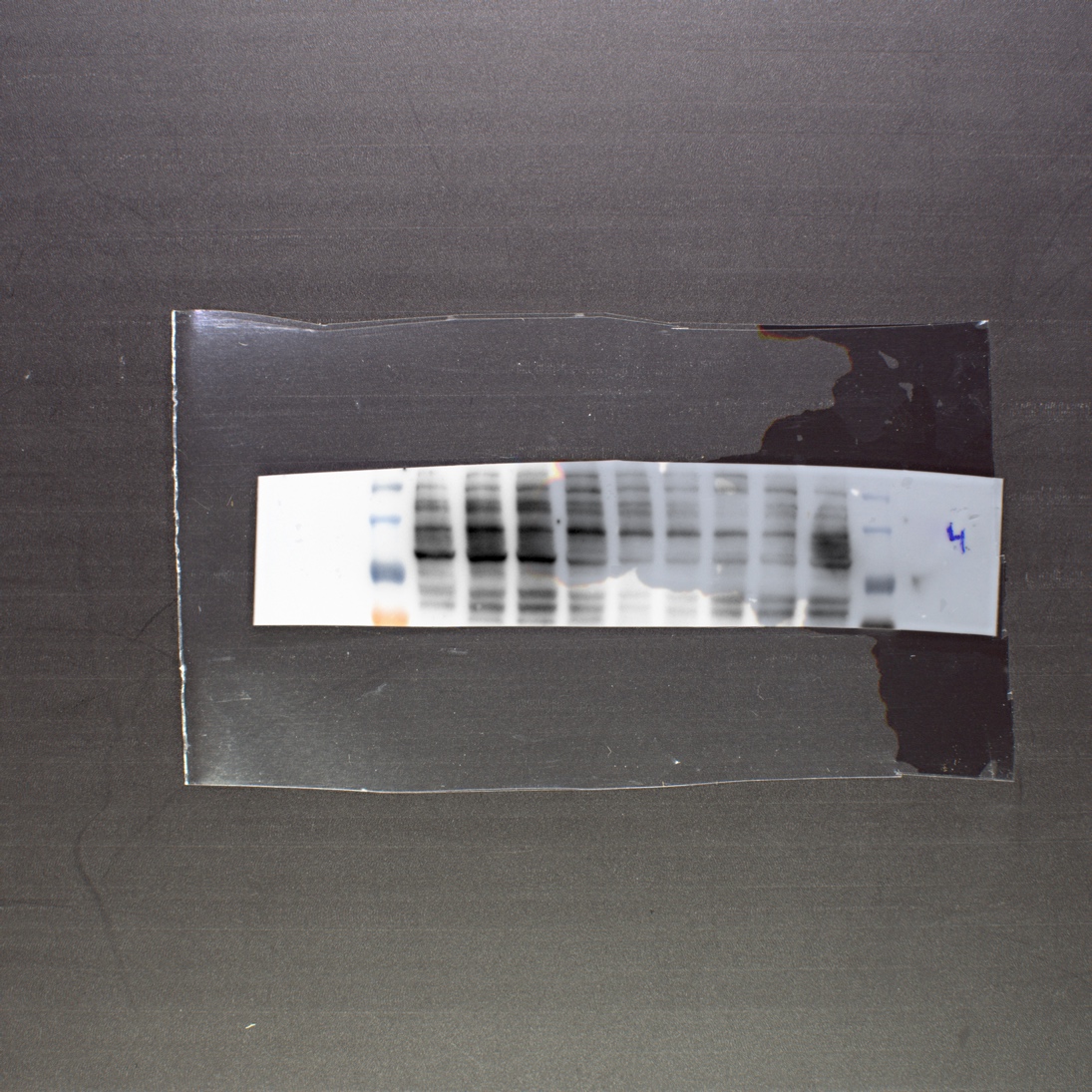
**

RPLP01

Phospho P53

P53

HIF1A

V5 wt 4 hours – phospho p53

**
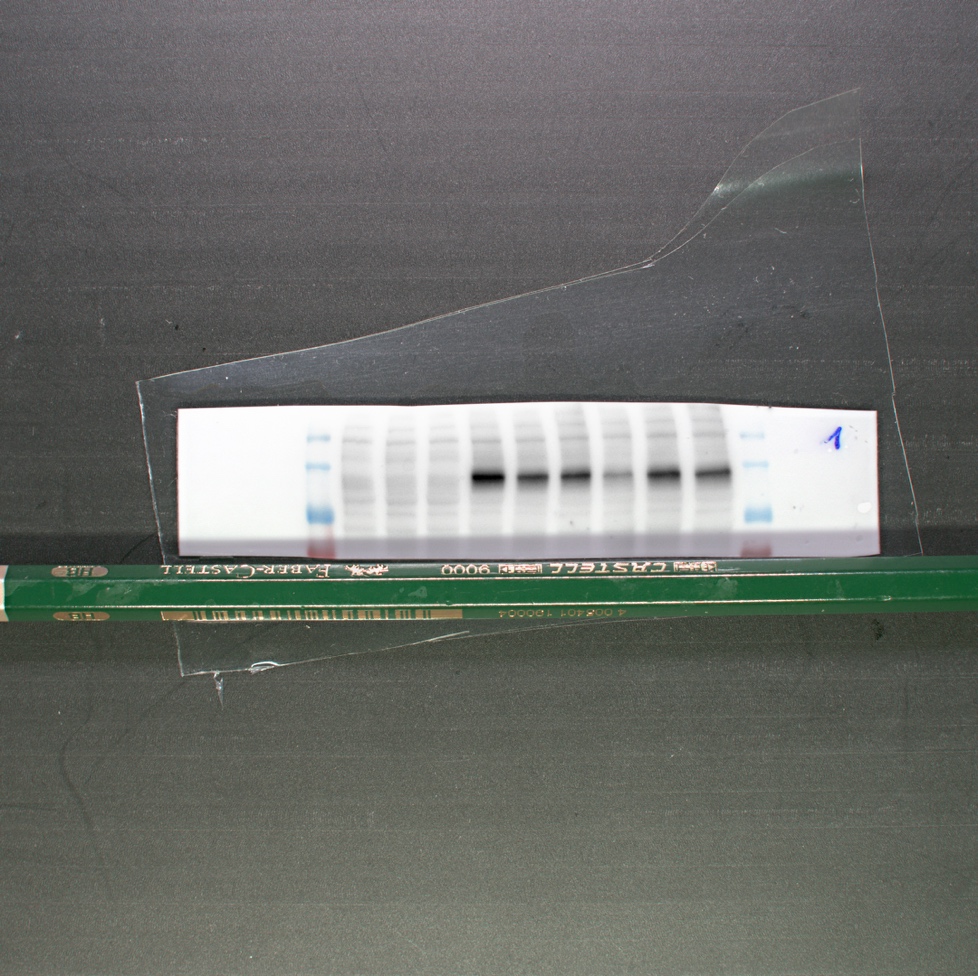
**

HIF1A

Phospho P53

**
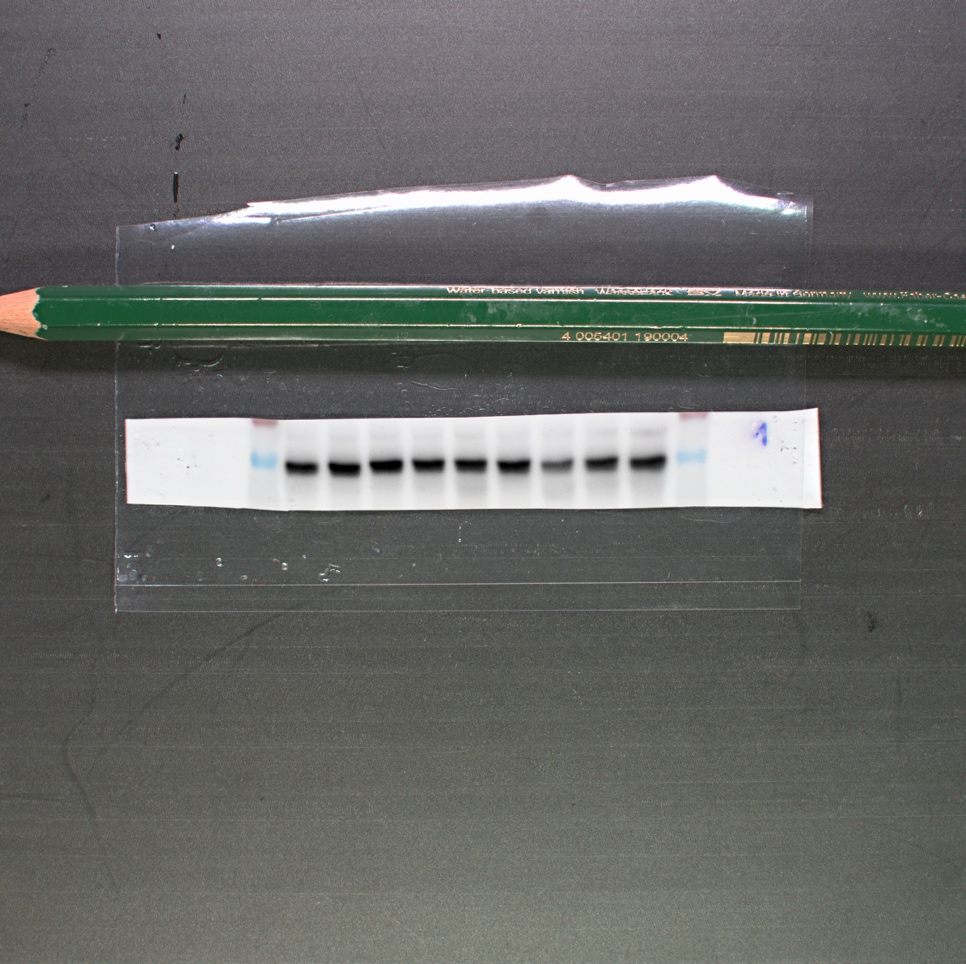

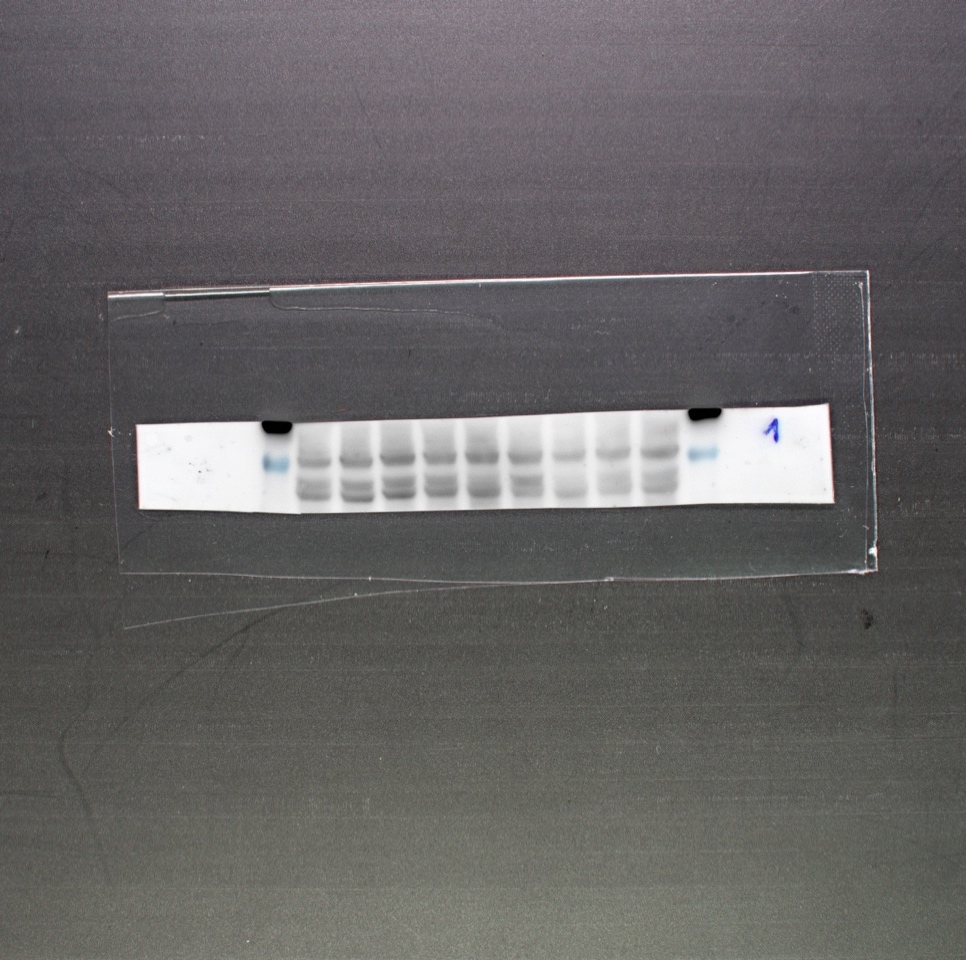
**

P53

RPLP01

**
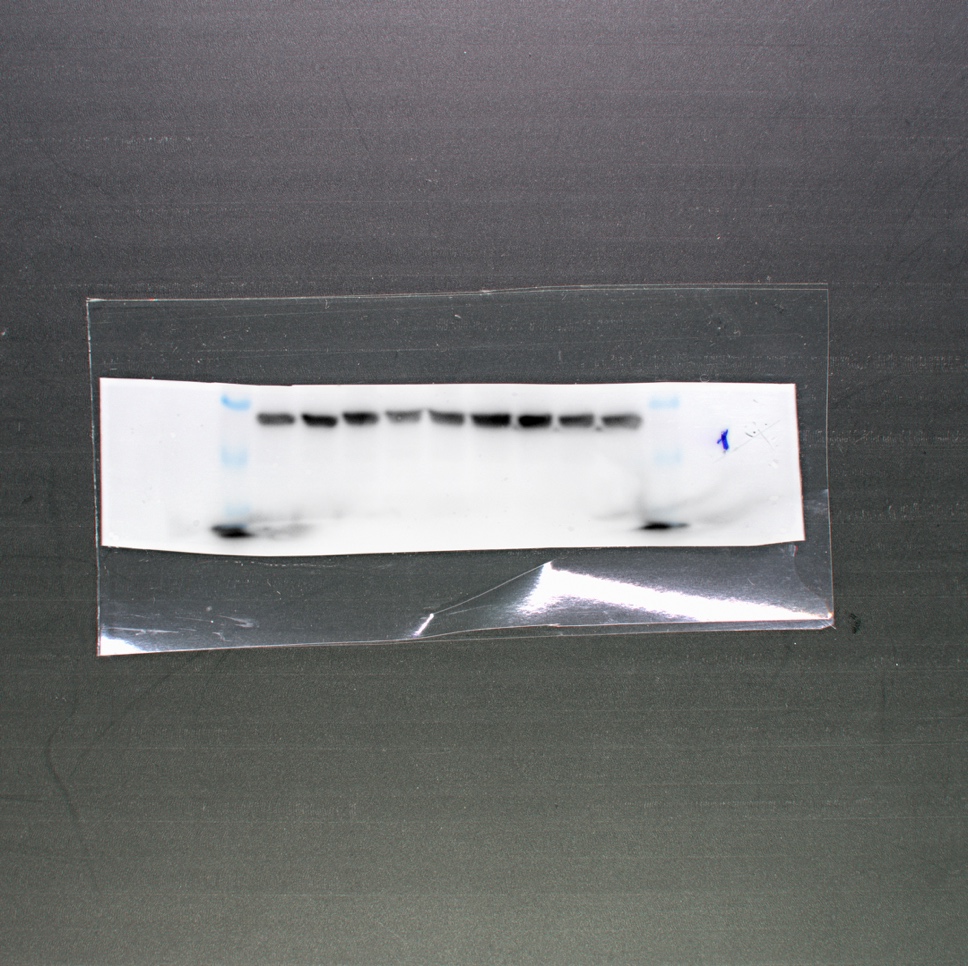
**

V5 wt 24 hours – phopsho p53

**
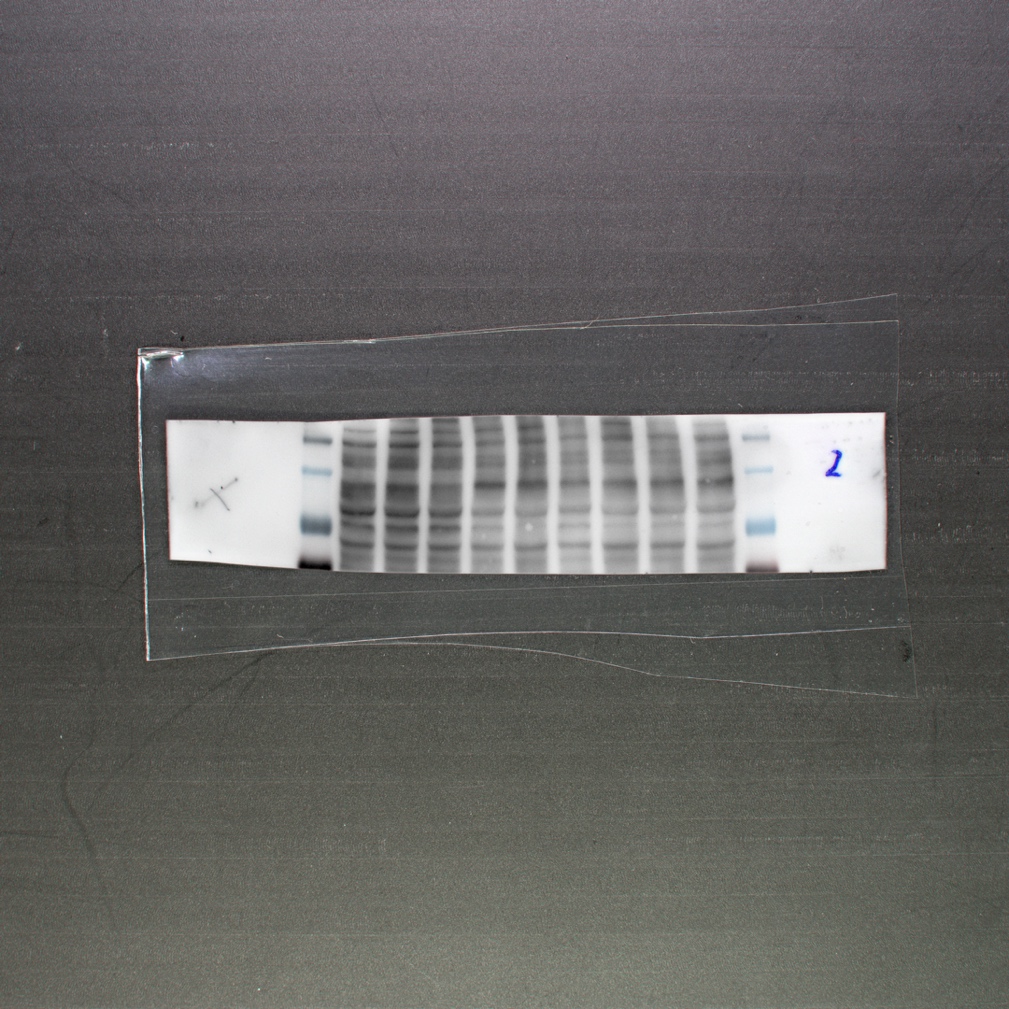

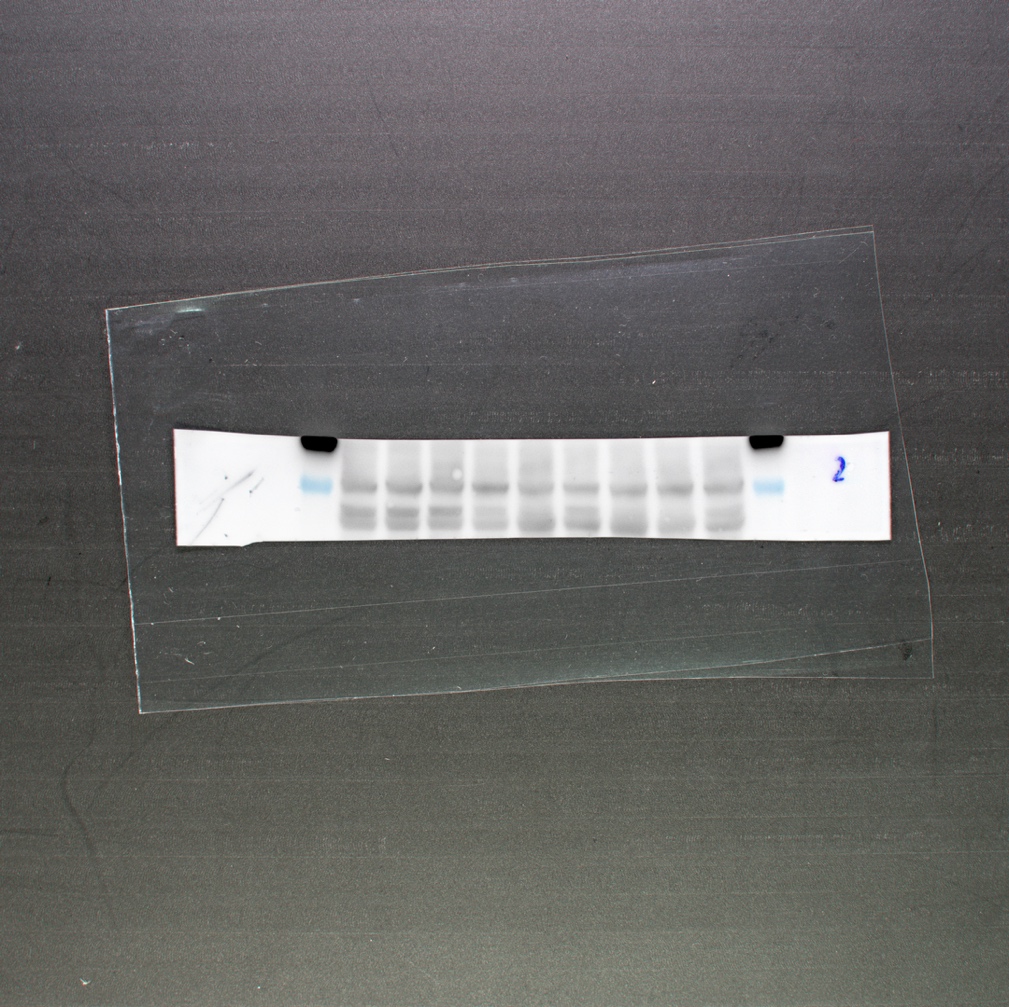

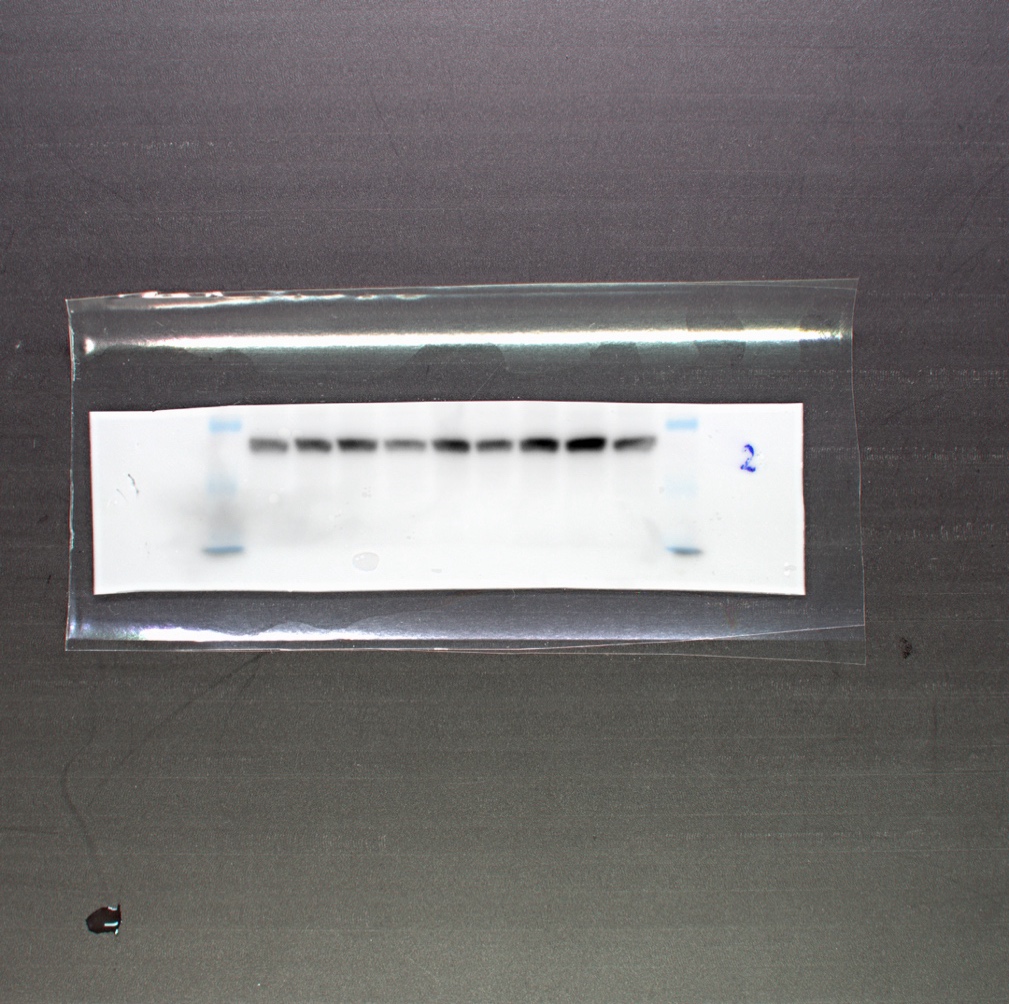
**

HIF1A

Phospho P53

**
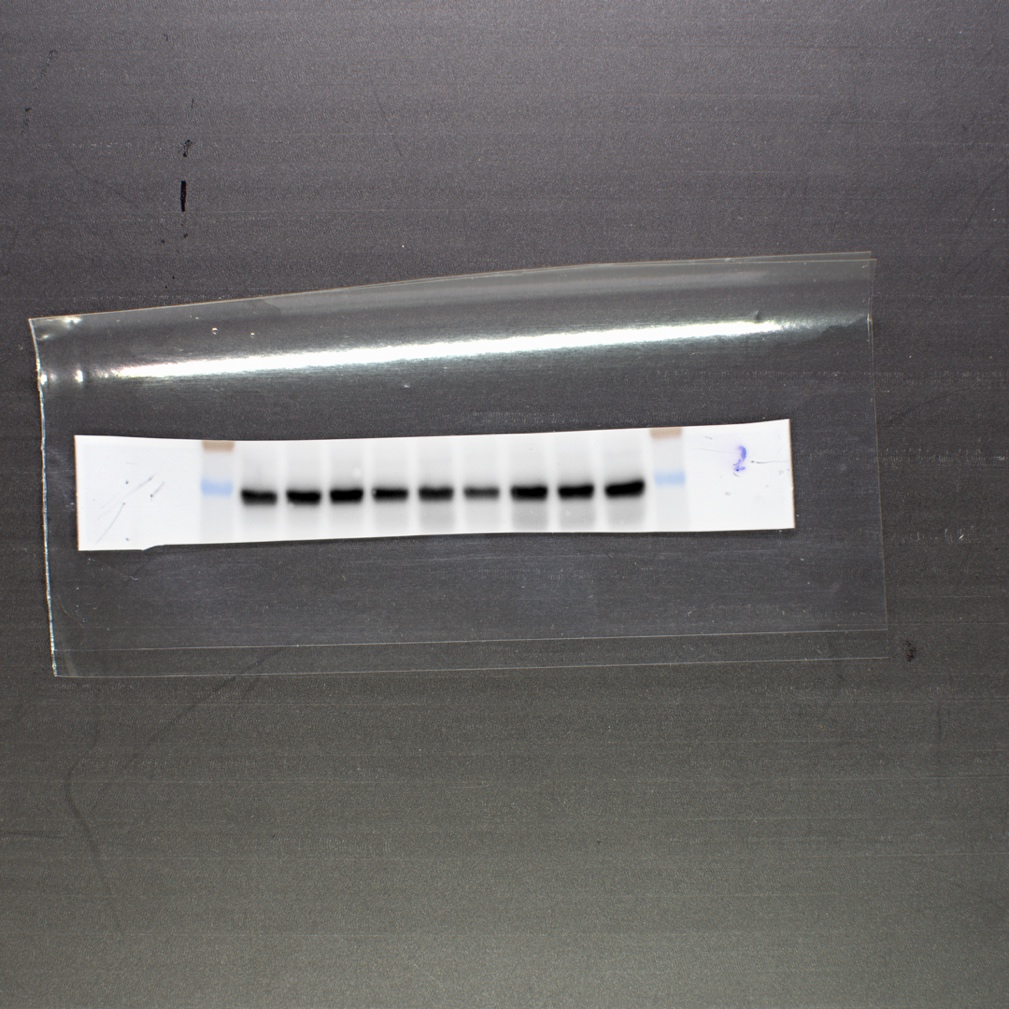
**

P53

RPLP01

V6 wt 4 hours – phospho p53

**
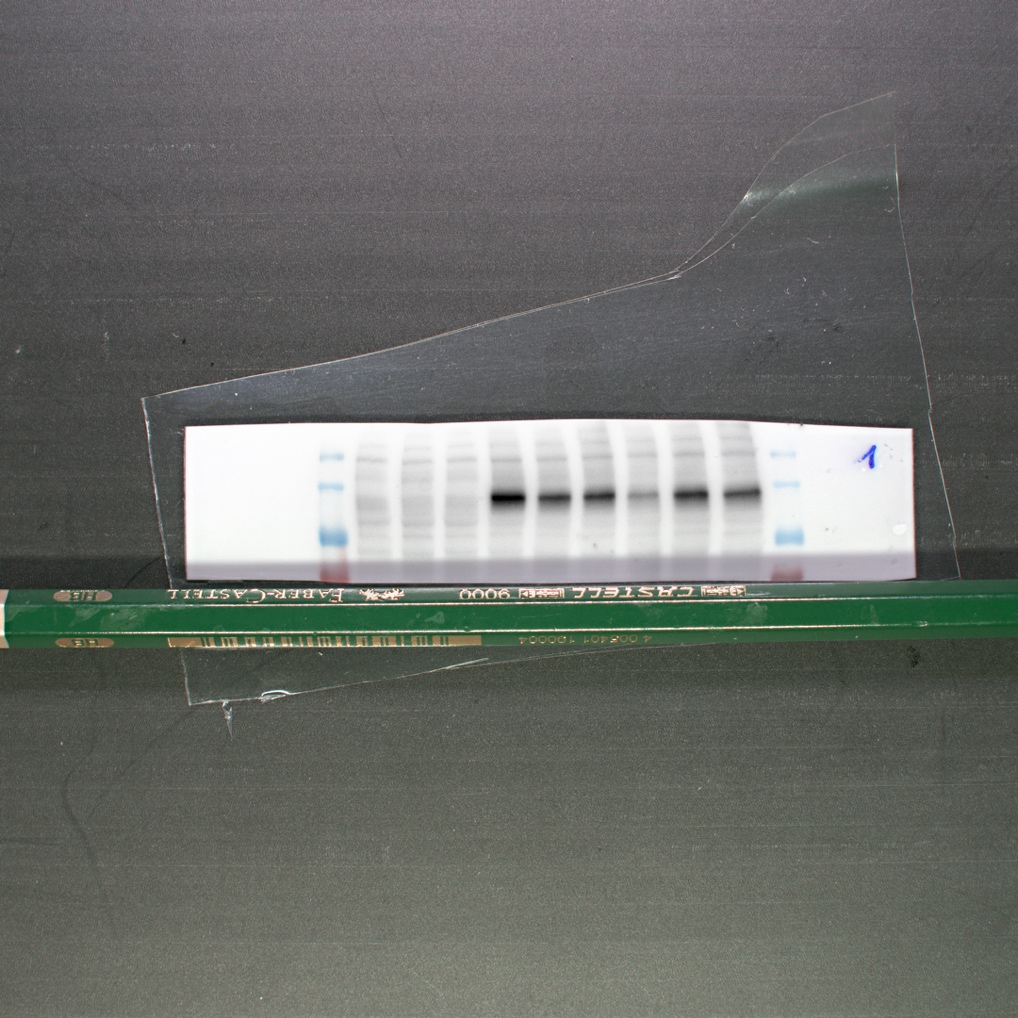
**

HIF1A

Phospho P53

**
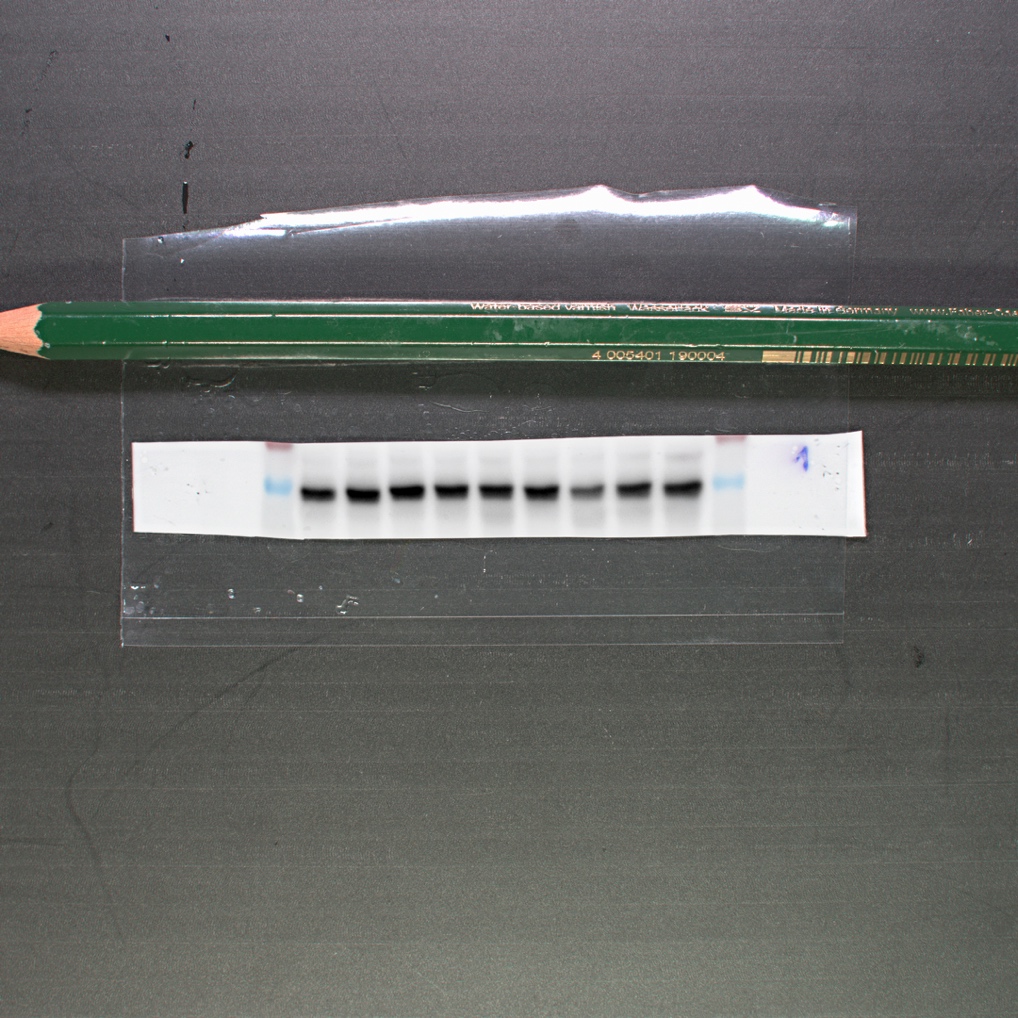

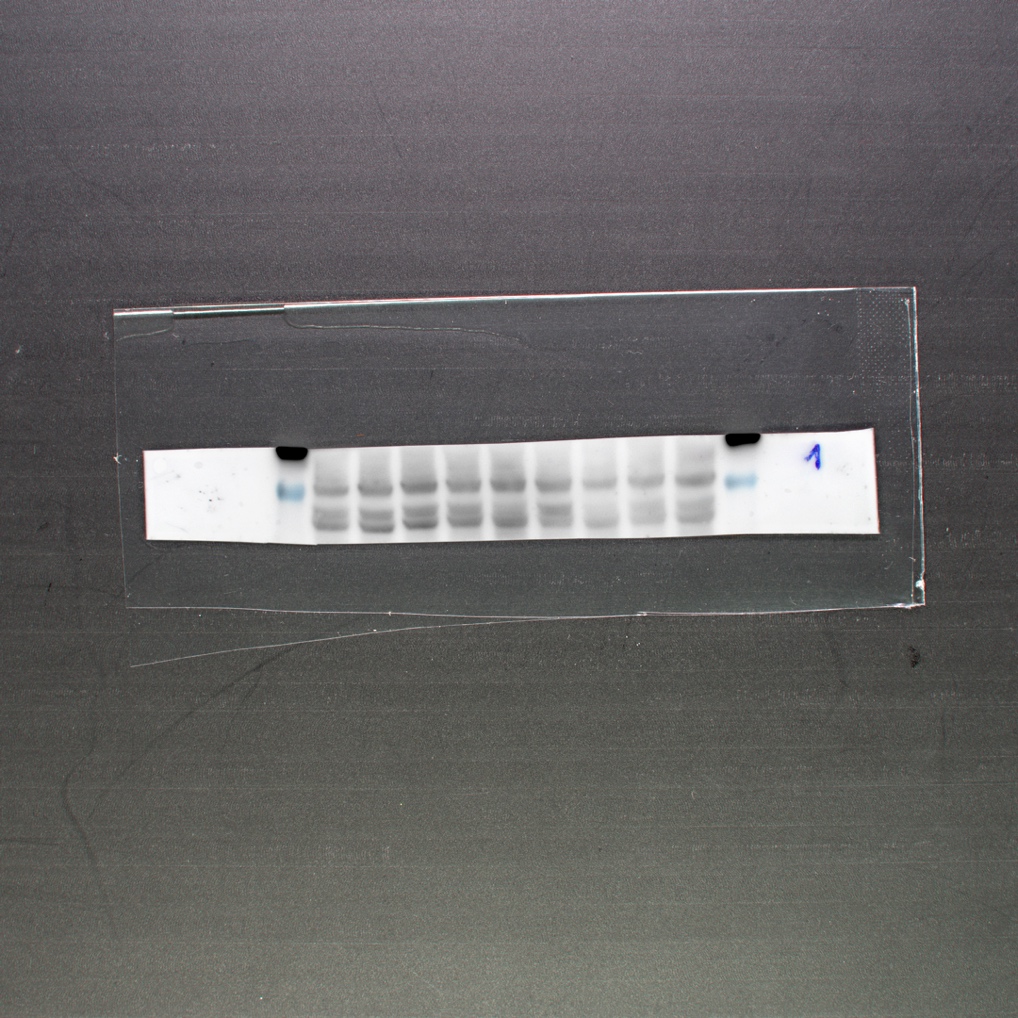
**

P53

RPLP01

**
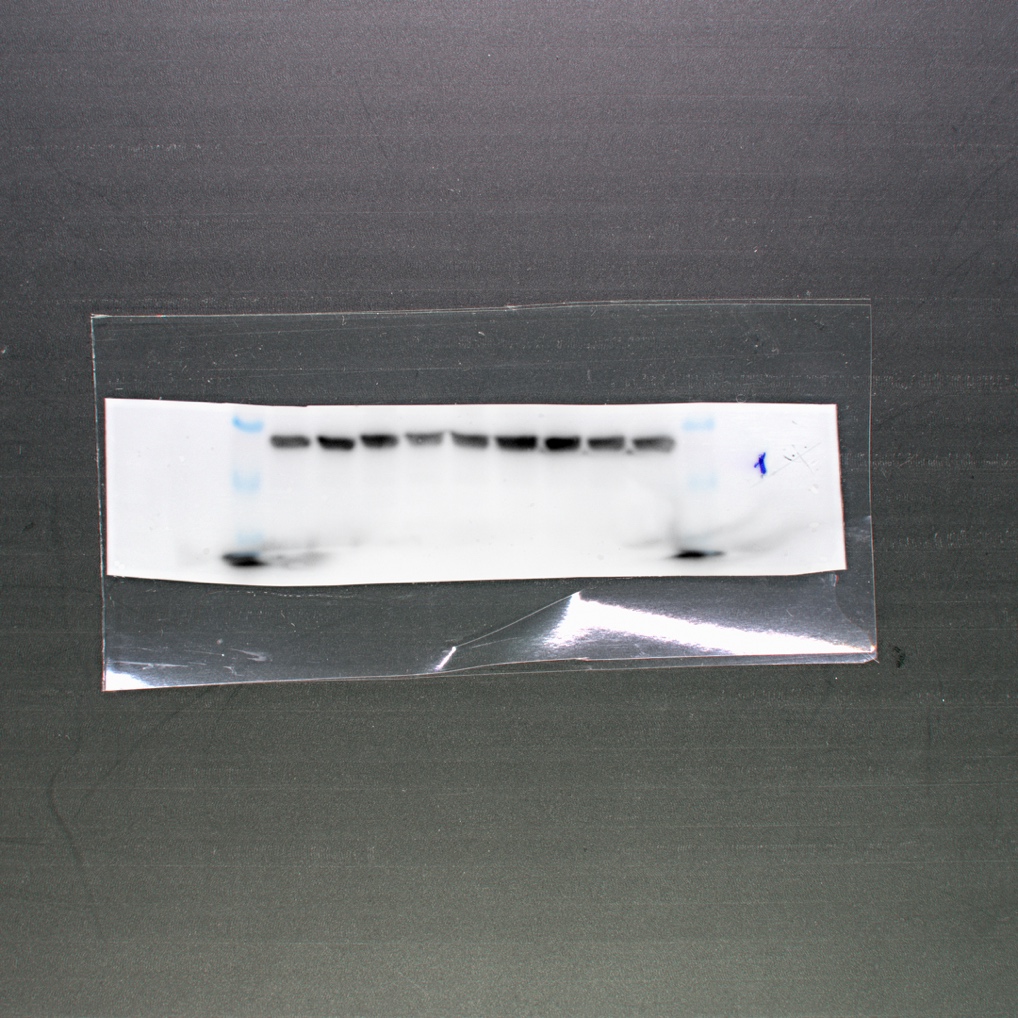
**

V6 wt 24 hours – phospho p53

**
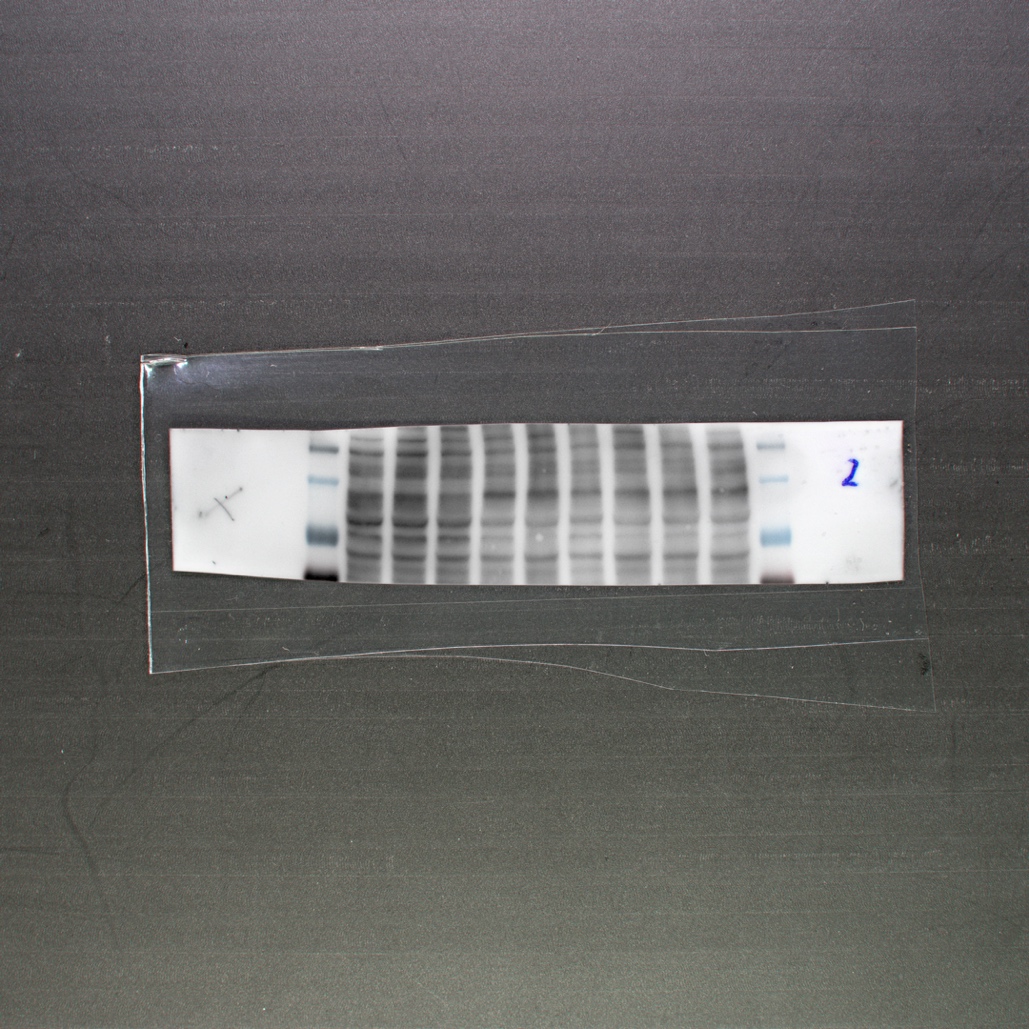
**

HIF1A

**
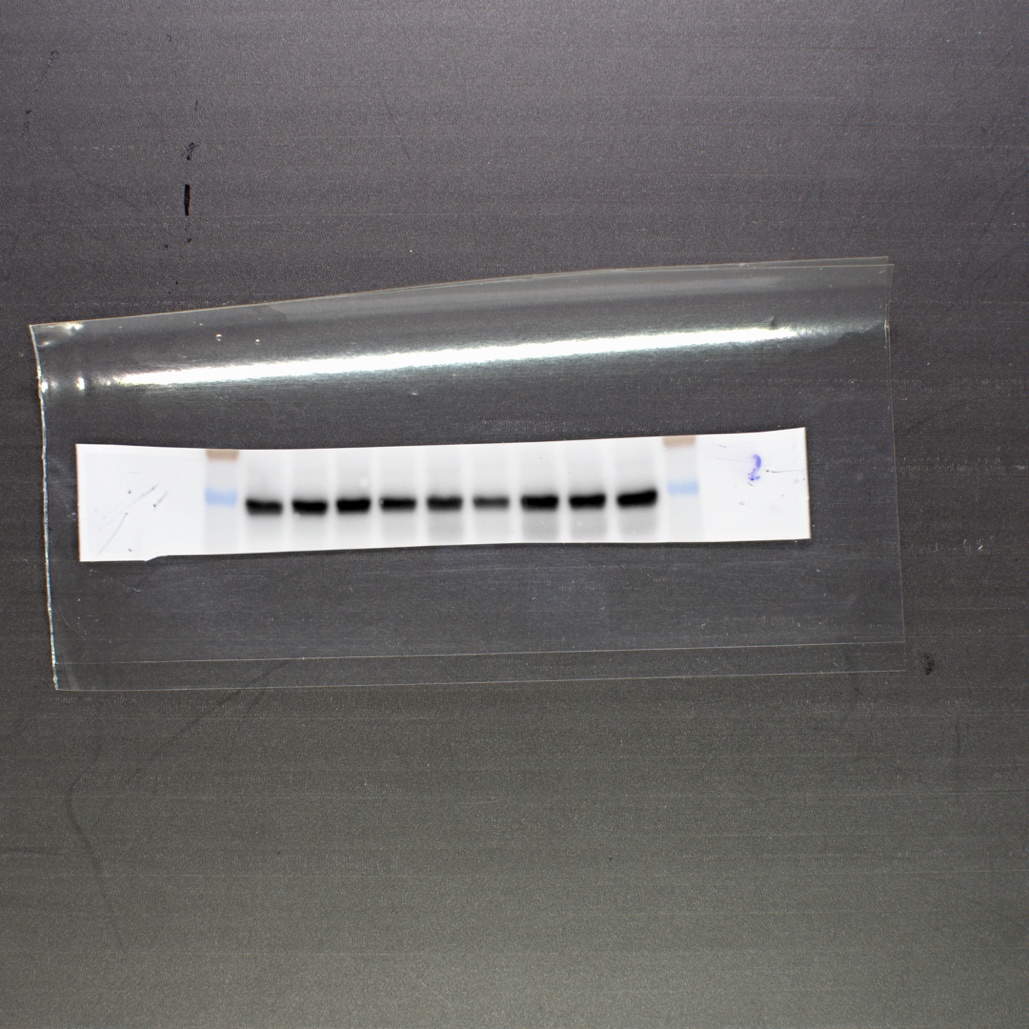

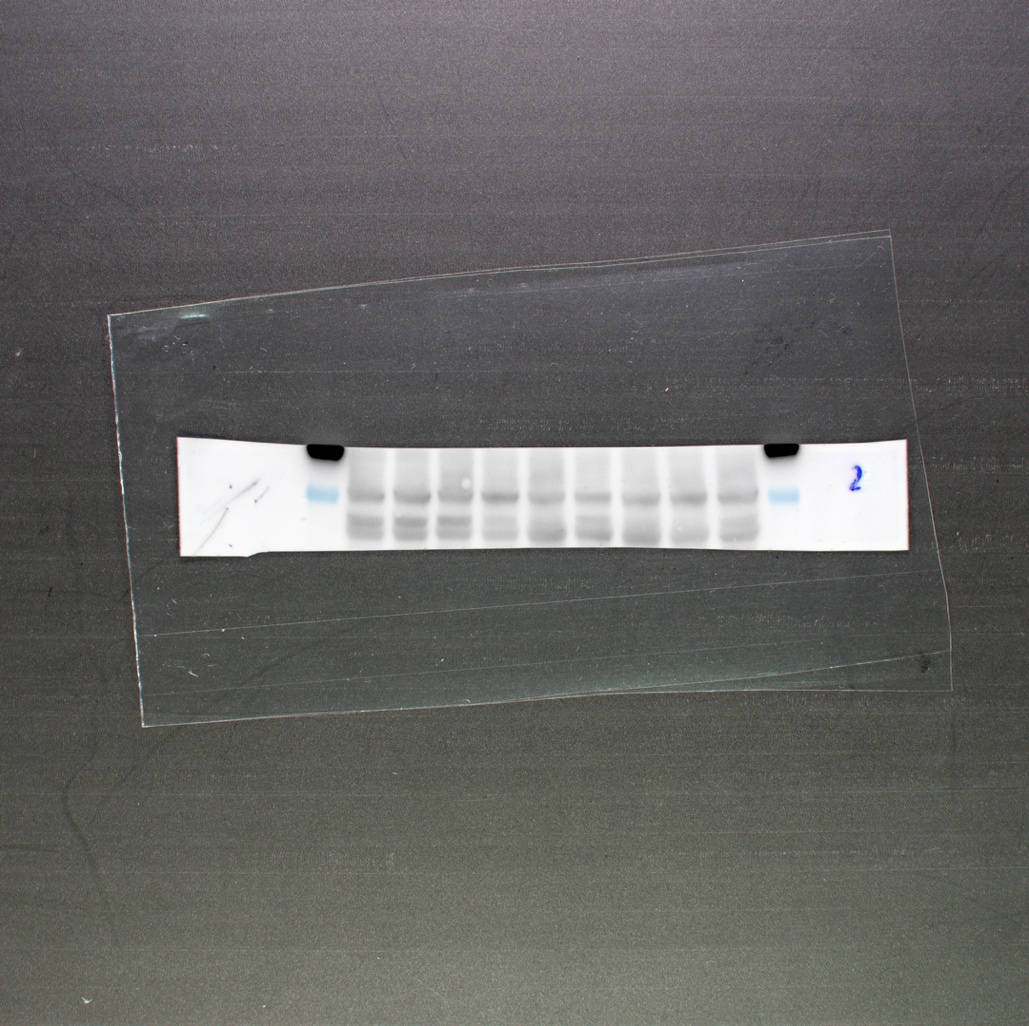
**

Phospho P53

P53

**
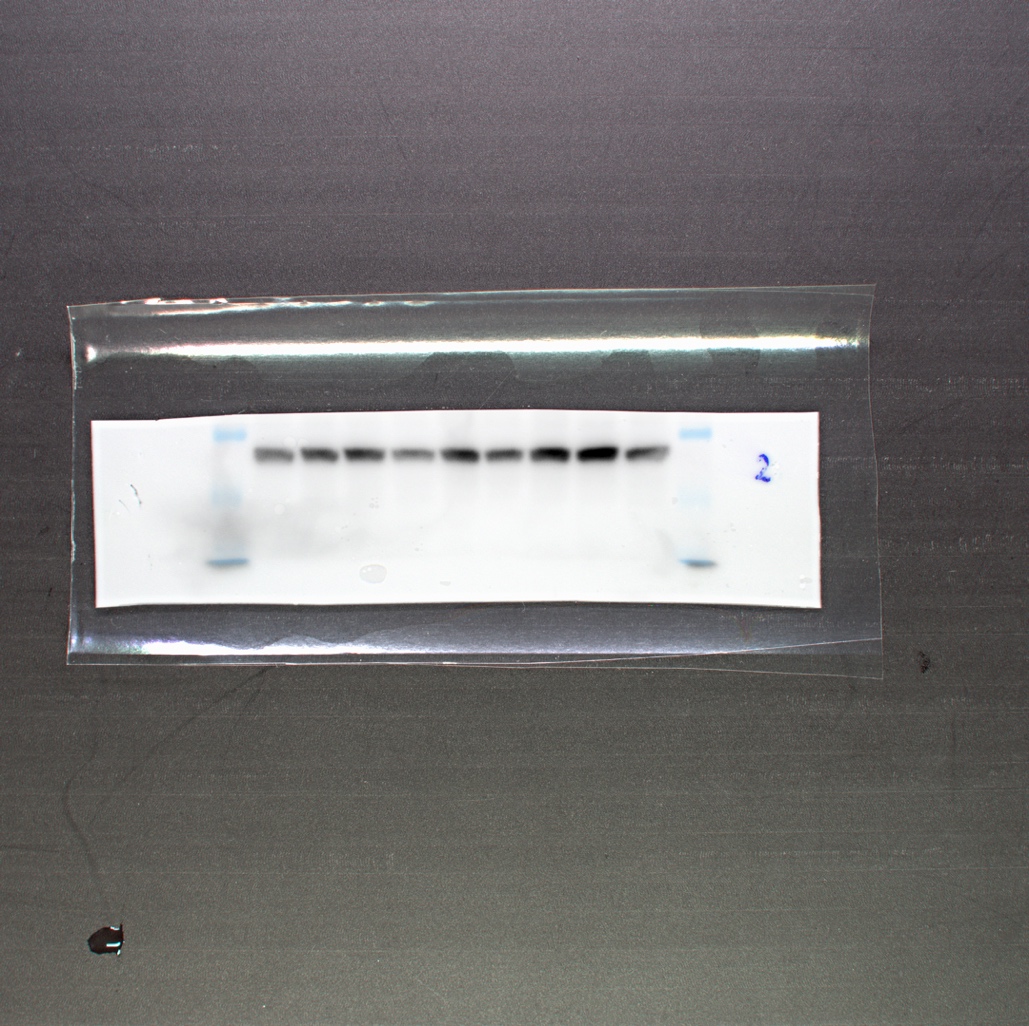
**

RPLP01

V7 wt 24 hours – phosphp P53 (Example Blot in Fig. 1C)

**
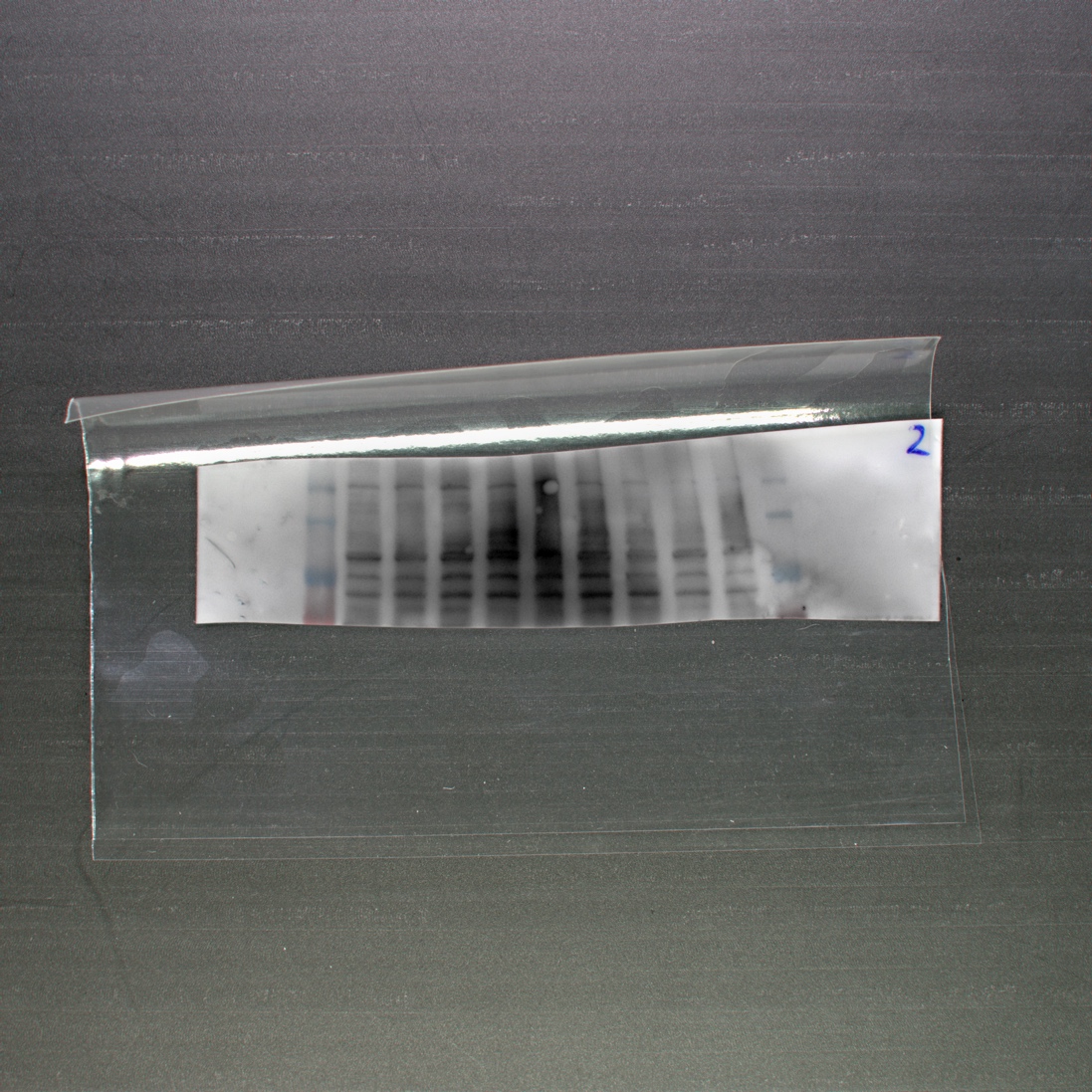
**

HIF1A

Phospho P53

**
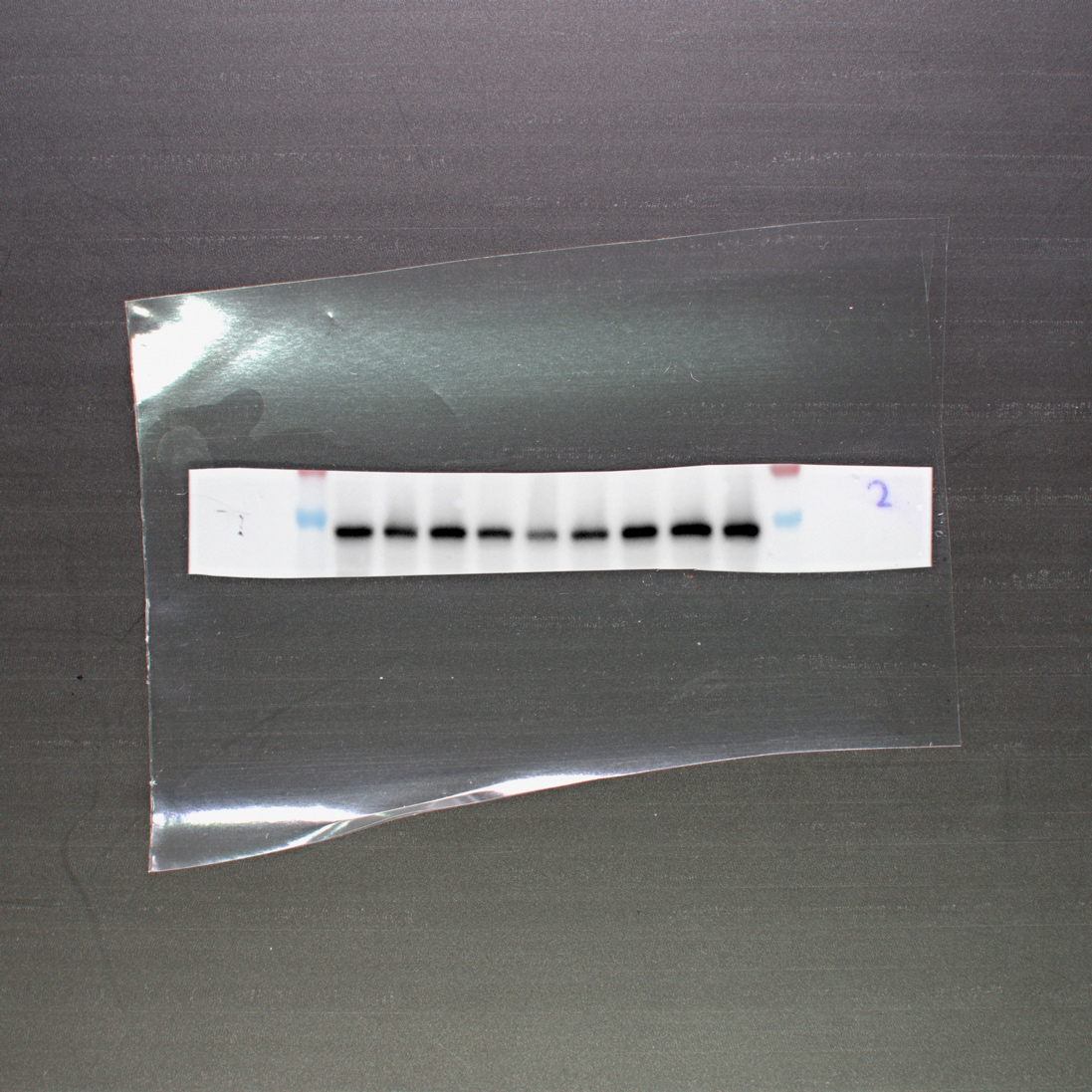

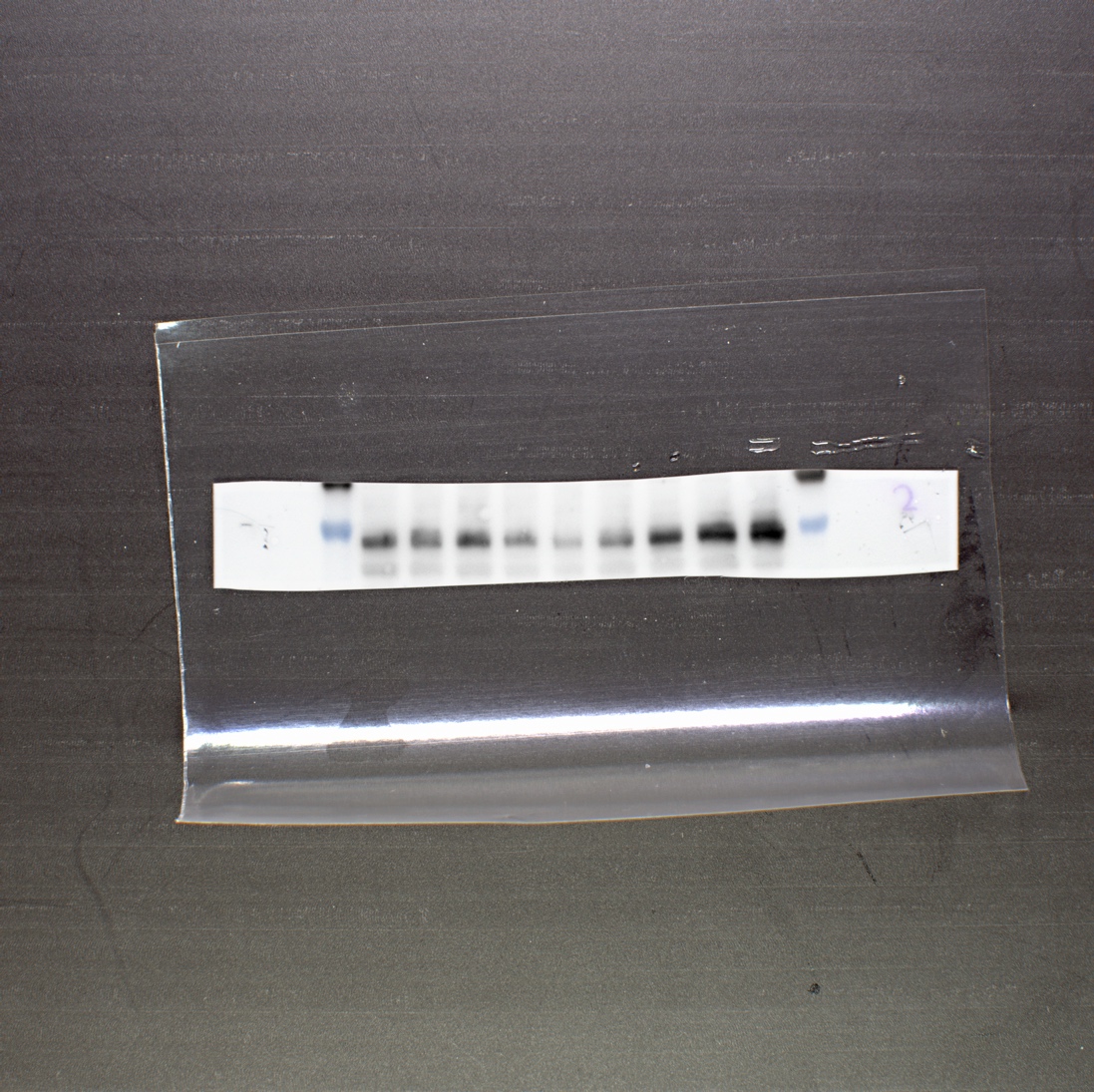
**

P53

**
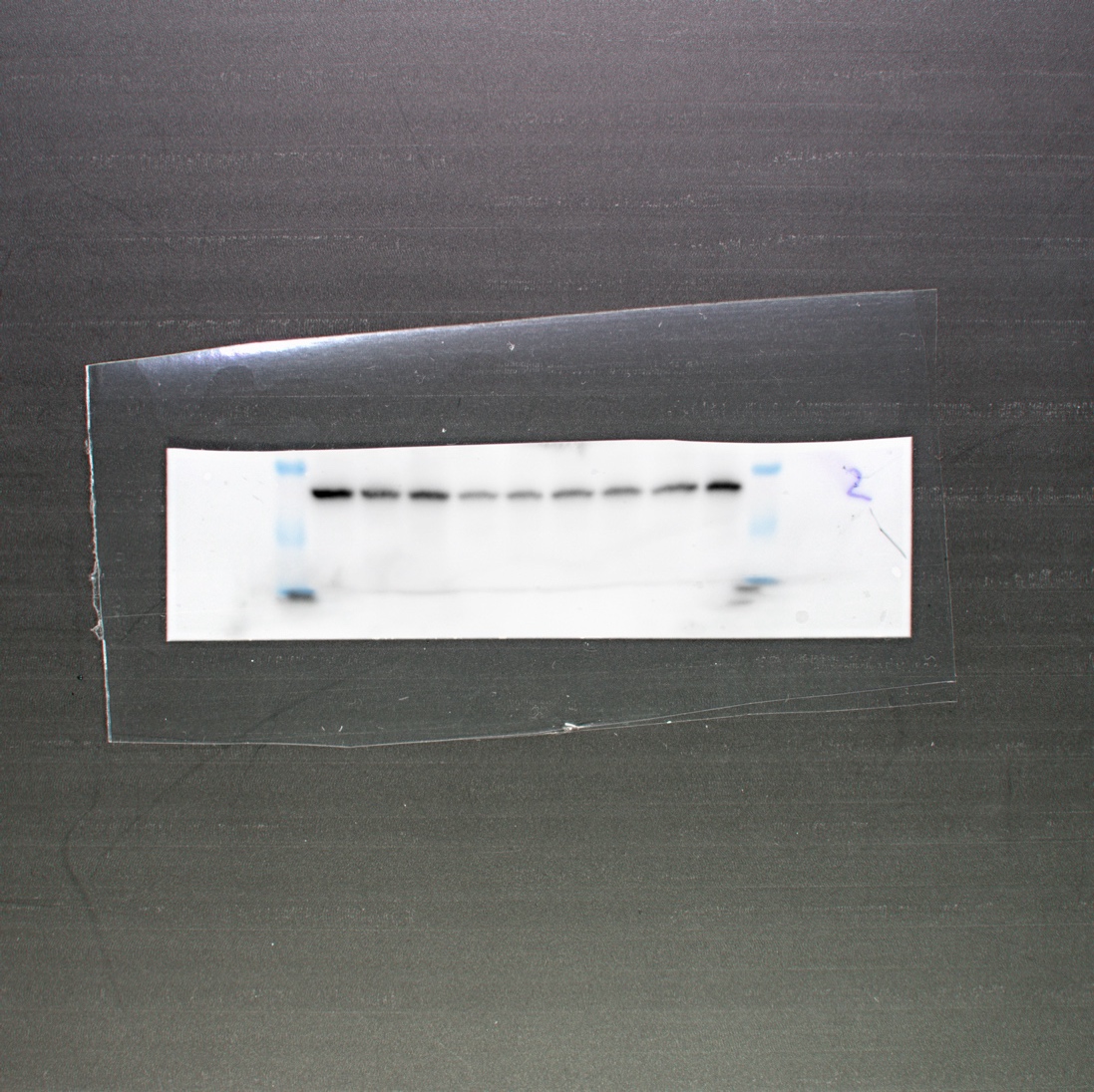
**

RPLP01

# Western Blots for Figure 3 B/F

V1 p53 ko 4 hours

**
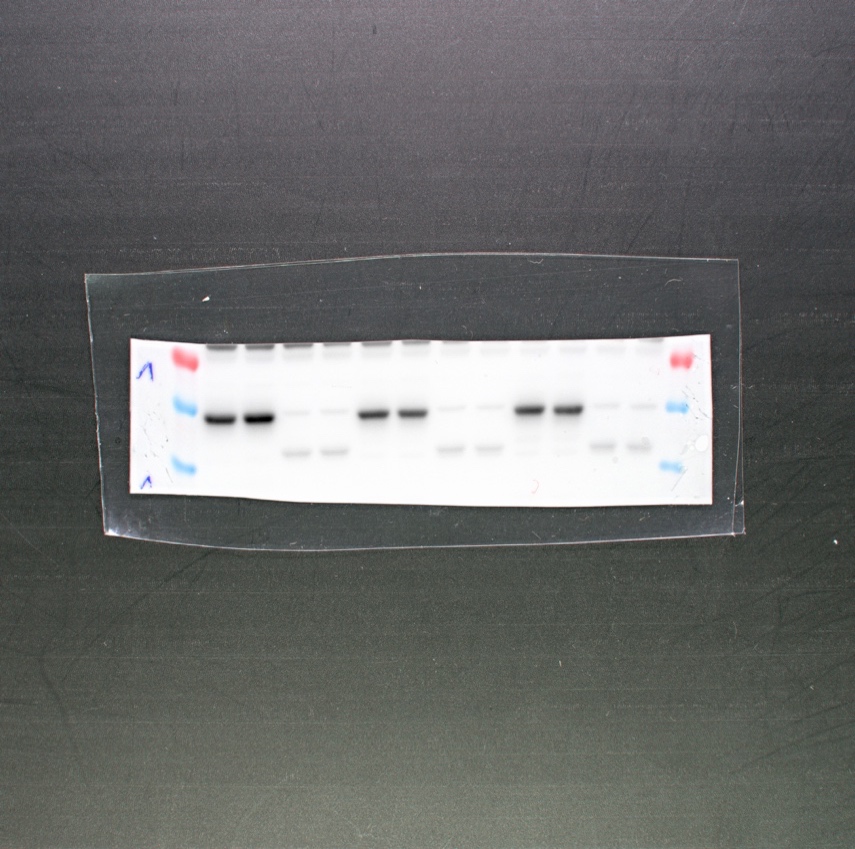

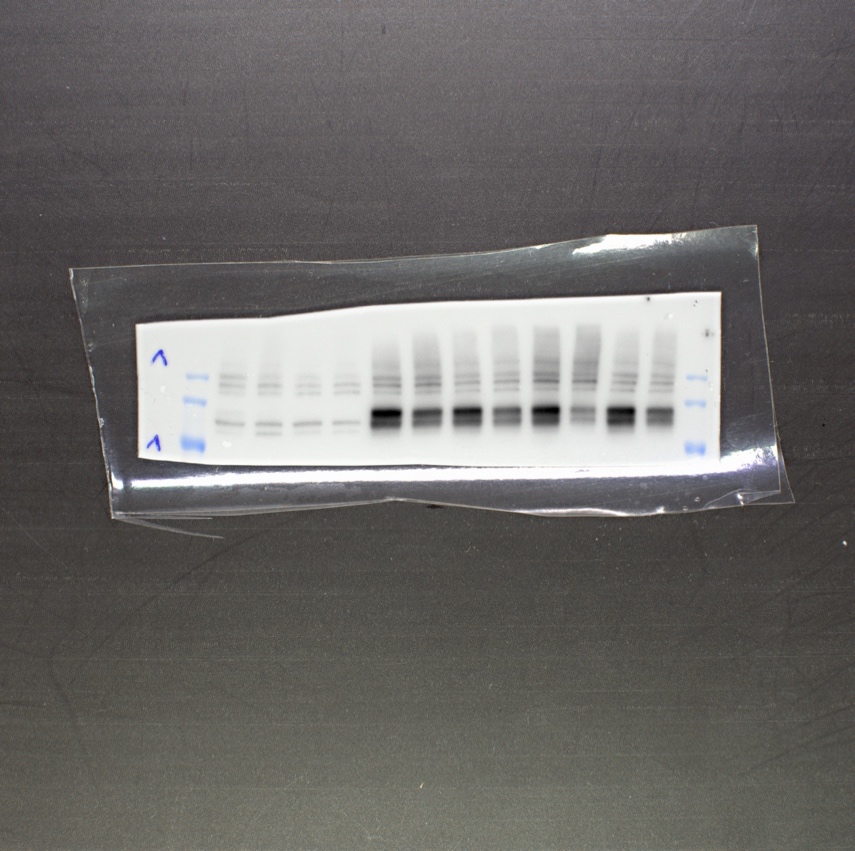

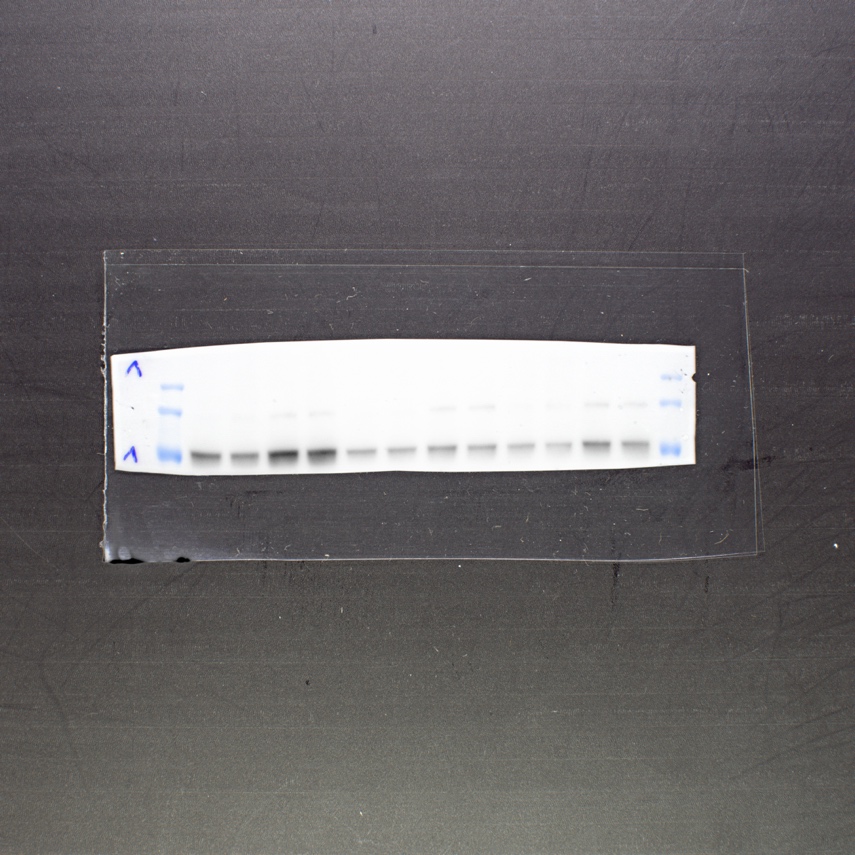
**

P53

ARNT

HIF1A

**
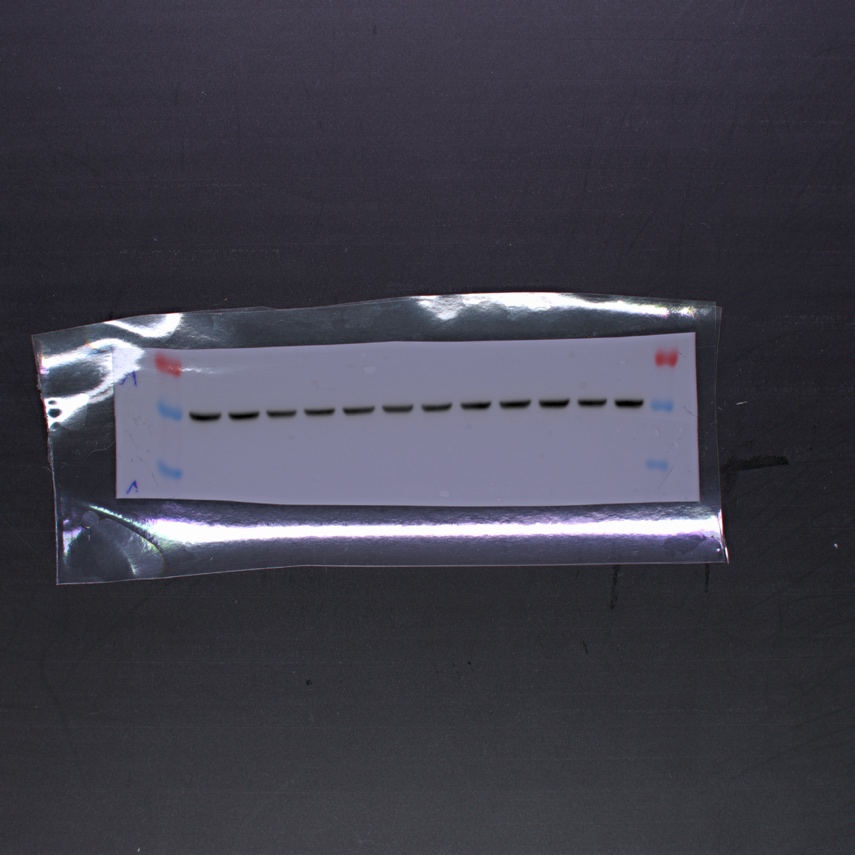
**

α-Tubulin

**
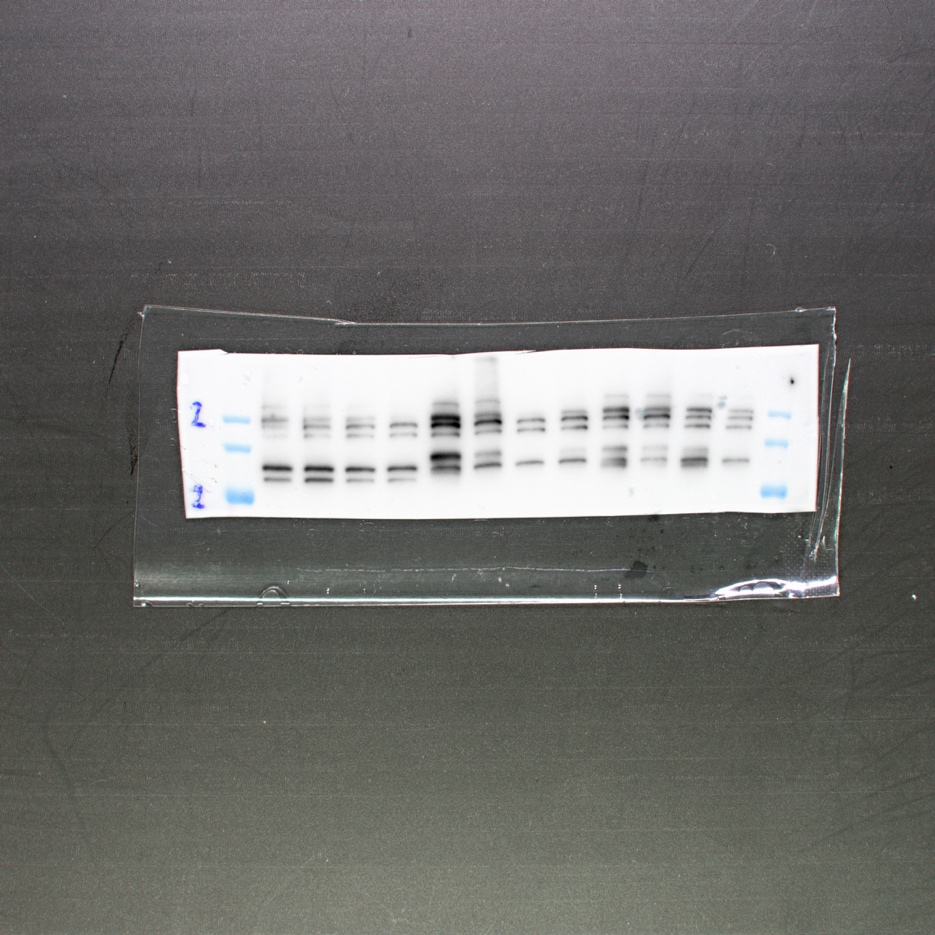

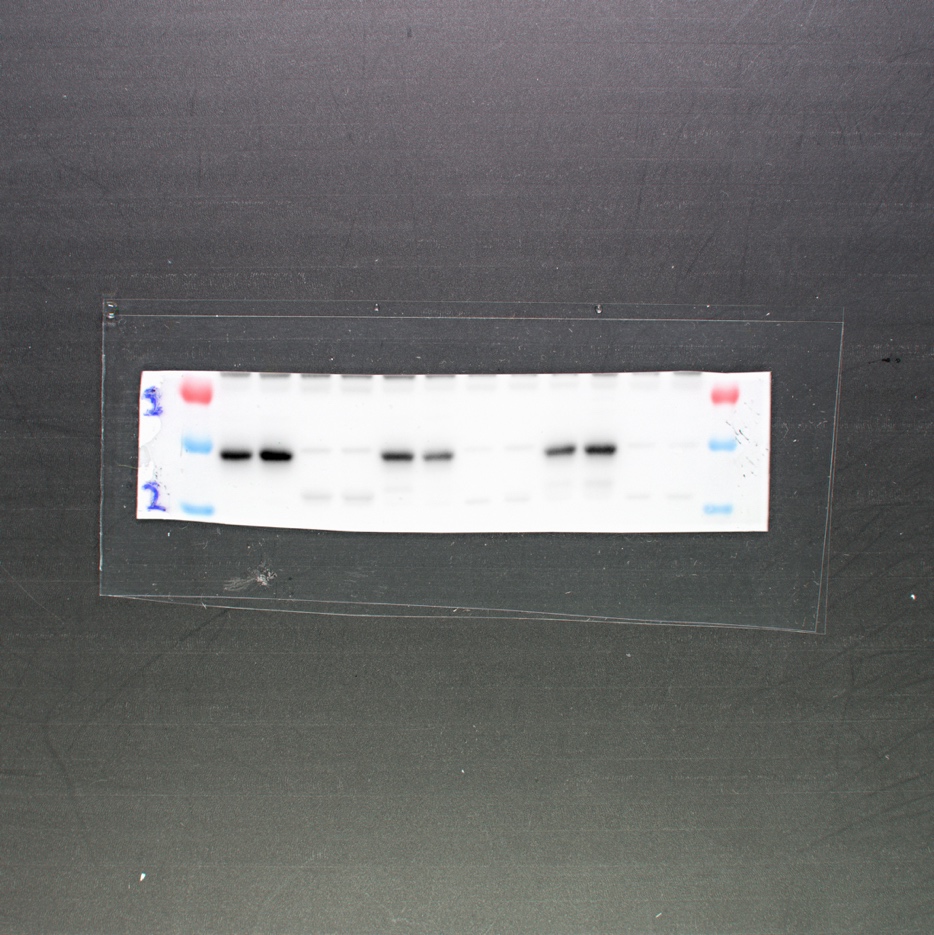

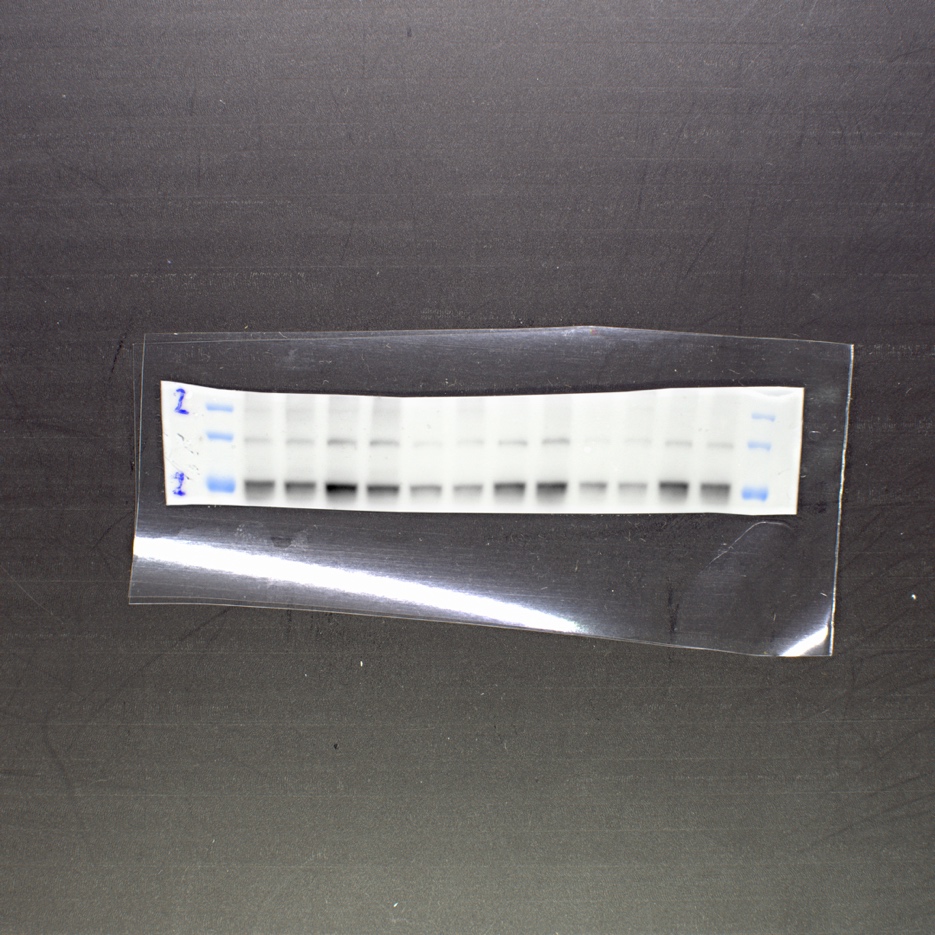
**

V1 p53 ko 24 hours

HIF1A

ARNT

**
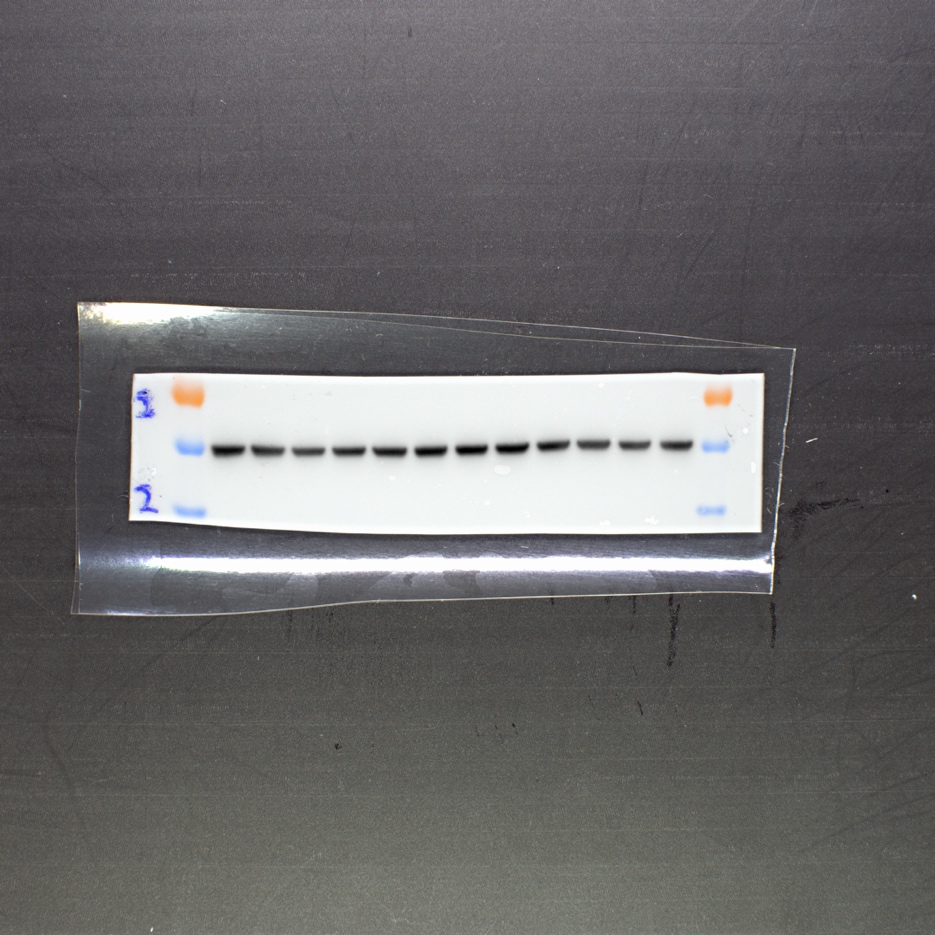
**

α-Tubulin

P53

V2 p53 ko 4 hours (Example Blot in Fig. 3B)

**
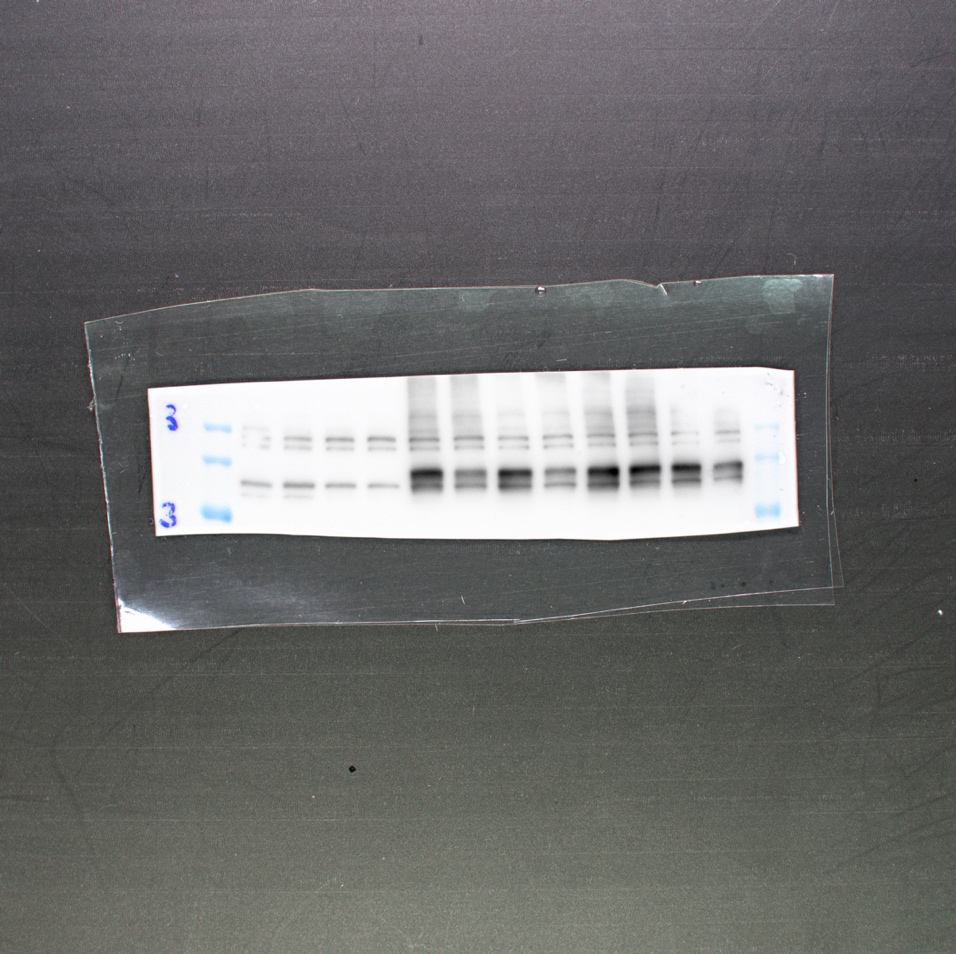
**

HIF1A

**
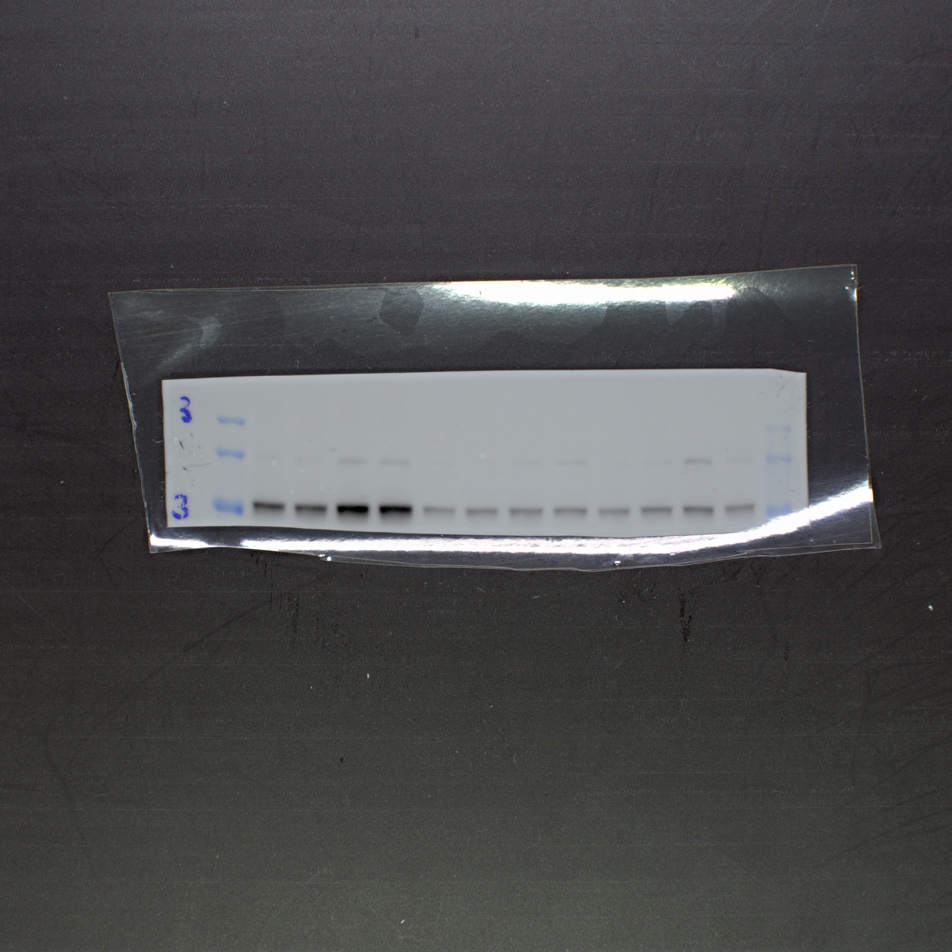
**

ARNT

**
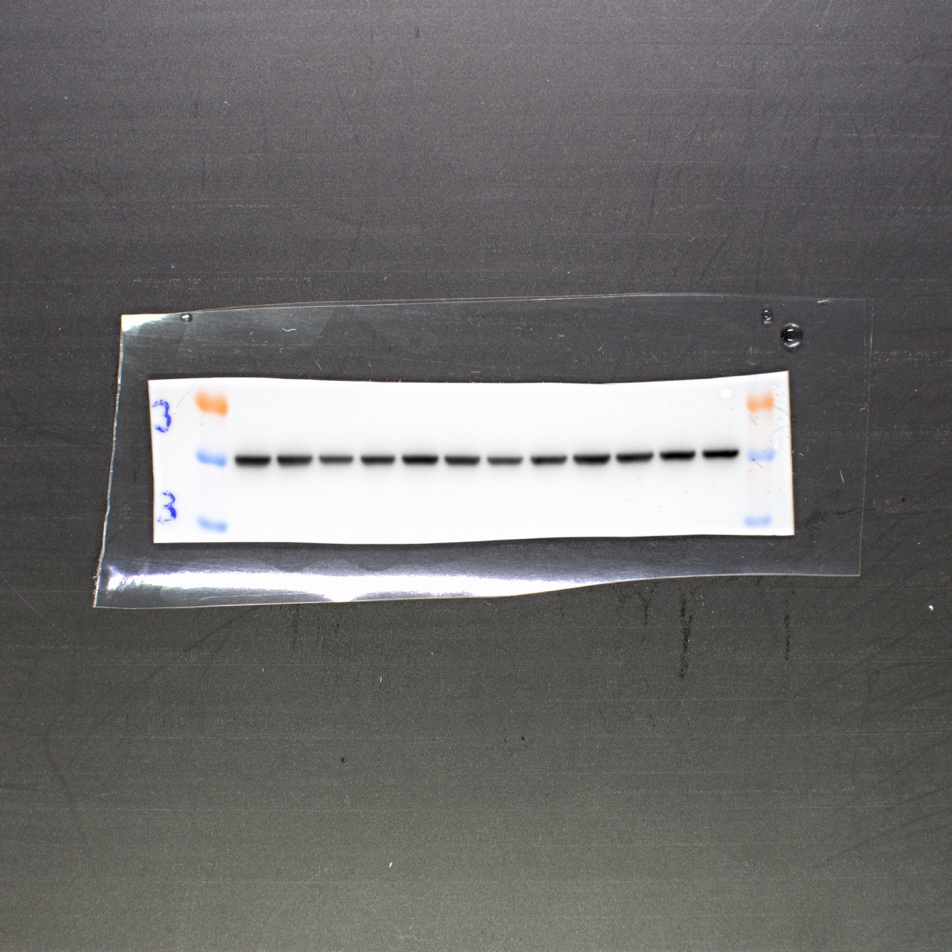
**

α-Tubulin

**
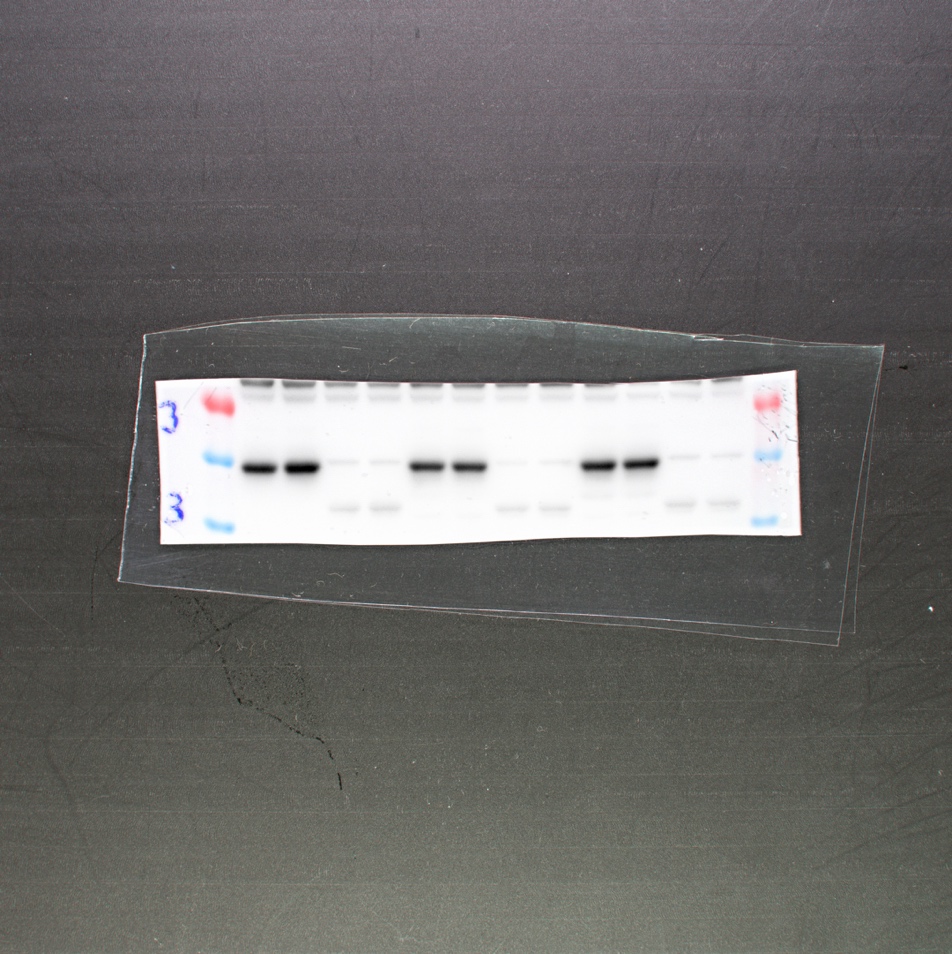
**

P53

**
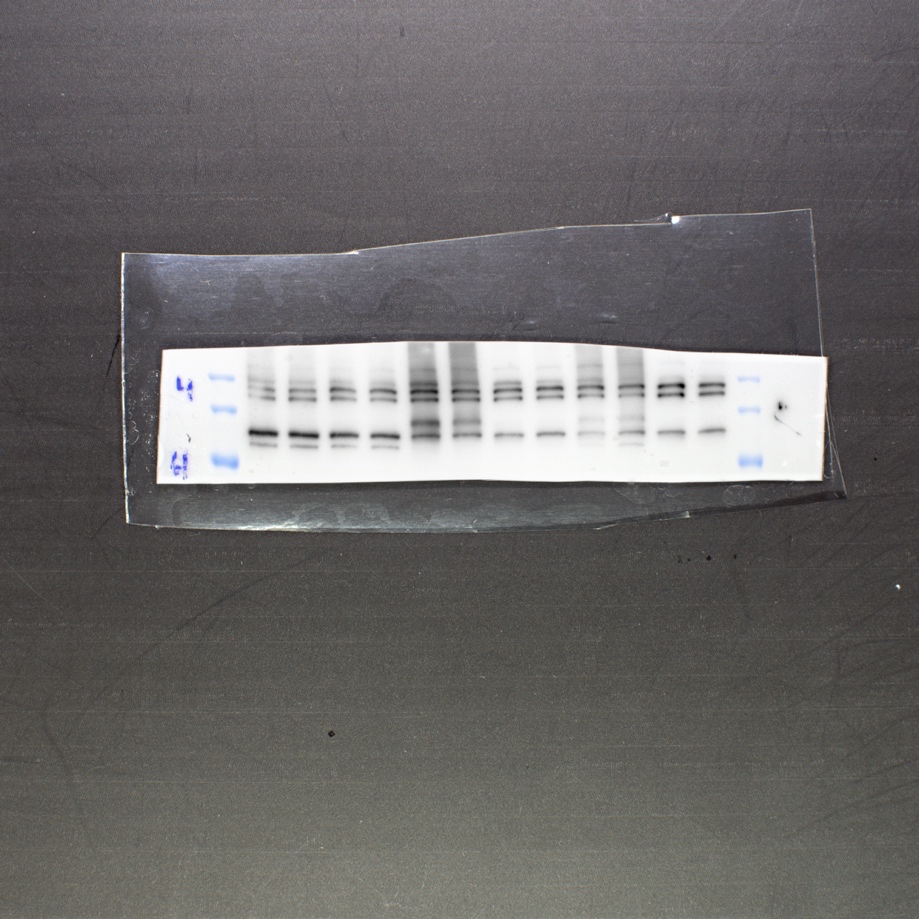

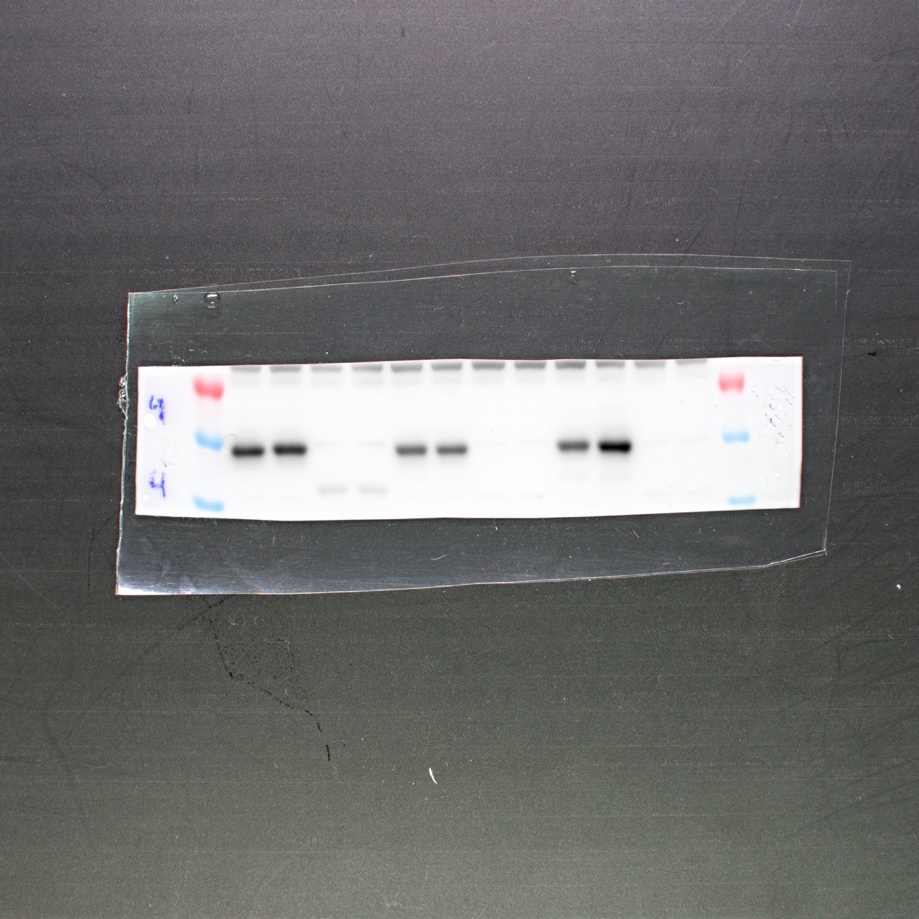

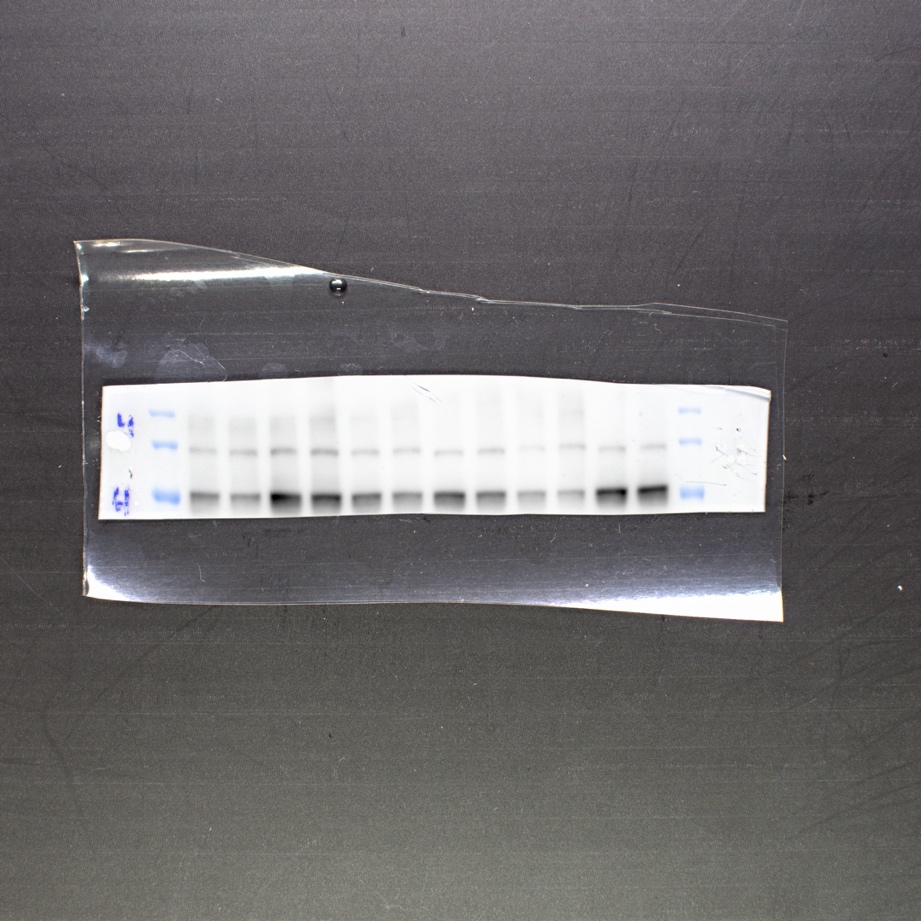
**

V2 p53 ko 24 hours

ARNT

P53

HIF1A

**
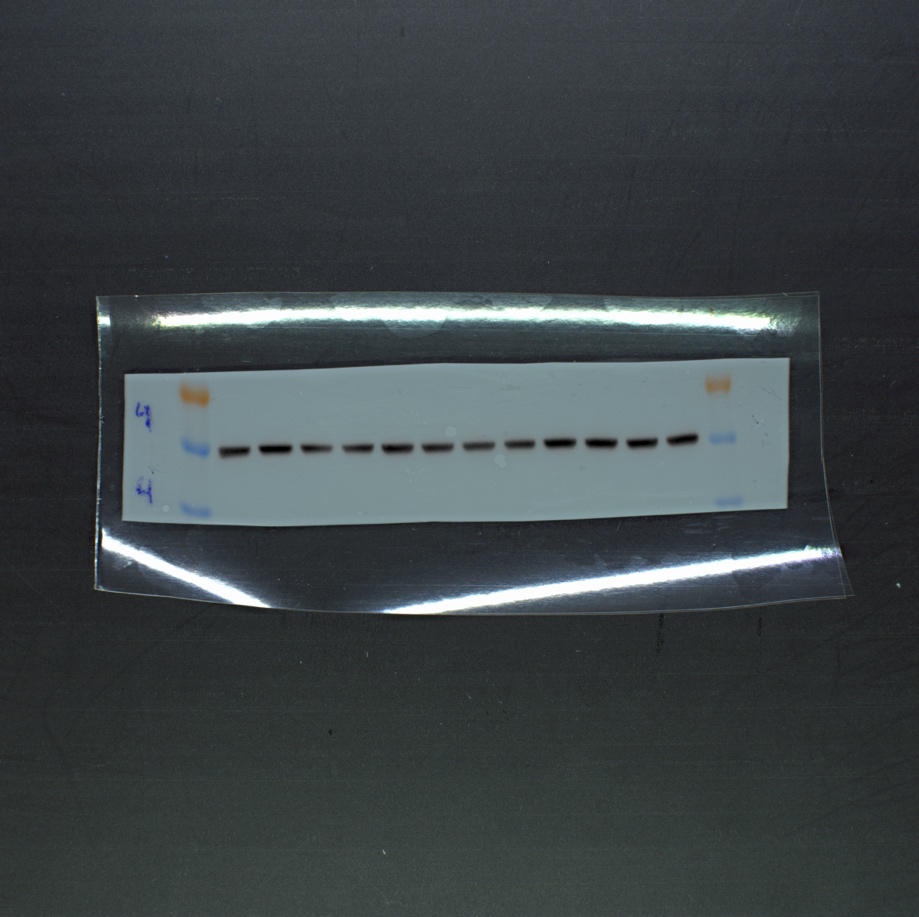
**

α-Tubulin

V3 p53 ko 4 hours

**
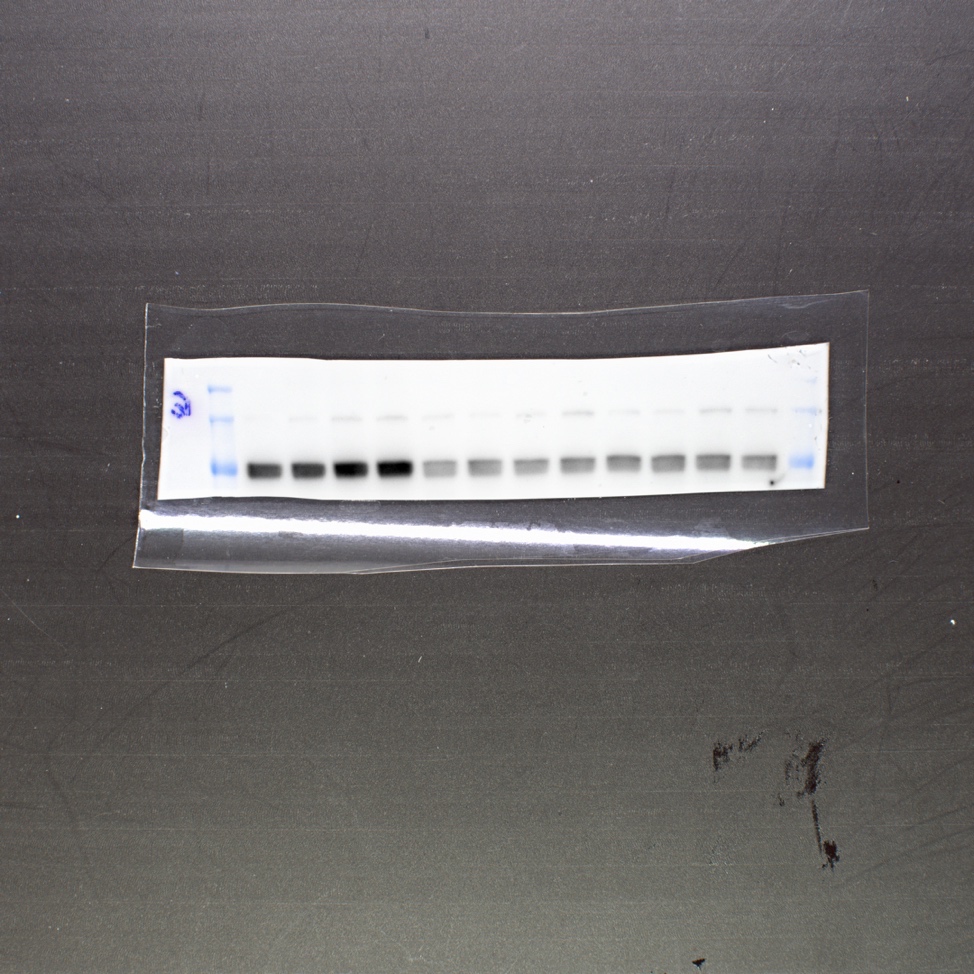

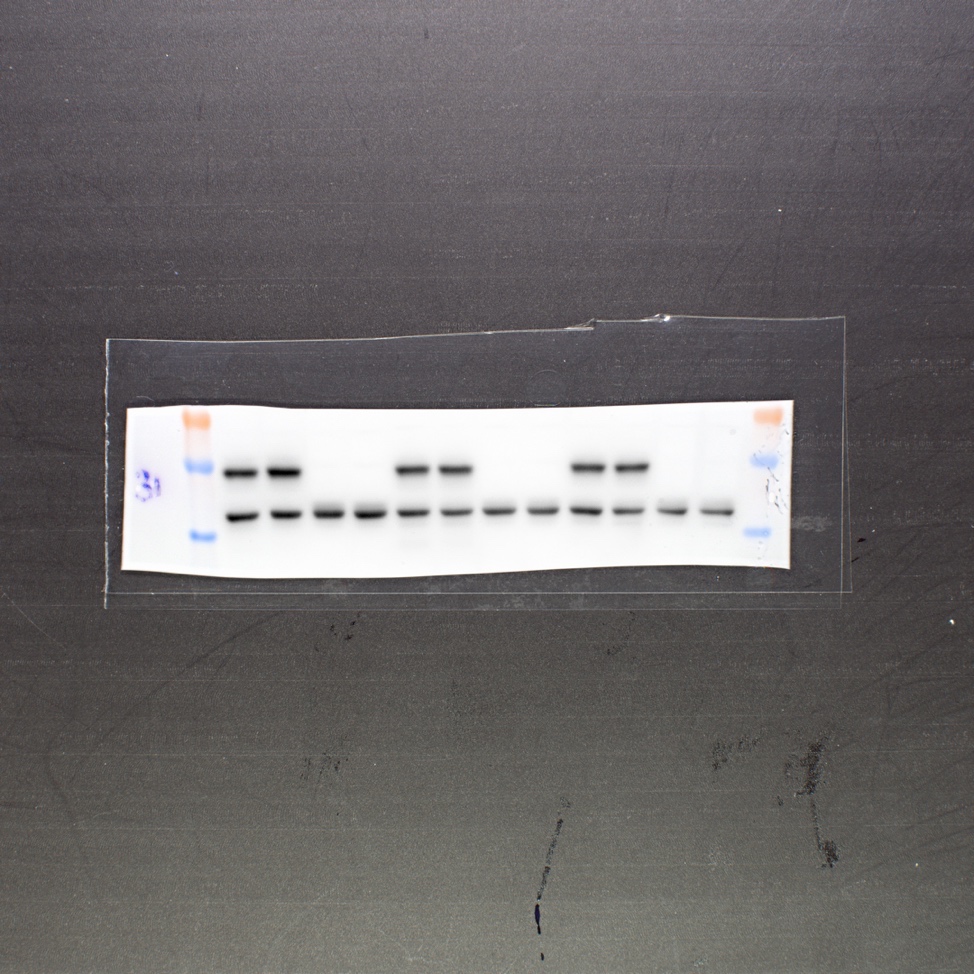

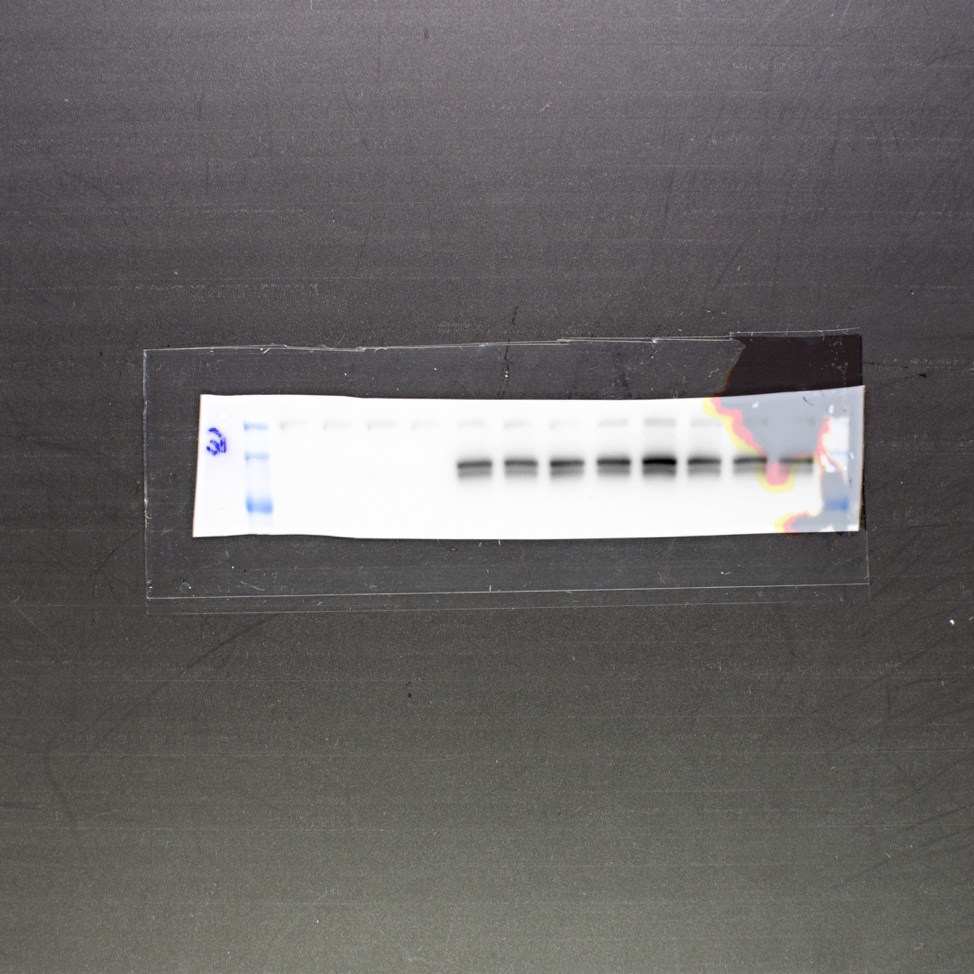
**

β-Actin

P53

ARNT

HIF1A

**
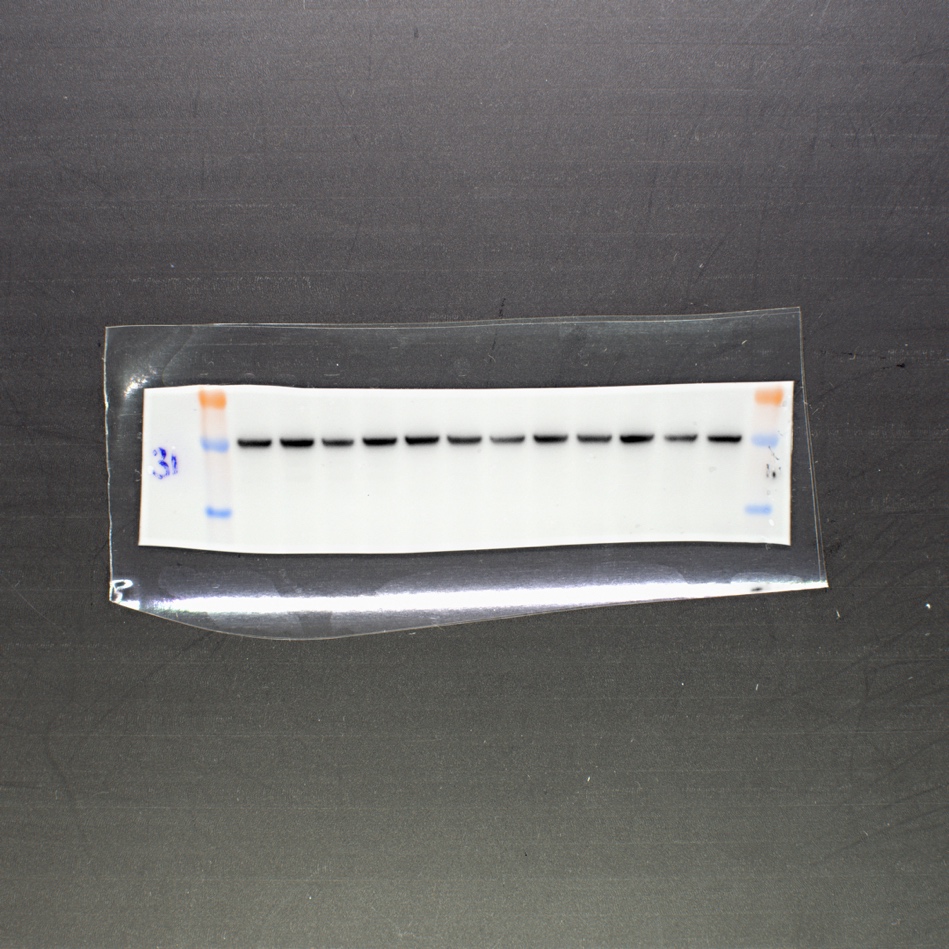
**

α-Tubulin

V3 p53 ko 24 hours (Example Blot in Fig. 3B)

**
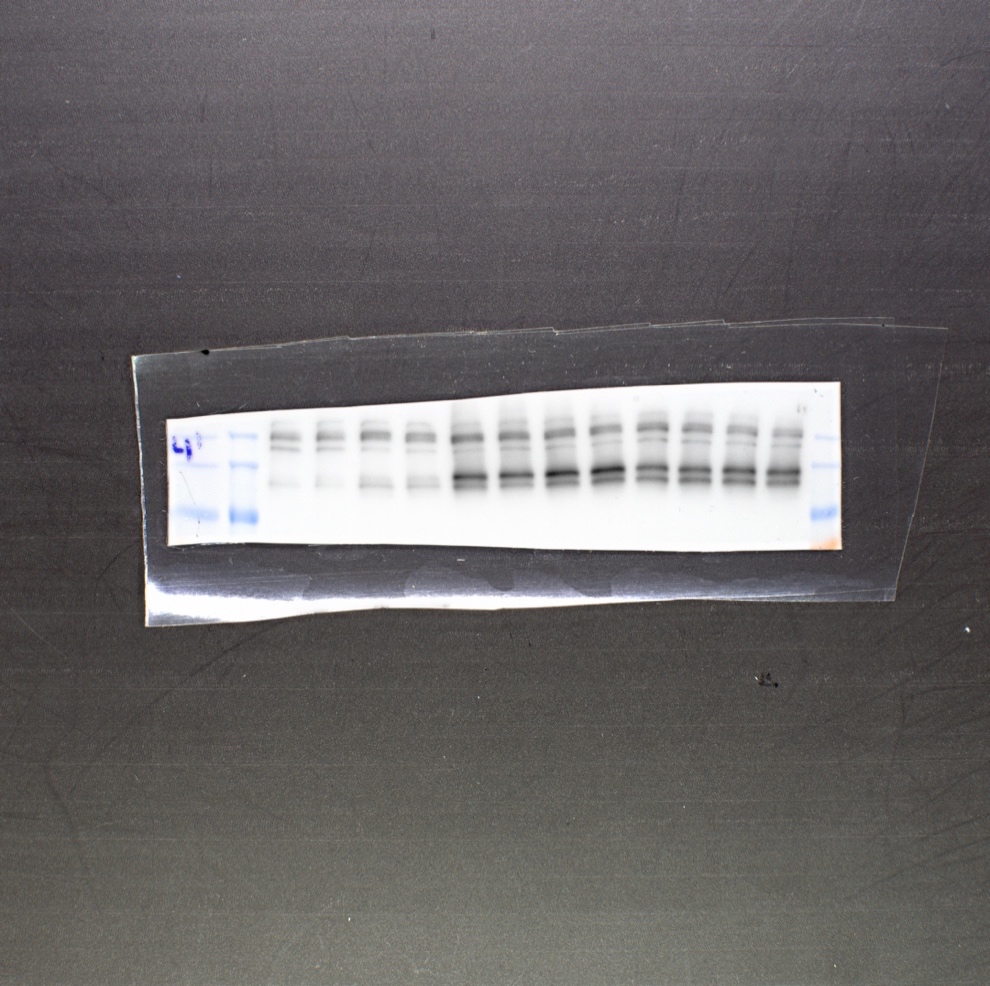
**

HIF1A

ARNT

α-Tubulin

P53

β-Actin

P53

ARNT

V4 p53 ko 4 hours

HIF1A

α-Tubulin

V4 p53 ko 24 hours

P53

HIF1A

ARNT

α-Tubulin

V5 p53 ko 4 hours

HIF1A

ARNT

α-Tubulin

P53

V5 p53 ko 24 hours

HIF1A

ARNT

P53

α-Tubulin

β-Actin

# Western Blots for Figure 8 B

V1 HIF1A ko 4 hours

HIF1A

α-Tubulin

P53

V1 HIF1A ko 24 hours

HIF1A

α-Tubulin

P53

V2 HIF1A ko 4 hours

P53

β-Actin

residue after stripping

α-Tubulin

V2 HIF1A ko 24 hours

α-Tubulin

P53

P53

V3 HIF1A ko 4 hours

HIF1A

α-Tubulin

V3 HIF1A ko 24 hours

α-Tubulin

P53

V4 HIF1A ko 4 hours

HIF1A

α-Tubulin

P53

V4 HIF1A ko 24 hours

HIF2A

α-Tubulin

P53

V5 HIF1A ko 4 hours

HIF1A

P53

α-Tubulin

V5 HIF1A ko 24 hours

P53

HIF1A

α-Tubulin

V6 HIF1A ko 4 hours

HIF1A

P53

α-Tubulin

V7 HIF1A ko 4 hours

HIF1A

P53

α-Tubulin
